# Waning of first- and second-dose ChAdOx1 and BNT162b2 COVID-19 vaccinations: a pooled target trial study of 12.9 million individuals in England, Northern Ireland, Scotland and Wales

**DOI:** 10.1093/ije/dyac199

**Published:** 2022-10-22

**Authors:** Steven Kerr, Stuart Bedston, Declan T Bradley, Mark Joy, Emily Lowthian, Rachel M Mulholland, Ashley Akbari, F D Richard Hobbs, Srinivasa Vittal Katikireddi, Simon de Lusignan, Igor Rudan, Fatemeh Torabi, Ruby S M Tsang, Ronan A Lyons, Chris Robertson, Aziz Sheikh

**Affiliations:** Centre for Medical Informatics, Usher Institute, The University of Edinburgh, Edinburgh, UK; Population Data Science, Swansea University Medical School, Swansea University, Swansea, UK; School of Medicine, Dentistry and Biomedical Sciences, Queen’s University Belfast, Belfast, UK; Public Health Agency, Belfast, UK; Nuffield Department of Primary Care Health Sciences, University of Oxford, Oxford, UK; Population Data Science, Swansea University Medical School, Swansea University, Swansea, UK; Department of Education and Childhood Studies, Swansea University, Swansea, UK; Centre for Medical Informatics, Usher Institute, The University of Edinburgh, Edinburgh, UK; Population Data Science, Swansea University Medical School, Swansea University, Swansea, UK; Nuffield Department of Primary Care Health Sciences, University of Oxford, Oxford, UK; MRC/CSO Social & Public Health Sciences Unit, University of Glasgow, Glasgow, UK; Nuffield Department of Primary Care Health Sciences, University of Oxford, Oxford, UK; Centre for Medical Informatics, Usher Institute, The University of Edinburgh, Edinburgh, UK; Population Data Science, Swansea University Medical School, Swansea University, Swansea, UK; Nuffield Department of Primary Care Health Sciences, University of Oxford, Oxford, UK; Population Data Science, Swansea University Medical School, Swansea University, Swansea, UK; Public Health Scotland, Glasgow, UK; Department of Mathematics and Statistics, University of Strathclyde, Glasgow, UK; Centre for Medical Informatics, Usher Institute, The University of Edinburgh, Edinburgh, UK; BREATHE—The Health Data Research Hub for Respiratory Health, The University of Edinburgh, Edinburgh, UK

**Keywords:** COVID-19, vaccine effectiveness, vaccine waning

## Abstract

**Background:**

Several SARS-CoV-2 vaccines have been shown to provide protection against COVID-19 hospitalization and death. However, some evidence suggests that notable waning in effectiveness against these outcomes occurs within months of vaccination. We undertook a pooled analysis across the four nations of the UK to investigate waning in vaccine effectiveness (VE) and relative vaccine effectiveness (rVE) against severe COVID-19 outcomes.

**Methods:**

We carried out a target trial design for first/second doses of ChAdOx1(Oxford–AstraZeneca) and BNT162b2 (Pfizer–BioNTech) with a composite outcome of COVID-19 hospitalization or death over the period 8 December 2020 to 30 June 2021. Exposure groups were matched by age, local authority area and propensity for vaccination. We pooled event counts across the four UK nations.

**Results:**

For Doses 1 and 2 of ChAdOx1 and Dose 1 of BNT162b2, VE/rVE reached zero by approximately Days 60–80 and then went negative. By Day 70, VE/rVE was –25% (95% CI: –80 to 14) and 10% (95% CI: –32 to 39) for Doses 1 and 2 of ChAdOx1, respectively, and 42% (95% CI: 9 to 64) and 53% (95% CI: 26 to 70) for Doses 1 and 2 of BNT162b2, respectively. rVE for Dose 2 of BNT162b2 remained above zero throughout and reached 46% (95% CI: 13 to 67) after 98 days of follow-up.

**Conclusions:**

We found strong evidence of waning in VE/rVE for Doses 1 and 2 of ChAdOx1, as well as Dose 1 of BNT162b2. This evidence may be used to inform policies on timings of additional doses of vaccine.

Key Messages

- We undertook an observational epidemiological analysis of the effectiveness of COVID-19 vaccines across all four nations of the UK, pooling data from a UK cohort of 12.9 million individuals.
- We carried out a target trial study of waning in vaccine effectiveness (VE)/relative vaccine effectiveness (rVE) against COVID-19 hospitalization or death for first/second doses of ChAdOx1 (Oxford–AstraZeneca) and BNT162b2 (Pfizer–BioNTech).
- For Doses 1 and 2 of ChAdOx1, as well as Dose 1 of BNT162b2, VE/rVE reached zero by approximately Days 60–80 after vaccination, whereas for Dose 2 of BNT162b2, rVE remained above zero throughout 98 days of follow-up.
- Our methodology provides proof of concept for carrying out pooled studies where data are stored in different locations and sharing of individual-level data is not permitted.

## Introduction

In December 2019, a novel coronavirus (SARS-CoV-2) emerged in Wuhan, China.^1^ The World Health Organization declared the outbreak a Public Health Emergency of International Concern on 30 January 2020 and then a pandemic on 11 March 2020. In the UK as of 28 June 2022, there have been >22 million reverse-transcriptase polymerase chain reaction (RT-PCR)-confirmed COVID-19 cases, >880 000 COVID-19 hospitalizations and >170 000 COVID-19 deaths.^2^

COVID-19 vaccines have been developed in record time. Three vaccines have thus far been administered at scale in the UK: ChAdOx1 nCoV-19 (Oxford–AstraZeneca, hereafter ChAdOx1), BNT162b2 (Pfizer–BioNTech, hereafter BNT162b2) and mRNA-1273 (Moderna). These vaccines have demonstrated high levels of effectiveness against a number of outcomes including infection, hospitalization and death, in both clinical trials and observational epidemiological studies.^3–7^

However, there is evidence that vaccine protection against SARS-CoV-2 infection and severe outcomes wanes notably over time. A study of the workforce of the University of California San Diego Health found that the effectiveness of two doses of BNT162b2 or mRNA-1273 against symptomatic infection reduced to 65% after 4 months.^8^ A cross-country study found evidence of waning in effectiveness of second-dose ChAdOx1 against severe COVID-19 outcomes, with vaccine effectiveness (VE) of 63.7% in Scotland and 42.2% in Brazil at 18–19 weeks after the second dose.^9^ A meta-analysis of 18 studies on VE found that protection against severe COVID-19 disease decreased on average by 10.0% between 1 and 6 months after full vaccination.^10^

It is important to determine the timescale over which vaccines provide high levels of protection in order to inform policymaking regarding dosing schedules. To our knowledge, studies from the UK have hitherto analysed data from a single country, e.g. Scotland^9^ or England,^11^ with no multi-nation studies across the UK. The aim of this study was to investigate waning in VE/relative VE (rVE) against severe COVID-19 outcomes using pooled data from across the four nations of the UK using a target trial approach.

## Methods

### Study design and population

We carried out a target trial analysis. Target trials attempt to emulate a clinical trial by finding naturally occurring exposure groups.^12^ Individuals exposed to a dose of vaccine were statistically matched 1:1 to unexposed individuals based on a number of clinical and demographic characteristics. Differences in the composite outcome variable of COVID-19 hospitalization or death were then compared between these groups.

**Figure 1:**
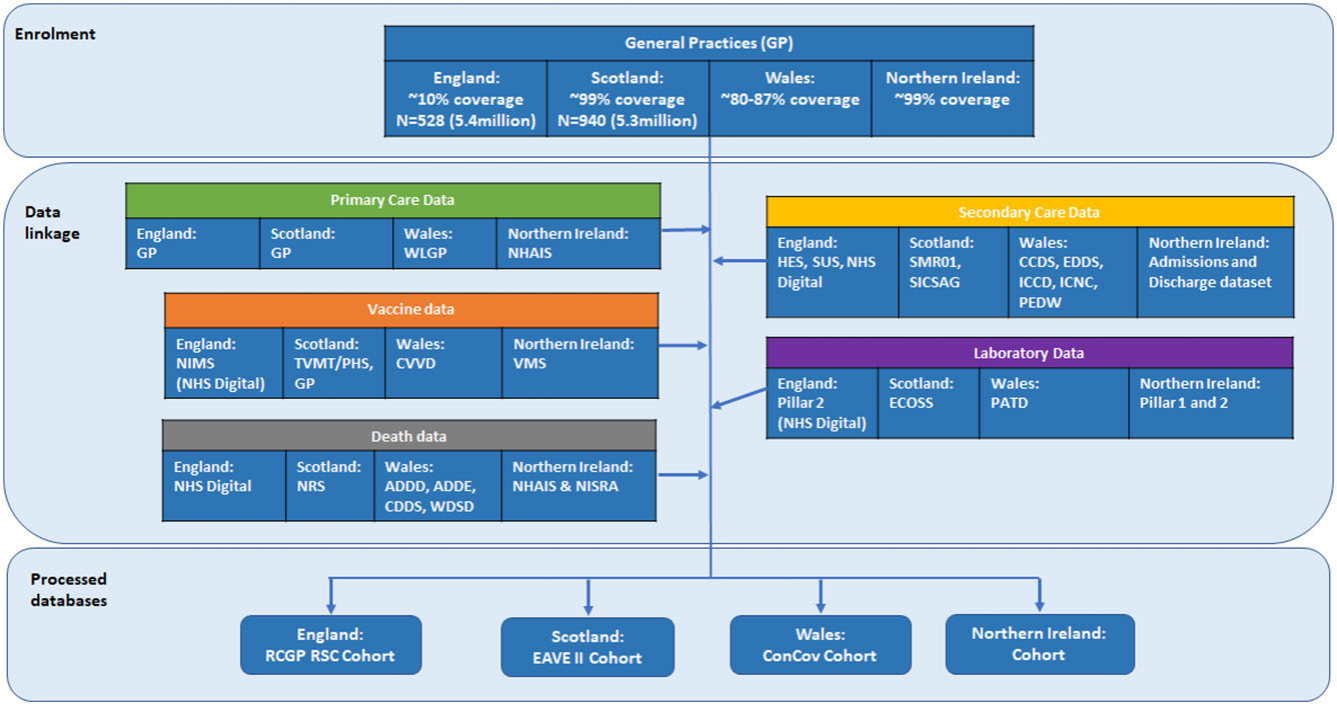
Data linkage diagram. ADDD, Annual District Death Daily; ADDE, Annual District Death Extract; CCDS, Critical Care Dataset; CDDS, COVID-19 Consolidated Deaths; ConCov, Controlling COVID-19 through enhanced population surveillance and intervention; CVVD, Covid Vaccination Dataset; EAVE, Early Assessment of Vaccine and antiviral Effectiveness; ECOSS, Electronic Communication of Surveillance in Scotland; EDDS, Emergency Department Dataset; HES, Hospital Episode Statistics; ICCD, Intensive Care National Research and Audit Centre, Weekly Covid-only; ICNC, Intensive Care National Research and Audit Centre; NHAIS, National Health Application and Infrastructure Services; NHS, National Health Service; NIMS, National Immunisation Management System; NISRA, Northern Ireland Statistics and Research Agency; NRS, National Records of Scotland; ONS, Office for National Statistics; PATD, Pathology COVID-19 Daily; PEDW, Patient Episode Database for Wales; PHS, Public Health Scotland; RCGP RSC, Oxford-Royal College of General Practitioners Research and Surveillance Centre; SICSAG, Scottish Intensive Care Society Audit Group; SMR01, Scottish Morbidity Records 01; SUS, Secondary Users Service; TVMT, Turas Vaccine Management Tool; VMS, Vaccine Management System; WDSD, Welsh Demographic Service Dataset

We followed a pre-specified statistical analysis plan (Supplementary File S1, Statistical analysis plan, available as Supplementary data at *IJE* online). Initially, we sought to study only first-dose waning. However, navigating permissions to undertake this analysis across national trusted research environments (TREs) took far longer than anticipated and by the time we were able to carry out the analysis, many people had received their second dose. Therefore, we also studied second dose and we looked at a longer study period than was originally specified in the statistical analysis plan. We initially planned to do a secondary cohort analysis in order to make use of the full data set. However, as we navigated permissions to undertake this work it became clear that it would not have been possible to do a pooled cohort study as this would require sharing non-count data. Therefore, we carried out the primary target trial analysis. We also planned to use two separate binary definitions of vaccine waning. However, after conducting the analysis, we decided that it would be most informative to present our main results graphically rather than focus on a binary definition. The data sets consisted of linked primary care, secondary care, mortality and virological testing data stored in secure TREs in each of England, Northern Ireland, Scotland and Wales (Figure 1). These data were deterministically linked using unique, anonymized patient identifiers—National Health Service (NHS) number in England, Health and Care Number in Northern Ireland and Community Health Index number in Scotland. In Wales, a combination of deterministic linkage based on NHS number and probabilistic linkage based on personal identifiers was used. The study period was 8 December 2020 to 30 June 2021. Anyone whose most recently recorded age at the cohort start date was aged <18 years was excluded. We also excluded elderly care home residents because care home residence may have been associated with strong confounding effects and there were very few care home residents who were suitable to be used as controls because this population was a high priority group for vaccination.

### Exposure

We defined an individual as exposed according to the type of vaccine and dose number they received, starting from the 14 days after it was administered.

### Outcomes

The primary outcome was a composite of incident COVID-19 hospitalization or death. In England, Scotland and Wales, COVID-19 hospitalization was an RT-PCR-confirmed positive test for SARS-CoV-2 in the 28 days prior to admission or hospitalization with an International Classification of Diseases (ICD-10) code for COVID-19 in any diagnostic position. ICD-10 codes for COVID-19 were U07.1 and U07.2. In Northern Ireland, COVID-19 hospitalization was an RT-PCR-confirmed positive test for SARS-CoV-2 from 14 days prior to admission to 7 days after admission or hospitalization with an ICD-10 code for COVID-19 in any diagnostic position. COVID-19 death was defined as death within 28 days of a positive RT-PCR test for SARS-CoV-2 infection or death with COVID-19 as the underlying ICD-10 cause of death recorded on the death certificate. We selected the first event for each person in the study period. The event date was whichever came first out of the date of hospital admission or the date of death.

### Statistical analysis

In Scotland and Wales, only the body mass index (BMI) variable had missing values and these were imputed using ordinary least squares regression. In Northern Ireland and England, a complete case analysis was done.

We used time-varying matching in approximately 1-month-long intervals to construct the exposure groups of the target trial. Administration of vaccines in the UK started on 8 December 2020 for BNT162b2 and 4 January 2021 for ChAdOx1. Therefore, we took the interval between these two dates to be the first matching period for the propensity model and then full months thereafter. Within each time period, a propensity score for receiving the next vaccine dose was calculated. For the first-dose analysis, individuals who received the first dose of a given vaccine type were matched with individuals who were unvaccinated on the date the vaccine was administered. For the second-dose analysis, individuals who received the second dose of a given vaccine type were matched with individuals who had received only one dose of the same vaccine type at that time.

Analysts in each nation had full access to that nation’s data. In all nations, the following predictors were included in the propensity model: sex, age, local authority area, urban/rural classification,^13–15^ quantiles of multiple deprivation index,^16–19^ number of previous SARS-CoV-2 tests pre-vaccination, mean household age, number of people in household, presence in hospital for any reason 4 weeks prior to the matching period and positive RT-PCR test at any time prior to the matching period. In England, Scotland and Wales, smoking status, BMI and number of QCovid risk groups were also included in the propensity model. QCovid is a tool for predicting the risk of COVID-19 hospitalization and death that takes into account a range of demographic and clinical variables and has been used to inform policy deliberations on shielding and vaccine prioritization in England.^20^ In Northern Ireland, the number of chapters of the British National Formulary (BNF) from which individuals received repeat prescriptions prior to the vaccination programme was included as a proxy for co-morbidity. To be included in the BNF prescription count, a medicine had to be prescribed in both of two 3-month periods in the 6 months before the start of the vaccination programme in the UK, 8 December 2020. Medications related to contraceptives (BNF chapter 7, section 3) were removed as these do not indicate an illness. This method was adapted from one validated in other multimorbidity studies using administrative data.^21^

Within each nation and for each dose, individuals were matched on their propensity to get vaccinated, area of residence and age. Matching on vaccination propensity was by single percentile bands of the propensity score and matching on age was by individual years up to 79 years, then 2-year bands from ages 80 to 89 years, 5-year bands for those aged 90–99 years and one final band for all those aged ≥100 years. Due to rapid uptake, an exception was made for second-dose analysis in Wales, where matching was based on two-percentile propensity bands, by year of age up to 59 years and 5-year bands for 60–99 years and all those aged ≥100 years. Whenever a case had more than one candidate control, one was selected at random, with the restriction that an individual could only be used as a control once. Matching was assessed using covariate balance plots.

Individuals were censored if they had a non-COVID-19 death. Matched pairs were jointly censored whenever either individual received the next dose of the vaccine.

We fitted Poisson models for each exposure group to estimate the rate of incident events. The Poisson model included a spline in days since start of follow-up, an offset for total person-days of follow-up and a vaccination exposure group as a stratification variable. We used this model to predict event rates for each exposure group by day of follow-up. We then divided these rates to obtain rate ratios. Under the model assumptions, the logarithm of this rate ratio is asymptotically normally distributed with variance that can be calculated from the covariance matrix of the parameter estimates. We used this to construct 95% CIs. VE was calculated as 1 – (Rate Ratio). We also fit a second model with a quadratic in time instead of a spline and we tested whether the coefficient on the squared term was equal to zero for the exposed group in order to assess waning. This analysis was repeated in each nation of the UK.

The data governance procedure of the TREs in each country did not allow individual-level data to be shared. However, we obtained permission from data controllers in each nation to confidentially share count data with the Scottish TRE on the condition that these would be combined in a pooled count. In each nation’s TRE, counts of outcome events and person-days of follow-up were collated by vaccine type, target trial arm, age group (18–64, 65–79, 80+ years) and day of follow-up. These were then gathered in the Scottish TRE and summed across the four nations. A similar analysis was then carried out on these pooled data. The target trial design meant that confounders could be controlled for by matching without each nation having to share individual-level data and the Poisson modelling strategy meant that only counts of events and person-years were required, thus allowing a pooled analysis to be carried out sharing only count data.

### Reporting

This study is reported in accordance with the REporting of studies Conducted using Observational Routinely-collected Data (RECORD) guidelines (Supplementary File S2, Checklist, available as Supplementary data at *IJE* online).^22^^,^^23^

## Results

Supplementary Tables S3a–d in Supplementary File S3 (Cohort summary tables, available as Supplementary data at *IJE* online) show the marginal distributions of a number of characteristics in each country’s cohort. Clinical risk groups in these tables were derived from the QCovid algorithm.^20^ There were no notable differences in the marginal distributions of these characteristics by country with the exception of BMI, with Scotland tending to have a greater proportion of the cohort in the overweight category and fewer in the obese category compared with England and Wales (BMI data were not available for Northern Ireland). Tables S4a–d in Supplementary File S4 (Event count tables, available as Supplementary data at *IJE* online) give event counts by vaccination status in each nation.

Table 1 shows the number and proportion of individuals who were exposed to each vaccine dose that were successfully matched with a control by nation. The proportion of exposed individuals that were matched ranged from ∼45–58% with the exception of second-dose matching in Wales (17%). Descriptive tables of matched exposure groups for each country are given in Tables S5a–h in Supplementary File S5 (Exposure group summary tables, available as Supplementary data at *IJE* online). The lower matching rate for the second-dose analysis in Wales was likely due to especially rapid vaccine uptake and the restriction that an individual could only be used as a control once. Covariate balance plots by vaccine type, dose and country are provided in Supplementary File S6 (Covariate balance, available as Supplementary data at *IJE* online).

**Table 1:** Number of exposed individuals who were successfully matched

	First-dose vaccinated	First-dose matched	Second-dose vaccinated	Second-dose matched
England	4 223 375	2 283 348 (54.1%)	3 632 725	1 658 061 (45.6%)
Northern Ireland	1 105 511	640 964 (58.0%)	844 018	393 820 (46.7%)
Scotland	3 481 808	1 650 088 (47.4%)	2 582 105	1 358 286 (52.6%)
Wales	1 629 997	912 704 (56.0%)	1 239 608	216 608 (17.5%)

**Figure 2:**
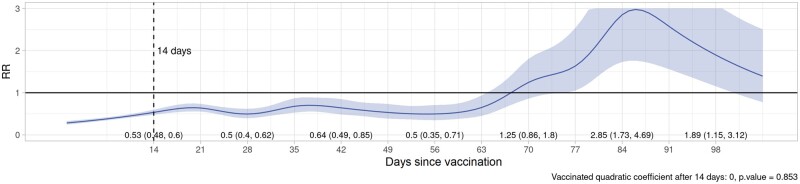
Pooled rate ratios for COVID-19 hospitalization or death, first-dose ChAdOx1 (Oxford–AstraZeneca), all ages

**Figure 3:**
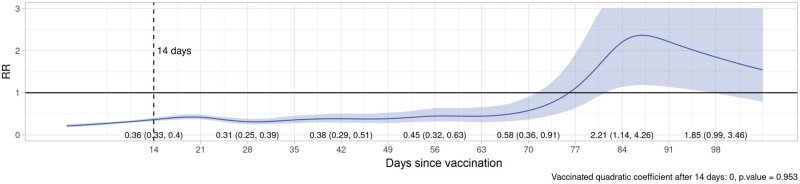
Pooled rate ratios for COVID-19 hospitalization or death, first-dose BNT162b2 (Pfizer–BioNTech), all ages

**Figure 4:**
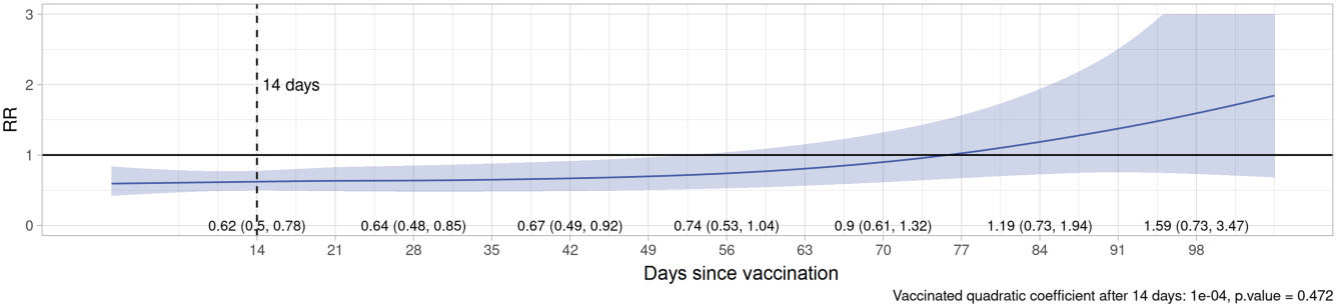
Pooled rate ratios for COVID-19 hospitalization or death, second-dose ChAdOx1 (Oxford–AstraZeneca), all ages

**Figure 5:**
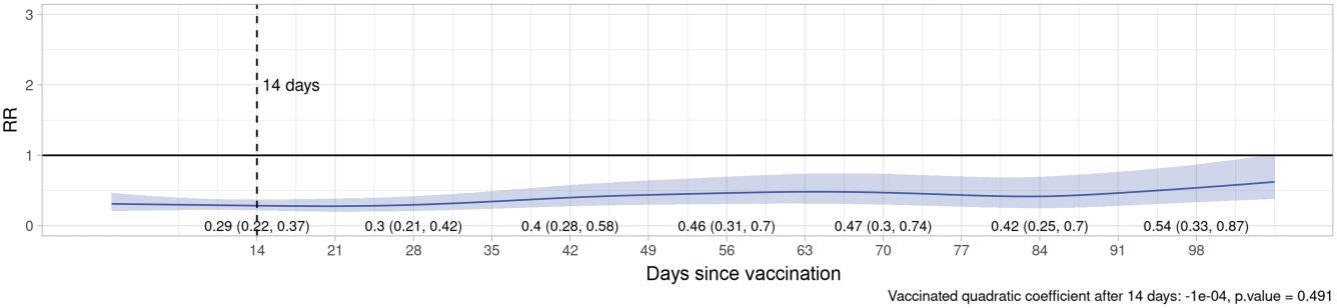
Pooled rate ratios for COVID-19 hospitalization or death, second-dose BNT162b2 (Pfizer–BioNTech), all ages

Plots of VE/rVE over time by vaccine type, dose and country are provided in Supplementary File S7 (VE/rVE by country, available as Supplementary data at *IJE* online). Table 2 and Figures 2–5 show VE/rVE in the pooled analysis, as well as *P*-values for testing that the coefficient on the squared term in time is zero for the exposed group in the model that included a quadratic in time. VE/rVE reached zero by approximately Days 60–80 for Doses 1 and 2 of ChAdOx1 and Dose 1 of BNT162b2. rVE for Dose 2 of BNT162b2 remained above zero throughout 98 days of follow-up. However, in the models that included quadratic terms in time, the *P*-value for the coefficient on the squared terms for the exposed group were quite high for both vaccines and doses, ranging from 0.47 to 0.95. Figure S8a–l in the Supplementary File S8 (Pooled VE/rVE by age group, available as Supplementary data at *IJE* online) show pooled VE/rVE stratified by age group (18–64, 65–79, 80+ years). These results were qualitatively similar, but estimates were less precise than the combined analysis for all age groups.

**Table 2:** Vaccine effectiveness and relative vaccine effectiveness in pooled study

Day	First dose ChAdOx1^a^	Second dose ChAdOx1^a^	First dose BNT162b2^b^	Second dose BNT162b2^b^
14	47 (50 to 52)	38 (22 to 50)	64 (60 to 67)	71 (63 to 78)
28	50 (38 to 60)	36 (15 to 52)	69 (61 to 75)	70 (58 to 79)
42	36 (15 to 51)	33 (8 to 51)	62 (49 to 71)	60 (42 to 72)
56	50 (29 to 65)	26 (–4 to 47)	55 (37 to 68)	54 (30 to 69)
70	–25 (–80 to 14)	10 (–32 to 39)	42 (9 to 64)	53 (26 to 70)
84	–185 (–73 to –369)	–19 (–94 to 27)	–121 (–14 to –326)	58 (30 to 75)
98	–89 (–15 to –212)	–59 (–247 to 27)	–85 (1 to 46)	46 (13 to 67)

a Oxford–AstraZeneca.

b Pfizer–BioNTech.

## Discussion

We carried out a pooled epidemiological analysis of linked, pseudonymized national-level vaccination data across the four nations of the UK. We employed a novel methodology to allow a pooled study to be done with only count data being shared between each country’s TREs. We found evidence of waning in VE/rVE for Doses 1 and 2 of ChAdOx1 and Dose 1 of BNT162b2, with VE/rVE dropping to zero ∼60–80 days after the date of administration and becoming negative thereafter. Our rVE estimates for Dose 2 of BNT162b2 remained above zero throughout 98 days of follow-up.

We believe that the most likely explanation for negative VE/rVE is that vaccination caused recipients to believe they were protected, leading them to change their behaviour in ways that increase their chance of contracting the infection. These changes in behaviours should initially have been outweighed by the protection offered by the immune response stimulated by the vaccine, but as time progressed the protection is likely to have diminished such that the impact of behavioural changes may have become dominant. It is also possible that naturally acquired immunity provides more robust protection than vaccination.^24^ Our VE/rVE estimates were lower than estimates of vaccine efficacy seen in clinical trials. Clinical trials for both ChAdOx1 and BNT162b2 reported 100% efficacy against severe COVID-19 outcomes after a two-dose regimen.^5^^,^^6^ There are several possible explanations for this. These clinical trials were carried out during different time periods and in different populations compared with our study. In particular, the dominant variants in circulation were not the same. The clinical trials included a blinding procedure, where the control group was given a placebo injection. Our study examined real-world effectiveness, where a placebo was not administered. This could have led to behavioural confounding that might be expected to make VE estimates lower than vaccine efficacy. Our first-dose VE estimates tended to be lower compared with other estimates in the literature.^25^ One major difference is that we used a target trial methodology as opposed to other common observational epidemiological designs such as cohort and case–control studies. We have previously carried out a cohort study of vaccine waning for two doses of ChAdOx1 on the same EAVE II data set, in tandem with a separate data set from Brazil.^9^ Our rVE estimates are consistent with results from this previous study, as well as a cohort study of second-dose waning of BNT162b2 in Israel.^26^ Our results may also be broadly consistent with a target trial study of VE of BNT162b2 in Israel, although a direct comparison cannot be made for second dose because we estimated VE relative to those who had received a first dose, as opposed to the unvaccinated.^27^ A key strength of this paper is the use of the target trial method, which seeks to emulate a clinical trial by finding naturally occurring exposure groups in the population. This design together with the Poisson regression modelling strategy meant that the only data that were required in the analysis were count-level data from the exposure groups in each nation. We obtained permission to confidentially share counts of individuals stratified by categories between TREs in each nation on the understanding that these would be combined in a pooled count. This enabled us to conduct a pooled analysis across the four nations of the UK. Typically, the only way to combine results from separate analyses on individual-level data that cannot be shared is to do a meta-analysis. Our use of splines in time allowed us to model complex trajectories for VE/rVE. A final strength of this paper is that we used time-varying, incident density sampling in our propensity score matching procedure to account for changes in vaccine prioritization over time.

A limitation in our analysis is that there was limited follow-up in populations that were prioritized for vaccination, particularly the elderly. Matched pairs in older age groups tended to be censored relatively quickly, contributing to imprecision in our estimates of VE/rVE. This was particularly true in the second-dose analysis in Wales, for which the matching percentage was only 17% due to an especially rapid rollout there. However, Scotland and England contributed most of the data that were used in the pooled analysis and data from Wales likely had a relatively minor effect on the results. We considered allowing individuals to be used multiple times in the matching, but we believe this would have led to unacceptable distortion of CIs on our estimates. Target trials tend to offer better control over confounders at the cost of lower statistical power and it is not uncommon for a significant proportion of the study population to be lost when creating exposure groups in target trials. There was also a potential selection bias in that those who are matched are not representative of those who are vaccinated. Comparison of Table S3 with S5 (available as Supplementary data at *IJE* online) provides information on this and generally there is reasonable agreement. It is possible that younger people were more likely to be matched and this may have been due to the elderly being a smaller group that was prioritized for vaccination. It is not clear in which direction this bias may have worked. Whereas England, Scotland and Wales had access to QCovid risk group variables for use in the propensity matching, Northern Ireland did not and BNF chapters prescribed was used as a proxy. Although pooling data across the nations noticeably improved the precision in our estimates, there was still significant uncertainty, particularly in the age-stratified analyses and at large times elapsed since exposure. The Alpha variant was dominant in the UK until May 2021, switching to the Delta variant thereafter. This change in the dominant variant part-way through our study period meant it was difficult to determine the extent to which waning in VE was due solely to the passage of time vs the emergence of new variants and potential vaccine escape. Our analysis of first-dose waning indicated that VE changes non-trivially with time. In the second-dose analysis, we considered matching exposure group pairs by date of first-dose vaccination to control for VE of the first dose changing with time. However, this resulted in very few matches and very little follow-up time. We believe it is likely that matching by propensity score for vaccination controlled adequately for this confounder because the dosage schedule was similar for most people who were vaccinated. Our estimates of VE/rVE against COVID-19 hospitalization or death tended to be notably high immediately after vaccination, on a timescale that was too short to be plausibly explained by the immune response generated by the vaccine. This may have been due to behavioural changes associated with the vaccination programme. People who contracted COVID-19 prior to a scheduled vaccination may have postponed vaccination, causing early VE/rVE to be biased upwards. In addition, some recipients may have suffered mild illness following vaccination, causing them to reduce their social contact, reducing the chance of contracting COVID-19. Other behavioural explanations have also been proposed for this phenomenon.^28^ On the other hand, it is possible that there were people who had an event shortly after vaccination but were infected prior to vaccination. This effect would work in the opposite direction, lowering VE/rVE estimates in a period immediately following vaccination. Finally, it is possible that there was residual confounding that was not fully accounted for by the target trial study design.

This paper contributes additional robust evidence on the waning of VE/rVE for first and second doses of ChAdOx1 and BNT162b2 vaccines. In particular, if protection against severe COVID-19 wanes over the course of months, then COVID-19 vaccination may have greatest utility as a tool for ‘flattening the curve’. Although we studied first- and second-dose vaccination only, the evidence here may still be informative for future doses of vaccine. We have also used a novel methodology that allowed us to carry out a pooled study—for the first time—across all UK nations without having to share individual-level data. This will we hope serve as a proof of concept for further pooled multicentre/country studies in the future.

## Ethics approval

In England, approvals were obtained from the Health Research Authority, London Central (reference number 21/HRA/2786). In Scotland, data approvals were obtained from the National Research Ethics Service Committee, Southeast Scotland 02 (reference number: 12/SS/0201) and the Public Benefit and Privacy Panel for Health and Social Care (reference number: 1920–0279). In Northern Ireland, approval was by the Honest Broker Service Governance Board (Project 064). In Wales, approval was provided by Secure Anonymised Information Linkage (SAIL) independent Information Governance Review Panel (IGRP) (Project 0911).

## Supplementary Material

dyac199_Supplementary_Data

## Data Availability

All code, metadata and documentation for this project is publicly available at https://github.com/EAVE-II/Covid-vaccine-waning-pooled. Most of the data that were used in this study are highly sensitive and will not be made available publicly.
