# Supplementary material for: Waning of first- and second-dose ChAdOx1 and BNT162b2 COVID-19 vaccinations: a pooled target trial study of 12.9 million individuals in England, Northern Ireland, Scotland and Wales
Source: Int J Epidemiol. 2022 Oct 22;52(1):22–31. doi: 10.1093/ije/dyac199 (PMC9620314; doi:10.1093/ije/dyac199)
Supplement: dyac199_Supplementary_Data [file dyac199_supplementary_data.zip › dyac199_Supplementary_Data/ije-2022-04-0492-File008.docx]

**S2 Statistical analysis plan.**


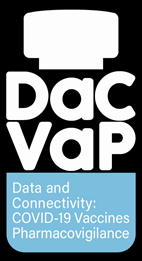
**DaC-VaP**

**Analysis plan to investigate effectiveness of first dose vaccine over time in adults using linked UK national data**

| **Full Project Title** | DaC-VaP |
| --- | --- |
| **Version Number** | V3 |
| **Previous Versions** | N/A |
| **Effective Date** | 05 March 2021 |
| **Analyst(s)** | Scotland: Rachel Mulholland (RM)  England: Mark Joy (MJ)  Wales: Emily Lowthian (EL) and Stuart Bedston (SB)  NI: Declan Bradley (DB) and Siobhan Murphy (SM) |
| **Co-authors** | Srinivasa Vittal Katikireddi* (SVK), Chris Robertson (CR), Ting Shi (TS), Eleftheria Vasileiou (EV), Steven Kerr (SK), Aziz Sheikh (AS) |
| **Code** | <https://github.com/EAVE-II/Covid-vaccine-waning-pooled> |

*corresponding author

| **Version History** | | | |
| --- | --- | --- | --- |
| **Version** | **Date** | **Author** | **Notes** |
| V1 | 05.03.2021 | EV, SK | First version sent to team |
| V2 | 09.03.2021 | SVK |  |
| V3 | 15.06.2021 | RM | Update for DaCVap meta-analysis |
|  |  |  |  |
|  |  |  |  |

Contents

[1. Introduction 3](#_Toc80323386)

[2. Aims and objectives 3](#_Toc80323387)

[2.1 Aims 3](#_Toc80323388)

[2.2 Objectives 3](#_Toc80323389)

[3. Study Design 3](#_Toc80323390)

[3.1 Study design 3](#_Toc80323391)

[3.2 Setting 4](#_Toc80323392)

[3.3 Population 4](#_Toc80323393)

[3.4 Data sources 4](#_Toc80323394)

[3.5 Inclusion/exclusion criteria 5](#_Toc80323395)

[4. Data and data validation 5](#_Toc80323396)

[4.1 Data variables available 5](#_Toc80323397)

[4.3 Consistency and error checking 6](#_Toc80323398)

[5. Statistical analyses 7](#_Toc80323399)

[5.1 Exposures of interest 7](#_Toc80323400)

[5.2 Outcomes of interest 7](#_Toc80323401)

[5.3 Potential confounders 7](#_Toc80323402)

[5.4 Potential effect modifiers 7](#_Toc80323403)

[5.5 Analytical techniques 7](#_Toc80323404)

[5.5.1 Descriptive analysis 7](#_Toc80323405)

[5.5.2 Matched analysis 7](#_Toc80323406)

[5.5.3 Cohort analysis 9](#_Toc80323407)

[5.5.4 Sub-group analysis 9](#_Toc80323408)

[5.5.5 Corrections for multiple testing 9](#_Toc80323409)

[5.5.6 Sensitivity analysis 9](#_Toc80323410)

[5.5.7 Other analysis 9](#_Toc80323411)

[5.6 Meta analysis 9](#_Toc80323412)

[5.6.2 Descriptive table on matched cohort 14](#_Toc80323413)

[5.6.3 Covariate balance plot 16](#_Toc80323414)

[5.6.4 Table of risk outcomes stratified by follow up time period 16](#_Toc80323415)

[5.6.5 Plot of GAMs 17](#_Toc80323416)

[5.6.7 Meta analysis methods 18](#_Toc80323417)

[5.7 Missing data 18](#_Toc80323418)

[5.8 Statistical software 18](#_Toc80323419)

[6 Reporting results 18](#_Toc80323420)

[6.1 Reporting guidelines and conventions 18](#_Toc80323421)

[6.2 Dissemination 19](#_Toc80323422)

[References 20](#_Toc80323423)

[Appendix 21](#_Toc80323424)

[Table S1. ICD-10 codes 21](#_Toc80323425)

# 1. Introduction

The aim of this study is to determine whether protection from COVID-19 vaccination wanes after the first SARS-CoV-2 vaccine dose. At present, the vaccination programme in the UK uses two dose regimens of the Pfizer-BioNTech (BNT162b2) and the Oxford-AstraZeneca (ChAdOx1) vaccines. Whilst Moderna vaccine is now available in the UK, we will not include it in this analysis due to later start date and lack of follow-up data coverage. We theorise the vaccination programme as a natural experiment which has impacts that directly arise from the vaccination itself, as well as behavioural and other responses that occur as a consequence of broader programmatic effects - for example, being asked not to attend for vaccination if symptomatic and being reminded about protective behaviours to avoid infection.

We will use pseudonymised individual level linked routinely collected primary, laboratory and vaccination healthcare data across the UK nations. All data and analyses will be hosted within a trusted research environment within each nation. Pooled estimates will be calculated across the UK nations.

# 2. Aims and objectives

## 2.1 Aims

To assess the relationship between time since receiving the first vaccine dose and second dose (of the Pfizer-BioNTech and Oxford-AZ vaccines) compared to unvaccinated and partially vaccinated people, and effectiveness of SARS-CoV-2 vaccines against COVID-19 hospitalisation and death.

## 2.2 Objectives

We seek to:

1. Estimate vaccine effectiveness against the composite outcome of COVID-19 hospitalisation/death as a function of time since first SARS-CoV-2 vaccine dose and pool these estimates for the whole UK population.
2. Assess if vaccine effectiveness as a function of time since first vaccine dose differs by vaccine type, age group and sex.

# 3. Study Design

## 3.1 Study design

The primary study design is an open prospective cohort study with risk-set matching to emulate a ‘target trial’ comparing outcomes amongst first dose vaccinated (partially vaccinated) to people who have not yet been vaccinated [1-3]. To maximise statistical power, we will conduct secondary analyses using the entire study population.

The time period for the study will begin on the first date of vaccine administration in each UK nation, 8^th^ December 2020, and will end on the 30^th^ April 2021.

## 3.2 Setting

UK

## 3.3 Population

Individuals registered in General Practices (GPs) across the UK (~5.3 million for England, approx. 1.4 million for Northern Ireland, ~ 5.4 million for Scotland and ~3.1million for Wales).

## 3.4 Data sources

| **Data** | **Descriptive** | **England** | **Northern Ireland** | **Scotland** | **Wales** |
| --- | --- | --- | --- | --- | --- |
| Primary care data | General Practices (GPs) data for information on demographics, other population characteristics and vaccination data. | RCGP | NA | EAVE II – All GPs in Scotland | Welsh Longitudinal General Practice (WLGP) |
| Vaccination data | COVID-19 vaccines administered in national vaccination centres | GPs and the National Immunisation Management Service (NIMS) | GPs and Health and Social Care (HSC) Trusts via VMS | GPs and Turas Vaccination Management Tool (TVMT) | COVID Vaccination Data (CVVD) population from the all Wales Immunisation System (WIS) |
| Laboratory test data | RT-PCR laboratory confirmed SARS-CoV-2 infection | NHS Digital | Pillar 1 dataset from Laboratory Information System (LIS) and Pillar 2 dataset from NHS Digital | Electronic Communication of Surveillance in Scotland (ECOSS) database | Pillar 1 and 2 data from all NHS and private laboratories within the PATD data source. There is also national lateral flow testing data in CVLF |
| Hospitalisations | COVID-19 hospital admissions | Hospital Episode Statistics (HES), which are the long-term validated record; and Secondary Uses Services (SUS) which is an extract of contemporary operational data which after validation will become HES | Admissions and discharge dataset | Scottish Morbidity Record (SMR) 01 and Rapid Preliminary Inpatient Data (RAPID) | Patient Episode Database for Wales (PEDW) |
| Deaths | COVID-19 deaths | NHS Digital and Office for National Statistics (ONS) | National Health Applications and Infrastructure Services (NHAIS) and the Northern Ireland Statistics and Research Agency (NISRA) death data | National Records of Scotland (NRS) death data | National Population Spine (Welsh Demographic Service – WDSD), ONS death data (ADDE and ADDD) and a national NHS master patient index record (Consolidated Death Data Source – CDDS) |

## 3.5 Inclusion/exclusion criteria

Exclusion criteria:

- Age 17 or less at the study start date (8^th^ December 2020)
- Deceased prior to the study start date (8^th^ December 2020)
- Those living in an elderly care home (household with more than 10 people and an average age of over 65 as proxy)

# 4. Data and data validation

## 4.1 Data variables available

Table 1 lists the groupings of variables available for this study by data source. Exposure data are described in the vaccinations category. Outcome data are described in the secondary care, mortality, and laboratory tests categories. The rest of categories contain data on potential confounding factors and effect modifiers.

**Table 1: Data items/variables and data sources**

| **Data category** | **Data item** | **England** | **Northern Ireland** | **Scotland** | **Wales** |
| --- | --- | --- | --- | --- | --- |
| Demographic | Sex | GP | NHAIS | GP | C19_COHORT20 |
|  | Age | GP | NHAIS | GP | C19_COHORT20 |
| Socioeconomic status (SES) | IMD, SIMD, NIMDM, WIMD | Post code to IMD | NHAIS | GP | C19_COHORT20 |
| Other characteristics | Body Mass Index (BMI) | GP | GP | GP | WLGP, QCOVID |
|  | Smoking | GP | GP | GP | WLGP |
|  | Blood Pressure | GP | NA | GP | WLGP |
| Geographic | Type of settlement (urban/rural) | GP | NHAIS | GP | C19_COHORT20 |
|  | Local authority | GP | NHAIS | GP | C19_COHORT20 |
| Type of residence | Private housing, care home or social housing | GP | NHAIS | GP | C19_COHORT20, CARE |
| Clinical diagnoses | Underlying conditions  (e.g., asthma, cardiac disease etc.) | GP |  | GP | GP, WLGP, PEDW, CVSP, QCOVID, WDDS |
| Household information | Number of people in household, Average age of household (to create proxy for elderly care home) |  |  | Household identifier registry from CHI | C19_COHORT20, CARE |
| Vaccinations | Vaccine type | GP, NIMS | GP, HSC Trusts via VMS | GP, TVMT | CVVD |
|  | Vaccine dose | GP, NIMS | GP, HSC Trusts via VMS | GP, TVMT | CVVD |
|  | Vaccination date | GP, NIMS | GP, HSC Trusts via VMS | GP, TVMT | CVVD |
| Laboratory tests | RT-PCR SARS-CoV-2 test result | GP, Pillar 1&2  SGSS, PHE | Pillar 1 dataset from LIS, Pillar 2 dataset from NHS Digital | ECOSS | PATD (Pillar 1, 2, & 3 data from all NHS and private labs), CVLF testing and results data |
|  | Date of RT-PCR SARS-CoV-2 test |  |  |  |  |
|  | Genome sequencing data |  |  |  |  |
| Secondary care | Hospital admission | GP, SUS | Admissions and discharge dataset | SMR01 | PEDW |
|  | Admission ICD-10 code | GP, SUS | Admissions and discharge dataset | SMR01 | PEDW |
| Mortality | Deaths | GP, ONS, SSRS | National Health Applications and Infrastructure Services (NHAIS) and the Northern Ireland Statistics and Research Agency (NISRA) death data | NRS | C19_COHORT20_MORTALITY |

4.2 Constructed variables

- Time to event in weeks (0-13, 14-20, 21-27, 28-34, 35-41, 42-48, 49-55, 56-62, 63-69 and 70+ days from dose 1, extend to 70-76, 77+ if numbers allow)
- Number of Q Covid risk groups (0,1,2,3,4,5+)
- Number of PCR tests in the pre vaccination era (0,1,2,3-4, 5-9, 10+). This will serve as our proxy for healthcare workers where healthcare worker status is unknown.
- Elderly care home resident (defined as person from a household with more than 10 people and an average age of over 65)

## 4.3 Consistency and error checking

We will check for patterns of missingness and implausible values for all analytical variables being used, with a record maintained of reasons for exclusion of any records from analysis. In the case where a variable of interest has high levels of missingness, we will consider using alternative variables that are closely related as a proxy for these missing data.

# 5. Statistical analyses

## 5.1 Exposures of interest

For the first vaccine dose (partial vaccination), an individual will be defined as exposed from day 14 after receiving the first vaccine dose between the period of 8^th^ December 2020 and until the end of follow up on 30^th^ April 2021.

## 5.2 Outcomes of interest

The primary outcome will be a composite outcome of time to COVID-19 hospitalisation or death. COVID-19 hospitalisation will be defined as a RT-PCR confirmed positive test for SARS-CoV-2 in the 28 days prior to admission, or with ICD-10 code for COVID-19 (in any diagnostic position). COVID-19 deaths will be defined as COVID-19 as the underlying ICD-10 cause of death recorded on the death certificate, or death from any cause within 28 days of a positive RT-PCR test for SARS-CoV-2 infection.

Secondary outcomes will be the single outcomes of: a) COVID-19 hospitalisation (as defined above), and b) COVID-19 deaths.

## 5.3 Potential confounders

Age, sex, socio-economic status (SES), residential settlement measured by the urban/rural 6 fold classification (1 refers to large urban areas and 6 refers to small remote rural areas), household size, number and types of comorbidities commonly associated with COVID-19 illness (asthma, chronic kidney disease, liver cirrhosis, chronic neurological condition, heart failure, diabetes (type 1 and type 2), dementia, coronary heart disease), risk factors (smoking status, blood pressure, body mass index) and geography (local authority level).

## 5.4 Potential effect modifiers

Stratification into different population groups by age group (18-64, 65-79, 80+ years) and sex will be performed. Given that vaccine effectiveness may differ amongst previously infected individuals, we will stratify analyses by previous confirmed infection status when adequate numbers are available for meaningful statistical analysis. As noted above, we will also stratify analyses by vaccine type.

## 5.5 Analytical techniques

### 5.5.1 Descriptive analysis

We will commence analysis by conducting descriptive analyses to visually inspect trends in vaccination delivery, age-specific COVID-19 hospitalisations and COVID-19 deaths, including by age group and sex. This will include inspecting the number of people who have received no doses, one dose and two doses and the length of time between the receipt of one dose and two doses.

### 5.5.2 Matched analysis

To create control groups for our primary analysis, we will emulate a ‘target trial’. We will do this by conducting 1:1 risk-set matching to identify individuals who had not yet become exposed (i.e. remained unvaccinated) on the date an exposed person received their vaccine dose. We will do this using a time-varying propensity score matching algorithm, by fitting separate propensity score models by month.

Due to the strong confounding effect of living in an elderly care home (arising from the targeted vaccination of these residents and their high risks of COVID-19 harms) and the lack of available controls, we will exclude people whose household had more than 10 people with an average age of 65 years and over. This will be used as a proxy for those living in an elderly care home.

We will incorporate the following characteristics into the time-varying propensity score model: sex, age (grouped in 5 year bands), SES, number of risk factors (grouped), number of previous SARS-CoV-2 tests pre-vaccination era (grouped), geography (local authority level), urban-rural settlement, average household age, number of people in household (grouped), smoker indicator, BMI (grouped), presence in hospital status (hospitalised for any reason 4 weeks prior to monthly time-period) and previous positive test indicator (tested positive previous to monthly time-period). Individuals will then be matched using exact matching based on the propensity score in 1% bands, local authority, and age (exact age in years, truncated up to 100 years). The adequacy of the matching will be assessed by checking for imbalance of the individual covariates and the individual risk groups across exposure groups.

We expect that any effects before 14 days for first dose vaccination will reflect programmatic effects (e.g. being instructed not to attend vaccination if symptomatic and reinforcement of behavioural advice to reduce COVID-19 related risks). Follow up will therefore start from day 14 after the date of first dose vaccination (and day 7 after second dose) for both the exposed and control person. It will end for both the exposed and control person on the first of: experiencing the outcome of interest, death (from any cause) or end of follow up period. Unvaccinated controls who become vaccinated will be eligible to become considered as exposed, after follow up is censored for the exposed-unexposed pair at the date of vaccination of the control. Follow-up of the exposure period will also be censored for both the recipient and the control if the partially vaccinated recipient participant received a second dose. Analyses will be stratified by vaccine type.

For the matched cohort analysis, we will draw Kaplan Meier curves to inspect cumulative incidence. We will then fit Poisson regression models to estimate rate ratios (RRs) with 95% confidence intervals (95% CIs) for the association between exposure group and risk of the outcomes, at weekly time periods from exposure to event (Section 4.2).

We will fit a model including a spline with person-time defined in days, to explore the timing of any waning of vaccine effectiveness. The splines will be fit using thin plate regression splines (the R package ‘mgcv’ default settings) with weekly knots from days 14 to the maximum week. The difference between the smooths in the exposure groups will be calculated using methods by Simpson, [7] to obtain smoothed estimated RRs between the exposure groups at daily follow-up periods.

We will assess vaccine waning using two different pre-specified approaches: first, conducting statistical hypothesis tests for declining effectiveness; and second, by assessing whether effectiveness is maintained above a minimum acceptable level. For the former, we will assess the curvature of the association in the spline modelled estimates over time, to evaluate whether the smoothed estimates peak and return to null. This will be achieved by fitting the same statistical model as for the spline but replacing the spline term with a quadratic term and post 14 days only. A statistically significant positive quadratic coefficient from this model in the exposed group would suggest waning. For the latter approach, we adopted the US Food & Drug Administration’s threshold of achieving a RR of at least 0.5 for the point estimate. [8]

### 5.5.3 Cohort analysis

Given the relatively small number of anticipated COVID-19 deaths during the follow up period, the secondary analysis focusing on mortality will be based on data from the whole cohort. In addition, secondary analyses will be conducted for COVID-19 hospitalisation.

Vaccination status (unvaccinated and one dose) will be defined as a time-varying exposure. Poisson regression adjusted for an offset representing the time at risk, with rate ratios (RRs) and 95% confidence intervals (CIs) will be calculated. Vaccine effectiveness and 95% Confidence Intervals (CIs) will be calculated as (1-Rate Ratio)*100. Models will be adjusted for relevant confounders, including age, sex, SES, geography, time period, comorbidities and risk factors. Stratification variables for each week post-vaccination will be included in the Poisson regression. We will assess waning by examining the resulting discrete set of weekly RR estimates. The reason for doing it this way as opposed to including a polynomial/spline in time since vaccination in the Poisson regression is that this would require adding rows to the data for every individual and every time, which would make the dataset impractically large to work with when using the full cohort.

### 5.5.4 Sub-group analysis

Subgroup analyses by vaccine type, age group and sex will be performed. We will consider conducting sub-group analysis by time-period too, especially if there is evidence of different circulating variants predominating.

### 5.5.5 Corrections for multiple testing

N/A

### 5.5.6 Sensitivity analysis

We will consider exploring the impact of alternative approaches to classifying the start of the exposed period (from day 0 of vaccination for both first and second doses, and day 21 after first dose), classifying COVID-19 hospitalisation on the basis of primary diagnosis (rather than any diagnostic position) and using a doubly robust estimator (i.e. including variables for confounder adjustment within regression models that already incorporate a propensity score).

### 5.5.7 Other analysis

We will consider conducting falsification analyses (negative controls) for alternative time periods (e.g. repeating analyses using time periods two months prior to first vaccination dose) to check the comparability of our exposure groups.

## 5.6 Pooled analysis

Results from the described analysis (Section 5.5) will be pooled across the UK nations. All nations should supply the following figures and tables.

5.6.1 Descriptive table of study population

Table of the characteristics of the underlying population (excluding elderly care home residents).

Code: 02_descriptive.R (lines 319-338)

| Characteristic | Level | Unvaccinated | | One dose ChAdOx1 | | Two doses  ChAdOx1 | | One dose BNT162b2 | | Two doses BNT162b2 | |
| --- | --- | --- | --- | --- | --- | --- | --- | --- | --- | --- | --- |
| Total | | | 1750310 | | 1767347 | | 862901 | |  | |  |
| Sex | Female | 803174 (45.9%) | | 532302 (61.7%) | | 911365 (51.6%) | |  | |  | |
|  | Male | 947136 (54.1%) | | 330599 (38.3%) | | 855982 (48.4%) | |  | |  | |
| Age (years) | Mean (SD) | 36 (13.7) | | 55.9 (16.4) | | 58.8 (15.3) | |  | |  | |
|  | Median (IQR) | 34 (16) | | 59 (24) | | 58 (20) | |  | |  | |
| Age group (years) | 18-64 | 1665366 (95.1%) | | 514525 (59.6%) | | 1203275 (68.1%) | |  | |  | |
|  | 65-79 | 52470 (3.0%) | | 324802 (37.6%) | | 379775 (21.5%) | |  | |  | |
|  | 80+ | 32474 (1.9%) | | 23574 (2.7%) | | 184297 (10.4%) | |  | |  | |
| Deprivation status † | 1 - High | 378104 (21.6%) | | 158081 (18.3%) | | 320872 (18.2%) | |  | |  | |
|  | 2 | 347560 (19.9%) | | 170138 (19.7%) | | 344723 (19.5%) | |  | |  | |
|  | 3 | 329048 (18.8%) | | 171937 (19.9%) | | 366204 (20.7%) | |  | |  | |
|  | 4 | 324051 (18.5%) | | 184204 (21.3%) | | 367064 (20.8%) | |  | |  | |
|  | 5-Low | 347067 (19.8%) | | 174089 (20.2%) | | 359778 (20.4%) | |  | |  | |
|  | Unknown | 24480 (1.4%) | | 4452 (0.5%) | | 8706 (0.5%) | |  | |  | |
| Urban/Rural index | 1 Large Urban Areas | 761350 (43.5%) | | 260620 (30.2%) | | 535334 (30.3%) | |  | |  | |
|  | 2 Other Urban Areas | 554621 (31.7%) | | 344947 (40.0%) | | 661485 (37.4%) | |  | |  | |
|  | 3 Accessible Small Towns | 139019 (7.9%) | | 84252 (9.8%) | | 180264 (10.2%) | |  | |  | |
|  | 4 Remote Small Towns | 67946 (3.9%) | | 43206 (5.0%) | | 99311 (5.6%) | |  | |  | |
|  | 5 Accessible Rural | 135931 (7.8%) | | 75302 (8.7%) | | 182029 (10.3%) | |  | |  | |
|  | 6 Remote Rural | 66964 (3.8%) | | 50122 (5.8%) | | 100218 (5.7%) | |  | |  | |
|  | Unknown | 24480 (1.4%) | | 4452 (0.5%) | | 8706 (0.5%) | |  | |  | |
| Number of risk groups ‡ | 0 | 1324630 (75.7%) | | 448039 (51.9%) | | 829753 (46.9%) | |  | |  | |
|  | 1 | 343193 (19.6%) | | 251725 (29.2%) | | 526054 (29.8%) | |  | |  | |
|  | 2 | 62186 (3.6%) | | 101524 (11.8%) | | 239430 (13.5%) | |  | |  | |
|  | 3 | 12733 (0.7%) | | 38518 (4.5%) | | 100966 (5.7%) | |  | |  | |
|  | 4 | 4420 (0.3%) | | 14699 (1.7%) | | 43167 (2.4%) | |  | |  | |
|  | 5+ | 3147 (0.2%) | | 8396 (1.0%) | | 27977 (1.6%) | |  | |  | |
| Number of previous tests § | 0 | 1467860 (83.9%) | | 643067 (74.5%) | | 1477853 (83.6%) | |  | |  | |
|  | 1 | 214044 (12.2%) | | 116474 (13.5%) | | 202719 (11.5%) | |  | |  | |
|  | 2 | 40341 (2.3%) | | 31064 (3.6%) | | 46050 (2.6%) | |  | |  | |
|  | 3 | 9947 (0.6%) | | 11717 (1.4%) | | 15097 (0.9%) | |  | |  | |
|  | 4-9 | 10719 (0.6%) | | 24158 (2.8%) | | 18650 (1.1%) | |  | |  | |
|  | 10+ | 7399 (0.4%) | | 36421 (4.2%) | | 6978 (0.4%) | |  | |  | |
| Average household age ¶ | Mean (SD) | 34.5 (13.9) | | 52 (18.2) | | 54.6 (17.8) | |  | |  | |
|  | Median (IQR) | 31.7 (16.25) | | 54.5 (31.5) | | 55.3 (27.25) | |  | |  | |
| Number of people in household ¶ | 1 | 542866 (31.0%) | | 272231 (31.5%) | | 620387 (35.1%) | |  | |  | |
|  | 2 | 385511 (22.0%) | | 309859 (35.9%) | | 608712 (34.4%) | |  | |  | |
|  | 3-5 | 727433 (41.6%) | | 262802 (30.5%) | | 505624 (28.6%) | |  | |  | |
|  | 6-10 | 81156 (4.6%) | | 16887 (2.0%) | | 30930 (1.8%) | |  | |  | |
|  | 11-30 | 6537 (0.4%) | | 850 (0.1%) | | 1301 (0.1%) | |  | |  | |
|  | 31-100 | 1655 (0.1%) | | 217 (0.0%) | | 222 (0.0%) | |  | |  | |
|  | 101+ | 5152 (0.3%) | | 55 (0.0%) | | 171 (0.0%) | |  | |  | |
| BMI | Underweight | 22839 (1.3%) | | 7172 (0.8%) | | 17856 (1.0%) | |  | |  | |
|  | Normal weight | 221423 (12.7%) | | 108021 (12.5%) | | 216994 (12.3%) | |  | |  | |
|  | Overweight | 1361930 (77.8%) | | 550521 (63.8%) | | 1126894 (63.8%) | |  | |  | |
|  | Obese | 144119 (8.2%) | | 197187 (22.9%) | | 405603 (22.9%) | |  | |  | |
| Smoking status | Ex-Smoker | 135461 (7.7%) | | 140896 (16.3%) | | 299894 (17.0%) | |  | |  | |
|  | Non-Smoker | 659177 (37.7%) | | 343925 (39.9%) | | 679009 (38.4%) | |  | |  | |
|  | Smoker | 283557 (16.2%) | | 200773 (23.3%) | | 436263 (24.7%) | |  | |  | |
|  | Unknown | 672115 (38.4%) | | 177307 (20.5%) | | 352181 (19.9%) | |  | |  | |
| Atrial fibrillation | | | 6268 (0.4%) | | 26750 (3.1%) | | 72957 (4.1%) | |  | |  |
| Asthma | | | 206703 (11.8%) | | 114308 (13.2%) | | 247126 (14.0%) | |  | |  |
| Blood cancer | | | 1811 (0.1%) | | 5358 (0.6%) | | 14739 (0.8%) | |  | |  |
| Heart failure | | | 3408 (0.2%) | | 11265 (1.3%) | | 33232 (1.9%) | |  | |  |
| Cerebral palsy | | | 622 (0.0%) | | 1036 (0.1%) | | 4356 (0.2%) | |  | |  |
| Coronary heart disease | | | 11998 (0.7%) | | 57178 (6.6%) | | 133101 (7.5%) | |  | |  |
| Cirrhosis | | | 3279 (0.2%) | | 6106 (0.7%) | | 14366 (0.8%) | |  | |  |
| Congenital heart disease | | | 3598 (0.2%) | | 10123 (1.2%) | | 24413 (1.4%) | |  | |  |
| COPD | | | 9734 (0.6%) | | 33608 (3.9%) | | 90563 (5.1%) | |  | |  |
| Dementia | | | 2350 (0.1%) | | 7411 (0.9%) | | 17478 (1.0%) | |  | |  |
| Diabetes type 1 | | | 2046 (0.1%) | | 5554 (0.6%) | | 14921 (0.8%) | |  | |  |
| Diabetes type 2 | | | 16798 (1.0%) | | 76948 (8.9%) | | 167878 (9.5%) | |  | |  |
| Epilepsy | | | 8910 (0.5%) | | 12308 (1.4%) | | 43449 (2.5%) | |  | |  |
| Fracture | | | 51152 (2.9%) | | 40527 (4.7%) | | 97860 (5.5%) | |  | |  |
| Neurological disorder | | | 1386 (0.1%) | | 4300 (0.5%) | | 12602 (0.7%) | |  | |  |
| Parkinson’s | | | 576 (0.0%) | | 2403 (0.3%) | | 6063 (0.3%) | |  | |  |
| Pulmonary hypertension | | | 724 (0.0%) | | 1596 (0.2%) | | 6510 (0.4%) | |  | |  |
| Pulmonary rare | | | 1436 (0.1%) | | 5635 (0.7%) | | 16043 (0.9%) | |  | |  |
| Peripheral vascular disease | | | 3373 (0.2%) | | 11786 (1.4%) | | 28862 (1.6%) | |  | |  |
| Rheumatoid arthritis or SLE | | | 3455 (0.2%) | | 12601 (1.5%) | | 31283 (1.8%) | |  | |  |
| Respiratory cancer | | | 1192 (0.1%) | | 2461 (0.3%) | | 7260 (0.4%) | |  | |  |
| Severe mental illness | | | 150214 (8.6%) | | 121762 (14.1%) | | 267033 (15.1%) | |  | |  |
| Sickle cell disease | | | 391 (0.0%) | | 763 (0.1%) | | 1904 (0.1%) | |  | |  |
| Stroke/TIA | | | 7729 (0.4%) | | 31281 (3.6%) | | 79952 (4.5%) | |  | |  |
| Thrombosis or pulmonary embolus | | | 7879 (0.5%) | | 17365 (2.0%) | | 50876 (2.9%) | |  | |  |
| Care housing category | Care home | 996 (0.1%) | | 4547 (0.5%) | | 2474 (0.1%) | |  | |  | |
|  | Homeless | 2221 (0.1%) | | 355 (0.0%) | | 1424 (0.1%) | |  | |  | |
| Learning disability or Down's | Learning disability | 22384 (1.3%) | | 9518 (1.1%) | | 34498 (2.0%) | |  | |  | |
|  | Down's | 113 (0.0%) | | 360 (0.0%) | | 1198 (0.1%) | |  | |  | |
| Kidney disease | CKD5 without dialysis or transplant | 7523 (0.4%) | | 37526 (4.3%) | | 103489 (5.9%) | |  | |  | |
|  | CKD5 with dialysis | 537 (0.0%) | | 1204 (0.1%) | | 4331 (0.2%) | |  | |  | |
|  | CKD with transplant | 383 (0.0%) | | 1211 (0.1%) | | 3103 (0.2%) | |  | |  | |
| * COVID-19 hospitalisation or death for vaccinated population after date of 1^st^ dose vaccination  † Deprivation status: Scottish Index of Multiple Deprivation (SIMD) 2020  ‡ Number of risk groups: Individual QCOVID risk groups found in Extended Table 11  § Number of previous tests: Proxy for working in a high-risk occupation (e.g. healthcare worker)  ¶ Household information taken from September 2020  + Care homes: All types of QCOVID care homes | | | | | | | | | | | |

5.6.2 Descriptive table on matched cohort

Count of how many vaccine recipients were successfully matched, for first dose and second dose analyses.

Code: 03c_matching_ps_summary.R (lines 132-158) and

08c_matching_ps_summary.R (lines 347-393)

**First dose:**

| First dose vaccinated | Matched |
| --- | --- |
| 3,481,808 | 1,650,185 (47.4%) |

**Second dose:**

| Second dose vaccinated | Matched |
| --- | --- |
| 2,582,105 | 1,358,286 (52.6%) |

Population characteristics for vaccine controls and vaccine recipients by vaccine type in matched cohort analysis (excluding care home residents)

Code: 03c_matching_ps_summary.R (lines 102-169)

| **Characteristic** | **Level** | **Both vaccines** | | **BNT162b2** | | **ChAdOx1** | |
| --- | --- | --- | --- | --- | --- | --- | --- |
|  |  | **Unvaccinated** | **Vaccinated** | **Unvaccinated** | **Vaccinated** | **Unvaccinated** | **Vaccinated** |
| Total | | 2573874 | 2573874 | 833719 | 833719 | 1740155 | 1740155 |
| COVID-19 hospitalisation or death | All events, N (Rate per 1000*) | 2173 (8.9) | 1005 (4.1) | 840 (8.3) | 369 (3.7) | 1333 (9.2) | 636 (4.4) |
|  | Events 14 days+, N (Rate per 1000*) | 1029 (4.4) | 541 (2.3) | 488 (5) | 238 (2.4) | 541 (4) | 303 (2.2 |
| Sex | Female | 1418102 (55.1%) | 1403332 (54.5%) | 507290 (60.8%) | 508427 (61.0%) | 910812 (52.3%) | 894905 (51.4%) |
|  | Male | 1155772 (44.9%) | 1170542 (45.5%) | 326429 (39.2%) | 325292 (39.0%) | 829343 (47.7%) | 845250 (48.6%) |
| Age (years) | Mean (SD) | 57.9 (15.7) | 57.9 (15.7) | 56.1 (16.4) | 56.1 (16.4) | 58.8 (15.2) | 58.8 (15.2) |
|  | Median (IQR) | 58 (20) | 58 (20)` | 60 (23) | 60 (23) | 58 (19) | 58 (19) |
| Age group (years) | 18-64 | 1680152 (65.3%) | 1680152 (65.3%) | 491221 (58.9%) | 491221 (58.9%) | 1188931 (68.3%) | 1188931 (68.3%) |
|  | 65-79 | 691534 (26.9%) | 691534 (26.9%) | 319944 (38.4%) | 319944 (38.4%) | 371590 (21.4%) | 371590 (21.4%) |
|  | 80+ | 202188 (7.9%) | 202188 (7.9%) | 22554 (2.7%) | 22554 (2.7%) | 179634 (10.3%) | 179634 (10.3%) |
| Deprivation status † | 1 - High | 478638 (18.6%) | 468522 (18.2%) | 159802 (19.2%) | 152724 (18.3%) | 318836 (18.3%) | 315798 (18.1%) |
|  | 2 | 507601 (19.7%) | 503069 (19.5%) | 164176 (19.7%) | 163837 (19.7%) | 343425 (19.7%) | 339232 (19.5%) |
|  | 3 | 529350 (20.6%) | 525676 (20.4%) | 170884 (20.5%) | 165730 (19.9%) | 358466 (20.6%) | 359946 (20.7%) |
|  | 4 | 537544 (20.9%) | 539520 (21.0%) | 174078 (20.9%) | 178153 (21.4%) | 363466 (20.9%) | 361367 (20.8%) |
|  | 5 - Low | 508818 (19.8%) | 525164 (20.4%) | 160895 (19.3%) | 169391 (20.3%) | 347923 (20.0%) | 355773 (20.4%) |
|  | Unknown | 11923 (0.5%) | 11923 (0.5%) | 3884 (0.5%) | 3884 (0.5%) | 8039 (0.5%) | 8039 (0.5%) |
| Urban/Rural index | 1 Large Urban Areas | 783640 (30.4%) | 784728 (30.5%) | 258154 (31.0%) | 254808 (30.6%) | 525486 (30.2%) | 529920 (30.5%) |
|  | 2 Other Urban Areas | 971117 (37.7%) | 983172 (38.2%) | 318175 (38.2%) | 332206 (39.8%) | 652942 (37.5%) | 650966 (37.4%) |
|  | 3 Accessible Small Towns | 261494 (10.2%) | 259163 (10.1%) | 83271 (10.0%) | 81497 (9.8%) | 178223 (10.2%) | 177666 (10.2%) |
|  | 4 Remote Small Towns | 139935 (5.4%) | 137972 (5.4%) | 44273 (5.3%) | 40936 (4.9%) | 95662 (5.5%) | 97036 (5.6%) |
|  | 5 Accessible Rural | 259551 (10.1%) | 251953 (9.8%) | 76983 (9.2%) | 72623 (8.7%) | 182568 (10.5%) | 179330 (10.3%) |
|  | 6 Remote Rural | 146214 (5.7%) | 144963 (5.6%) | 48979 (5.9%) | 47765 (5.7%) | 97235 (5.6%) | 97198 (5.6%) |
|  | Unknown | 11923 (0.5%) | 11923 (0.5%) | 3884 (0.5%) | 3884 (0.5%) | 8039 (0.5%) | 8039 (0.5%) |
| Number of risk groups ‡ | 0 | 1243982 (48.3%) | 1255925 (48.8%) | 415784 (49.9%) | 432880 (51.9%) | 828198 (47.6%) | 823045 (47.3%) |
|  | 1 | 772262 (30.0%) | 763706 (29.7%) | 245304 (29.4%) | 243424 (29.2%) | 526958 (30.3%) | 520282 (29.9%) |
|  | 2 | 333638 (13.0%) | 330259 (12.8%) | 103715 (12.4%) | 98187 (11.8%) | 229923 (13.2%) | 232072 (13.3%) |
|  | 3 | 132252 (5.1%) | 133286 (5.2%) | 41209 (4.9%) | 37026 (4.4%) | 91043 (5.2%) | 96260 (5.5%) |
|  | 4 | 56040 (2.2%) | 55705 (2.2%) | 17117 (2.1%) | 14192 (1.7%) | 38923 (2.2%) | 41513 (2.4%) |
|  | 5+ | 35700 (1.4%) | 34993 (1.4%) | 10590 (1.3%) | 8010 (1.0%) | 25110 (1.4%) | 26983 (1.6%) |
| Number of previous tests § | 0 | 2105945 (81.8%) | 2100224 (81.6%) | 645635 (77.4%) | 637754 (76.5%) | 1460310 (83.9%) | 1462470 (84.0%) |
|  | 1 | 305290 (11.9%) | 311266 (12.1%) | 110764 (13.3%) | 114364 (13.7%) | 194526 (11.2%) | 196902 (11.3%) |
|  | 2 | 73485 (2.9%) | 73595 (2.9%) | 29242 (3.5%) | 29801 (3.6%) | 44243 (2.5%) | 43794 (2.5%) |
|  | 3 | 24995 (1.0%) | 24719 (1.0%) | 10565 (1.3%) | 10728 (1.3%) | 14430 (0.8%) | 13991 (0.8%) |
|  | 4-9 | 36566 (1.4%) | 36003 (1.4%) | 18077 (2.2%) | 18975 (2.3%) | 18489 (1.1%) | 17028 (1.0%) |
|  | 10+ | 27593 (1.1%) | 28067 (1.1%) | 19436 (2.3%) | 22097 (2.7%) | 8157 (0.5%) | 5970 (0.3%) |
| Smoking status | Ex-smoker | 427097 (16.6%) | 430664 (16.7%) | 135623 (16.3%) | 136623 (16.4%) | 291474 (16.7%) | 294041 (16.9%) |
|  | Non-smoker | 994635 (38.6%) | 1000968 (38.9%) | 327433 (39.3%) | 332476 (39.9%) | 667202 (38.3%) | 668492 (38.4%) |
|  | Smoker | 632266 (24.6%) | 622361 (24.2%) | 205181 (24.6%) | 193166 (23.2%) | 427085 (24.5%) | 429195 (24.7%) |
|  | Unknown | 519876 (20.2%) | 519881 (20.2%) | 165482 (19.8%) | 171454 (20.6%) | 354394 (20.4%) | 348427 (20.0%) |
| BMI | Underweight | 25608 (1.0%) | 24269 (0.9%) | 8309 (1.0%) | 6869 (0.8%) | 17299 (1.0%) | 17400 (1.0%) |
|  | Normal weight | 317547 (12.3%) | 317567 (12.3%) | 107387 (12.9%) | 103954 (12.5%) | 210160 (12.1%) | 213613 (12.3%) |
|  | Overweight | 1653585 (64.2%) | 1649494 (64.1%) | 528838 (63.4%) | 533473 (64.0%) | 1124747 (64.6%) | 1116021 (64.1%) |
|  | Obese | 577134 (22.4%) | 582544 (22.6%) | 189185 (22.7%) | 189423 (22.7%) | 387949 (22.3%) | 393121 (22.6%) |
| Average household age ¶ | Mean (SD) | 53.8 (17.8) | 53.8 (18.0) | 52.4 (18.0) | 52.2 (18.2) | 54.5 (17.7) | 54.5 (17.8) |
|  | Median (IQR) | 55 (27.8) | 55 (28.5) | 55 (31) | 55 (31.6) | 55 (26.5) | 55 (27.2) |
| Number of people in household ¶ | 1 | 889811 (34.6%) | 876778 (34.1%) | 274915 (33.0%) | 265299 (31.8%) | 614896 (35.3%) | 611479 (35.1%) |
|  | 2 | 885516 (34.4%) | 899194 (34.9%) | 293331 (35.2%) | 300388 (36.0%) | 592185 (34.0%) | 598806 (34.4%) |
|  | 3-5 | 749050 (29.1%) | 749389 (29.1%) | 248351 (29.8%) | 251047 (30.1%) | 500699 (28.8%) | 498342 (28.6%) |
|  | 6-10 | 47116 (1.8%) | 46095 (1.8%) | 16063 (1.9%) | 15969 (1.9%) | 31053 (1.8%) | 30126 (1.7%) |
|  | 11-30 | 1763 (0.1%) | 1889 (0.1%) | 787 (0.1%) | 785 (0.1%) | 976 (0.1%) | 1104 (0.1%) |
|  | 31-100 | 387 (0.0%) | 379 (0.0%) | 164 (0.0%) | 193 (0.0%) | 223 (0.0%) | 186 (0.0%) |
|  | 101+ | 231 (0.0%) | 150 (0.0%) | 108 (0.0%) | 38 (0.0%) | 123 (0.0%) | 112 (0.0%) |
| * Rate calculated using person years as denominator  † Deprivation status: Scottish Index of Multiple Deprivation (SIMD) 2020  ‡ Number of risk groups: Individual QCovid risk groups found in Extended Table 11  § Number of previous tests: Proxy for working in a high-risk occupation (e.g. healthcare worker)  ¶ Household information taken from September 2020 | | | | | | | |

### 5.6.3 Covariate balance plot

Standardised mean differences of population characteristics before and after matching for each vaccine

Code: 03c_matching_ps_summary (lines 342-470)


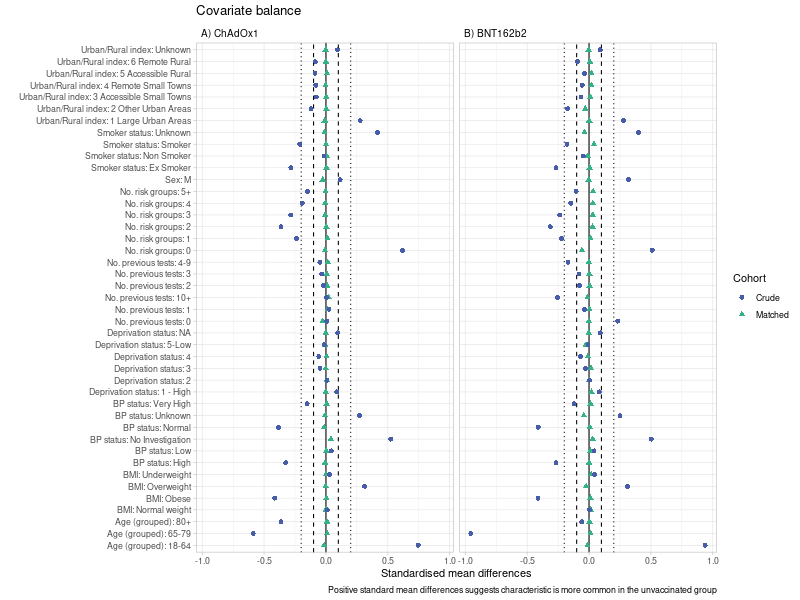


### 5.6.4 Table of risk outcomes stratified by follow up time period

Code: 07_meta_analysis (lines 62-114)

| **Vaccine** | **Period (days)** | **Age group** | **Unvaccinated Control** | | **First dose recipient** | | | **First dose vaccinated control** | | | **Second dose recipient** | |
| --- | --- | --- | --- | --- | --- | --- | --- | --- | --- | --- | --- | --- |
|  |  |  | **Person-days** | **Number of events** | **Person-days** | **Number of events** | **Person-days** | | **Number of events** | **Person-days** | | **Number of events** |
| **COVID-19 hospitalisations or deaths** | | | | | | | | | | | | |
| AZ | 0 | 18-64 |  |  |  |  |  | |  |  | |  |
| AZ | 1 | 18-64 |  |  |  |  |  | |  |  | |  |
| … | … | 18-64 |  |  |  |  |  | |  |  | |  |
| PB | 0 | 18-64 |  |  |  |  |  | |  |  | |  |
| PB | 1 | 18-64 |  |  |  |  |  | |  |  | |  |
| … | … | 18-64 |  |  |  |  |  | |  |  | |  |
| AZ | 0 | 65-79 |  |  |  |  |  | |  |  | |  |
| AZ | 1 | 65-79 |  |  |  |  |  | |  |  | |  |
| ... | ... | ... |  |  |  |  |  | |  |  | |  |
| PB | 0 | 65-79 |  |  |  |  |  | |  |  | |  |
| PB | 1 | 65-79 |  |  |  |  |  | |  |  | |  |
| ... | ... | ... |  |  |  |  |  | |  |  | |  |
| AZ | 0 | 80+ |  |  |  |  |  | |  |  | |  |
| AZ | 1 | 80+ |  |  |  |  |  | |  |  | |  |
| ... | ... | ... |  |  |  |  |  | |  |  | |  |
| PB | 0 | 80+ |  |  |  |  |  | |  |  | |  |
| PB | 1 | 80+ |  |  |  |  |  | |  |  | |  |
| ... | ... | ... |  |  |  |  |  | |  |  | |  |

### 5.6.5 Plot of GAMs

Rate ratios for the outcomes following a single vaccination dose over time in the matched cohort

Code: 03d_matching_modelling (lines 637-798)


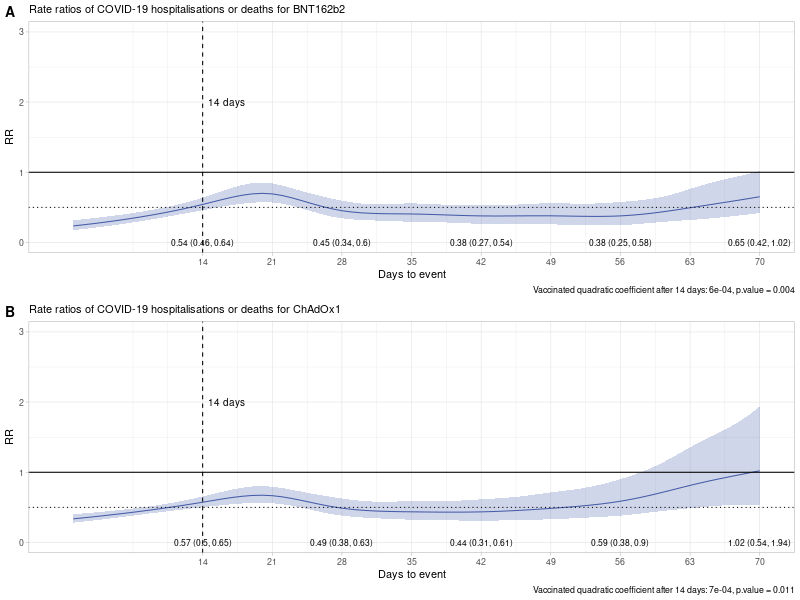


## 5.7 Missing data

Missing data will be reported as percentages of total or raw numbers where possible. Previous analyses have demonstrated that little missing data exist for our key variables of interest. For covariates which may have a higher proportion of missing data (such as body mass index), we will either use records with no item missingness or use a missing category.

## 5.8 Statistical software

All analyses will be carried out using R/RStudio, version 3.6.1.

# 6 Reporting results

## 6.1 Reporting guidelines and conventions

Results will be reported according to the Strengthening the Reporting of Observational Studies in Epidemiology (STROBE) and REporting of studies Conducted using Observational Routinely-collected Data (RECORD) (via the COVID-19 extension) guidelines. P-values will be quoted to two decimal places, unless they are less than 0.001 (whereby the p-value will be given as <0.001) or between <0.005 and >0.001, in which case they will be stated to three decimal places. Measures of association will be reported with 95% confidence intervals.

## 6.2 Dissemination

The analysis will be written in a manuscript and submitted to a peer reviewed journal. We will also seek to provide near real-time reports on vaccine safety, effectiveness and uptake for the various vaccines to the funders and government COVID-19 advisory bodies as appropriate.

All code will be made publicly available via a GitHub repository. R code for the Scotland’s vaccine waning analysis is found on the EAVE II GitHub page: <https://github.com/EAVE-II/Covid-vaccine-waning-pooled>. Within the code folder, scripts are available for reproducibility.

# References

[1] Danaei G, García Rodríguez LA, Cantero OF, Logan RW, Hernán MA. Electronic medical records can be used to emulate target trials of sustained treatment strategies. J Clin Epidemiol. 2018;96:12-22.

[2] Hernán MA, Robins JM. Using Big Data to Emulate a Target Trial When a Randomized Trial Is Not Available. Am J Epidemiol. 2016;183(8):758-64.

[3] Watson D, Spaulding AB, Dreyfus J. Risk-Set Matching to Assess the Impact of Hospital-Acquired Bloodstream Infections. Am J Epidemiol. 2018;188(2):461-6.

[4] Effectiveness of First Dose of COVID-19 Vaccines Against Hospital Admissions in Scotland: National Prospective Cohort Study of 5.4 Million People <https://papers.ssrn.com/sol3/papers.cfm?abstract_id=3789264>

[5] US Food and Drug Administration. Development and Licensure of Vaccines to Prevent COVID-19. 2020.

[6] Hodgson SH, Mansatta K, Mallett G, Harris V, Emary KRW, Pollard AJ. What defines an efficacious COVID-19 vaccine? A review of the challenges assessing the clinical efficacy of vaccines against SARS-CoV-2. The Lancet Infectious Diseases. 2021;21(2):e26-e35.

[7] Simpson, G. Comparing smooths in factor-smooth interactions. https://fromthebottomoftheheap.net/2017/10/10/difference-splines-i/ (10/05/2021)

[8] US Food and Drug Administration. Development and Licensure of Vaccines to Prevent COVID-19. (2020).

[9] Higgins JP, Thompson SG, Deeks JJ, Altman DG. Measuring inconsistency in meta-analyses. BMJ. 2003;327(7414):557-560.

# Appendix

### Table S1. ICD-10 codes

| **Code** | **Description** | **Category** |
| --- | --- | --- |
| U07.1 | COVID-19, virus identified | U07.1 |
| U07.2 | COVID-19, virus not identified | U07.2 |
| Source:<https://www.who.int/classifications/icd/COVID-19-coding-icd10.pdf> | | |
