# Supplementary material for: Waning of first- and second-dose ChAdOx1 and BNT162b2 COVID-19 vaccinations: a pooled target trial study of 12.9 million individuals in England, Northern Ireland, Scotland and Wales
Source: Int J Epidemiol. 2022 Oct 22;52(1):22–31. doi: 10.1093/ije/dyac199 (PMC9620314; doi:10.1093/ije/dyac199)
Supplement: dyac199_Supplementary_Data [file dyac199_supplementary_data.zip › dyac199_Supplementary_Data/ije-2022-04-0492-File010.docx]

**S3 Cohort summary tables**

**Table S3a: Cohort summary England**

| **Characteristic** | **Level** | **Unvaccinated** | **One dose ChAdOx1** | **Two doses  ChAdOx1** | **One dose BNT162b2** | **Two doses** **BNT162b2** |
| --- | --- | --- | --- | --- | --- | --- |
| Total | | 1,341,456 | 415,218 | 1,839,836 | 640,564 | 1,012,165 |
| Sex | Female | 661,237 (49.3%) | 207,904 (50.1%) | 970,634 (52.8%) | 341,806 (53.4%) | 595,500 (58.8%) |
|  | Male | 680,219 (50.7%) | 207,314 (49.9%) | 869,202 (47.2%) | 298,758 (46.6%) | 416,665 (41.2%) |
| Age (years) | Mean (SD) | 38.3 (15.2) | 51.2 (13.6) | 57.2 (14.6) | 35.4 (14) | 62.6 (18.7) |
|  | Median (IQR) | 35 (19) | 49 (17) | 57 (19) | 33 (12) | 65 (29) |
| Age group (years) | 18-64 | 1,245,362 (92.8%) | 344,249 (82.9%) | 1,257,858 (68.4%) | 599,644 (93.6%) | 498,792 (49.3%) |
|  | 65-79 | 69,161 (5.2%) | 59,192 (14.3%) | 497,741 (27.1%) | 270,00 (4.2%) | 291,300 (28.8%) |
|  | 80+ | 26,933 (2.0%) | 11,777 (2.8%) | 84,237 (4.6%) | 13,920 (2.2%) | 222,073 (21.9%) |
| Deprivation status † | 1 - High | 324,020 (24.2%) | 84,215 (20.3%) | 329,878 (17.9%) | 118,538 (18.5%) | 174,987 (17.3%) |
|  | 2 | 329,694 (24.6%) | 91,534 (22.0%) | 388,478 (21.1%) | 145,671 (22.7%) | 223,439 (22.1%) |
|  | 3 | 287,631 (21.4%) | 89,268 (21.5%) | 395,212 (21.5%) | 138,431 (21.6%) | 215,722 (21.3%) |
|  | 4 | 215,808 (16.1%) | 65,503 (15.8%) | 392,745 (21.3%) | 129,079 (20.2%) | 211,485 (20.9%) |
|  | 5-Low | 184,303 (13.7%) | 84,698 (20.4%) | 333,523 (18.1%) | 108,845 (17.0%) | 186,532 (18.4%) |
| Urban/Rural classification | Large Urban Areas | 557,912 (41.6%) | 118,786 (28.6%) | 497,638 (27.0%) | 212,187 (33.1%) | 289,650 (28.6%) |
|  | Other urban Areas | 36,230 (2.7%) | 9,564 (2.3%) | 72,236 (3.9%) | 21,065 (3.3%) | 36,053 (3.6%) |
|  | Rural town or village | 141,968 (10.6%) | 63,201 (15.2%) | 359,308 (19.5%) | 99,105 (15.5%) | 191,019 (18.9%) |
|  | Urban city or town | 605,346 (45.1%) | 223,667 (53.9%) | 910,654 (49.5%) | 308,207 (48.1%) | 495,443 (48.9%) |
| Number of risk groups ‡ | 0 | 925,499 (68.7%) | 239,641 (57.2%) | 942,341 (50.7%) | 430,304 (67.0%) | 399,386 (39.1%) |
|  | 1 | 309,731 (23.0%) | 117,096 (28.0%) | 553,381 (29.8%) | 162,564 (25.3%) | 321,608 (31.5%) |
|  | 2 | 77,387 (5.7%) | 38,971 (9.3%) | 218,733 (11.8%) | 35,424 (5.5%) | 166,553 (16.3%) |
|  | 3 | 20,207 (1.5%) | 13,488 (3.2%) | 84,131 (4.5%) | 8,152 (1.3%) | 75,556 (7.4%) |
|  | 4 | 7,767 (0.6%) | 5,348 (1.3%) | 35,176 (1.9%) | 3,135 (0.5%) | 33,996 (3.3%) |
|  | 5+ | 6,043 (0.4%) | 4,209 (1.0%) | 24,057 (1.3%) | 2,396 (0.4%) | 24,243 (2.4%) |
| Number of previous tests | 0 | 951,779 (71.0%) | 270,702 (65.2%) | 1,220,902 (66.4%) | 360,286 (56.2%) | 693,021 (68.5%) |
|  | 1 | 193,925 (14.5%) | 74,835 (18.0%) | 316,148 (17.2%) | 137,201 (21.4%) | 155,221 (15.3%) |
|  | 2 | 103,825 (7.7%) | 36,158 (8.7%) | 147,219 (8.0%) | 74,803 (11.7%) | 66,247 (6.5%) |
|  | 3 | 44,564 (3.3%) | 15,407 (3.7%) | 62,810 (3.4%) | 34,545 (5.4%) | 28,421 (2.8%) |
|  | 4-9 | 37,060 (2.8%) | 13,617 (3.3%) | 58,870 (3.2%) | 29,016 (4.5%) | 30,425 (3.0%) |
|  | 10+ | 10,303 (0.8%) | 4,499 (1.1%) | 33,887 (1.8%) | 4,713 (0.7%) | 38,830 (3.8%) |
| Household age | Mean (SD) | 32.4 (17.2) | 40.1 (20.9) | 48 (21.2) | 30.3 (17.5) | 54.9 (23.9) |
|  | Median (IQR) | 30 (20) | 41 (34) | 53 (36) | 28 (16) | 60 (41) |
| Number of people in household | 1 | 347,044 (25.9%) | 99,345 (23.9%) | 465,751 (25.3%) | 138,441 (21.6%) | 301,692 (29.8%) |
|  | 2 | 267,982 (20.0%) | 104,117 (25.1%) | 603,516 (32.8%) | 139,275 (21.7%) | 370,301 (36.6%) |
|  | 3-5 | 560,363 (41.8%) | 183,840 (44.3%) | 687,359 (37.4%) | 311,570 (48.6%) | 304,115 (30.0%) |
|  | 6-10 | 130,645 (9.7%) | 25,084 (6.0%) | 75,609 (4.1%) | 43,215 (6.7%) | 33,078 (3.3%) |
|  | 11-30 | 16,630 (1.2%) | 2,158 (0.5%) | 5,757 (0.3%) | 3,543 (0.6%) | 2,029 (0.2%) |
|  | 31-100 | 3,616 (0.3%) | 400 (0.1%) | 924 (0.1%) | 527 (0.1%) | 461 (0.0%) |
|  | 101+ | 15,176 (1.1%) | 274 (0.1%) | 920 (0.1%) | 3,993 (0.6%) | 489 (0.0%) |
| BMI | Underweight | 116,790 (8.7%) | 9,718 (2.3%) | 35,270 (1.9%) | 54,876 (8.6%) | 21,415 (2.1%) |
|  | Normal weight | 636,097 (47.4%) | 152,092 (36.6%) | 606,029 (32.9%) | 306,018 (47.8%) | 341,447 (33.7%) |
|  | Overweight | 364,636 (27.2%) | 146,602 (35.3%) | 666,683 (36.2%) | 173,878 (27.1%) | 361,131 (35.7%) |
|  | Obese | 223,933 (16.7%) | 106,806 (25.7%) | 531,854 (28.9%) | 105,792 (16.5%) | 288,172 (28.5%) |
| Smoking status | Ex-smoker | 244,035 (18.2%) | 111,586 (26.9%) | 546,869 (29.7%) | 119,355 (18.6%) | 323,357 (31.9%) |
|  | Non-smoker | 726,786 (54.2%) | 224,123 (54.0%) | 1,021,854 (55.5%) | 394,936 (61.7%) | 573,818 (56.7%) |
|  | Smoker | 311,923 (23.3%) | 77,018 (18.5%) | 263,217 (14.3%) | 101,986 (15.9%) | 110,664 (10.9%) |
|  | Unknown | 58,712 (4.4%) | 2,491 (0.6%) | 7,896 (0.4%) | 24,287 (3.8%) | 4,326 (0.4%) |
| Atrial fibrillation | | 10,714 (0.8%) | 8,023 (1.9%) | 57,096 (3.1%) | 5,412 (0.8%) | 67,857 (6.7%) |
| Asthma | | 183,629 (13.7%) | 62,285 (15.0%) | 283,576 (15.4%) | 106,518 (16.6%) | 162,224 (16.0%) |
| Blood cancer | | 3,970 (0.3%) | 2,405 (0.6%) | 17,515 (1.0%) | 1,591 (0.2%) | 16,399 (1.6%) |
| Heart failure | | 7,693 (0.6%) | 5,045 (1.2%) | 33,242 (1.8%) | 3,271 (0.5%) | 35,419 (3.5%) |
| Cerebral palsy | | 728 (0.1%) | 469 (0.1%) | 2,839 (0.2%) | 216 (0.0%) | 1,212 (0.1%) |
| Coronary heart disease | | 16,260 (1.2%) | 11,799 (2.8%) | 86,021 (4.7%) | 7,359 (1.1%) | 90,420 (8.9%) |
| Cirrhosis | | 1,635 (0.1%) | 1,041 (0.3%) | 5,665 (0.3%) | 529 (0.1%) | 4,045 (0.4%) |
| Congenital heart disease | | 4,453 (0.3%) | 2,259 (0.5%) | 13,648 (0.7%) | 1,511 (0.2%) | 9,208 (0.9%) |
| COPD | | 11,848 (0.9%) | 8,632 (2.1%) | 59,688 (3.2%) | 4,816 (0.8%) | 52,478 (5.2%) |
| Dementia | | 5,183 (0.4%) | 3,150 (0.8%) | 15,527 (0.8%) | 1,759 (0.3%) | 16,663 (1.6%) |
| Diabetes type 1 | | 3,791 (0.3%) | 2,287 (0.6%) | 14,017 (0.8%) | 1,370 (0.2%) | 10,512 (1.0%) |
| Diabetes type 2 | | 33,303 (2.5%) | 23,394 (5.6%) | 158,952 (8.6%) | 13,596 (2.1%) | 138,866 (13.7%) |
| Epilepsy | | 23,044 (1.7%) | 8,870 (2.1%) | 43,884 (2.4%) | 10,542 (1.6%) | 23,287 (2.3%) |
| Fracture | | 34,867 (2.6%) | 13,851 (3.3%) | 72,003 (3.9%) | 20,765 (3.2%) | 52,006 (5.1%) |
| Neurological disorder | | 1,927 (0.1%) | 1,340 (0.3%) | 8,379 (0.5%) | 610 (0.1%) | 5,606 (0.6%) |
| Parkinson’s | | 1,257 (0.1%) | 986 (0.2%) | 6,365 (0.3%) | 460 (0.1%) | 5,679 (0.6%) |
| Pulmonary hypertension | | 1,687 (0.1%) | 1,087 (0.3%) | 6,783 (0.4%) | 794 (0.1%) | 7,202 (0.7%) |
| Pulmonary rare | | 2,571 (0.2%) | 1,896 (0.5%) | 13,993 (0.8%) | 1,203 (0.2%) | 13,347 (1.3%) |
| Peripheral vascular disease | | 3,641 (0.3%) | 2,579 (0.6%) | 17,107 (0.9%) | 1,513 (0.2%) | 16,685 (1.6%) |
| Rheumatoid arthritis or SLE | | 7,873 (0.6%) | 5,225 (1.3%) | 33,737 (1.8%) | 3,336 (0.5%) | 24,049 (2.4%) |
| Respiratory cancer | | 1,809 (0.1%) | 1,064 (0.3%) | 6,016 (0.3%) | 582 (0.1%) | 5,155 (0.5%) |
| Severe mental illness | | 159,301 (11.9%) | 73,891 (17.8%) | 313,651 (17.0%) | 70,775 (11.0%) | 166,956 (16.5%) |
| Sickle cell disease | | 801 (0.1%) | 251 (0.1%) | 1,510 (0.1%) | 155 (0.0%) | 1,101 (0.1%) |
| Stroke/TIA | | 11,070 (0.8%) | 7,341 (1.8%) | 52,404 (2.8%) | 4,387 (0.7%) | 54,141 (5.3%) |
| Thrombosis or pulmonary embolus | | 5,090 (0.4%) | 3,071 (0.7%) | 19,891 (1.1%) | 1,705 (0.3%) | 16,906 (1.7%) |
| Care housing category | Care home | 5,178 (0.0%) | 3,535 (0.8%) | 17,983 (1.0%) | 1,411 (0.2%) | 9,177 (0.9%) |
|  | Homeless | 5,097 (0.4%) | 1,201 (0.3%) | 2,404 (0.1%) | 756 (0.1%) | 815 (0.1%) |
| Learning disability or Down's | Learning disability | 15,505 (1.2%) | 5,866 (1.4%) | 32,640 (1.8%) | 6,580 (1.0%) | 16,441 (1.6%) |
|  | Down's | 262 (0.0%) | 202 (0.0%) | 1,615 (0.1%) | 80 (0.0%) | 630 (0.1%) |
| Kidney disease | CKD5 without dialysis or transplant | 17,059 (1.3%) | 11,460 (2.8%) | 87,758 (4.8%) | 7,775 (1.2%) | 110,150 (10.9%) |
|  | CKD5 with dialysis | 228 (0.0%) | 135 (0.0%) | 785 (0.0%) | 72 (0.0%) | 725 (0.1%) |
|  | CKD with transplant | 223 (0.0%) | 191 (0.0%) | 1,348 (0.1%) | 121 (0.0%) | 1,026 (0.1%) |
| Vaccination categories refer to vaccination status at the end of the study period - 30 June 2021. † Deprivation status: Quintiles of the English Index of Deprivation 2019  ‡ Number of risk groups: Count of QCovid risk groups: [doi:10.1136/bmj.m3731](https://www.bmj.com/content/371/bmj.m3731) | | | | | | |

**Table S3b: Cohort summary Northern Ireland**

| **Characteristic** | **Levels** | **Unvaccinated** | **One dose ChAdOx1** | **Two doses ChAdOx1** | **One dose BNT162b2** | **Two doses BNT162b2** |
| --- | --- | --- | --- | --- | --- | --- |
| Total | | 351,843 | 114,291 | 484,588 | 145,249 | 345,765 |
| Sex | Female | 169,356 (48.1%) | 50,578 (44.3%) | 243,687 (50.3%) | 69,263 (47.7%) | 203,367 (58.8%) |
|  | Male | 182,487 (51.9%) | 63,713 (55.7%) | 240,901 (49.7%) | 75,986 (52.3%) | 142,398 (41.2%) |
| Age | Mean (SD) | 37.1 (15.8) | 43.5 (14.9) | 60.5 (16.4) | 30.7 (9.5) | 51.4 (13.3) |
|  | Median (IQR) | 33 (20) | 40 (16) | 60 (25) | 29 (13) | 54 (20) |
| Age group | <65 | 326,845 (92.9%) | 102,722 (89.9%) | 284,683 (58.7%) | 144,379 (99.4%) | 277,141 (80.2%) |
|  | 65-79 | 16,806 (4.8%) | 7,630 (6.7%) | 138,417 (28.6%) | 844 (0.6%) | 68,523 (19.8%) |
|  | 80+ | 8,192 (2.3%) | 3,939 (3.4%) | 61,488 (12.7%) | 26 (0.0%) | 101 (0.0%) |
| Deprivation Status† | 1 - High | 47,767 (13.6%) | 13,314 (11.6%) | 39,356 (8.1%) | 11,667 (8.0%) | 24,350 (7.0%) |
|  | 2 | 42,967 (12.2%) | 13,360 (11.7%) | 45,917 (9.5%) | 12,802 (8.8%) | 27,020 (7.8%) |
|  | 3 | 40,095 (11.4%) | 12,326 (10.8%) | 47,125 (9.7%) | 13,309 (9.2%) | 29,703 (8.6%) |
|  | 4 | 40,133 (11.4%) | 12,725 (11.1%) | 50,188 (10.4%) | 14,962 (10.3%) | 34,225 (9.9%) |
|  | 5 | 39,152 (11.1%) | 12,462 (10.9%) | 51,524 (10.6%) | 15,069 (10.4%) | 35,630 (10.3%) |
|  | 6 | 32,626 (9.3%) | 10,574 (9.3%) | 48,725 (10.1%) | 14,874 (10.2%) | 34,335 (9.9%) |
|  | 7 | 34,096 (9.7%) | 10,165 (8.9%) | 49,409 (10.2%) | 17,070 (11.8%) | 39,709 (11.5%) |
|  | 8 | 29,729 (8.4%) | 10,081 (8.8%) | 50,374 (10.4%) | 16,494 (11.4%) | 41,581 (12.0%) |
|  | 9 | 24,261 (6.9%) | 9,951 (8.7%) | 48,709 (10.1%) | 15,050 (10.4%) | 39,341 (11.4%) |
|  | 10 - Low | 21,017 (6.0%) | 9,333 (8.2%) | 53,261 (11.0%) | 13,952 (9.6%) | 39,871 (11.5%) |
| Settlement band | A Belfast Metropolitan Urban Area | 61,651 (17.5%) | 19,694 (17.2%) | 72,364 (14.9%) | 19,910 (13.7%) | 46,396 (13.4%) |
|  | B Derry Urban Area | 17,804 (5.1%) | 6,859 (6.0%) | 21,742 (4.5%) | 6,956 (4.8%) | 16,406 (4.7%) |
|  | C Large town | 98,533 (28.0%) | 32,285 (28.2%) | 144,699 (29.9%) | 41,225 (28.4%) | 100,699 (29.1%) |
|  | D Medium town | 28,258 (8.0%) | 8,232 (7.2%) | 33,583 (6.9%) | 10,069 (6.9%) | 22,323 (6.5%) |
|  | E Small town | 20,914 (5.9%) | 7,154 (6.3%) | 31,809 (6.6%) | 9,228 (6.4%) | 22,491 (6.5%) |
|  | F Intermediate settlement | 13,287 (3.8%) | 4,349 (3.8%) | 20,990 (4.3%) | 6,400 (4.4%) | 15,754 (4.6%) |
|  | G Village | 19,615 (5.6%) | 6,789 (5.9%) | 26,489 (5.5%) | 8,219 (5.7%) | 19,020 (5.5%) |
|  | H Small village, hamlet and open countryside | 91,780 (26.1%) | 28,929 (25.3%) | 132,912 (27.4%) | 43,242 (29.8%) | 102,676 (29.7%) |
| BNF chapters prescribed‡ | 0 | 252,485 (71.8%) | 70,869 (62.0%) | 146,618 (30.3%) | 118,371 (81.5%) | 166,953 (48.3%) |
|  | 1 | 48,532 (13.8%) | 18,769 (16.4%) | 98,807 (20.4%) | 17,842 (12.3%) | 76,140 (22.0%) |
|  | 2 | 24,005 (6.8%) | 10,214 (8.9%) | 84,762 (17.5%) | 5,527 (3.8%) | 47,759 (13.8%) |
|  | 3 | 13,484 (3.8%) | 6,512 (5.7%) | 65,724 (13.6%) | 2,166 (1.5%) | 27,993 (8.1%) |
|  | 4 | 7,339 (2.1%) | 4,014 (3.5%) | 45,603 (9.4%) | 855 (0.6%) | 15,268 (4.4%) |
|  | 5 | 3,739 (1.1%) | 2,301 (2.0%) | 26,011 (5.4%) | 323 (0.2%) | 7,344 (2.1%) |
|  | 6+ | 2,259 (0.6%) | 1,612 (1.4%) | 17,063 (3.5%) | 165 (0.1%) | 4,308 (1.2%) |
| Number of previous tests | 0 | 265,303 (75.4%) | 85,047 (74.4%) | 382,665 (79.0%) | 101,075 (69.6%) | 245,693 (71.1%) |
|  | 1 | 58,419 (16.6%) | 21,069 (18.4%) | 72,878 (15.0%) | 31,099 (21.4%) | 62,248 (18.0%) |
|  | 2 | 15,816 (4.5%) | 5,374 (4.7%) | 18,522 (3.8%) | 8,509 (5.9%) | 17,521 (5.1%) |
|  | 3 | 4,866 (1.4%) | 1,525 (1.3%) | 5,596 (1.2%) | 2,288 (1.6%) | 5,666 (1.6%) |
|  | 4-9 | 5,800 (1.6%) | 1,150 (1.0%) | 4,602 (0.9%) | 1,815 (1.2%) | 9,773 (2.8%) |
|  | 10+ | 1,639 (0.5%) | 126 (0.1%) | 325 (0.1%) | 463 (0.3%) | 4,864 (1.4%) |
| Household age | Mean (SD) | 37.1 (14.3) | 40 (16.1) | 53.7 (18.9) | 34.8 (10.5) | 44.4 (14.5) |
|  | Median (IQR) | 34.9 (17.3) | 37 (22) | 53.5 (32.8) | 34.2 (14) | 43.3 (23) |
| Number of people in household | 1 | 33,927 (9.6%) | 13,853 (12.1%) | 84,402 (17.4%) | 8,487 (5.8%) | 33,255 (9.6%) |
|  | 2 | 55,533 (15.8%) | 20,996 (18.4%) | 144,009 (29.7%) | 18,035 (12.4%) | 78,484 (22.7%) |
|  | 3-5 | 193,674 (55.0%) | 64,209 (56.2%) | 214,380 (44.2%) | 95,770 (65.9%) | 196,769 (56.9%) |
|  | 6-10 | 54,338 (15.4%) | 11,969 (10.5%) | 30,952 (6.4%) | 19,013 (13.1%) | 29,383 (8.5%) |
|  | 11-30 | 3,324 (0.9%) | 478 (0.4%) | 978 (0.2%) | 590 (0.4%) | 881 (0.3%) |
|  | 31-100 | 360 (0.1%) | 32 (0.0%) | 69 (0.0%) | 36 (0.0%) | 57 (0.0%) |
|  | 101+ | 862 (0.2%) | 44 (0.0%) | 19 (0.0%) | 196 (0.1%) | 120 (0.0%) |
|  | Missing | 9,825 (2.8%) | 2,710 (2.4%) | 9,779 (2.0%) | 3,122 (2.1%) | 6,816 (2.0%) |
| Vaccination categories refer to vaccination status at the end of the study period - 30 June 2021.  † Deprivation Status: Deciles of the Northern Ireland Multiple Deprivation Measure 2017.  ‡ BNF chapters prescribed: Number of chapters of the British National Formulary (BNF) from which individuals received repeat prescriptions prior to vaccination. | | | | | | |

**Table S3c: Cohort summary Scotland**

| **Characteristic** | **Levels** | **Unvaccinated** | **One dose ChAdOx1** | **Two doses ChAdOx1** | **One dose BNT162b2** | **Two doses BNT162b2** |
| --- | --- | --- | --- | --- | --- | --- |
| Total |  | 822,875 (100.0%) | 317,418 (100.0%) | 1,700,070 (100.0%) | 579,718 (100.0%) | 864,482 (100.0%) |
| Sex | Female | 377,773 (45.9%) | 143,162 (45.1%) | 877,900 (51.6%) | 271,670 (46.9%) | 531,514 (61.5%) |
|  | Male | 445,102 (54.1%) | 174,256 (54.9%) | 822,170 (48.4%) | 308,048 (53.1%) | 332,968 (38.5%) |
| Age | Mean (SD) | 36.1 (16.4) | 45.7 (10.3) | 59.1 (15.2) | 31.3 (8.6) | 55.8 (16.3) |
|  | Median (IQR) | 31 (19) | 43 (7) | 58 (19) | 31 (11) | 59 (24) |
| Age group | 18-64 | 757,921 (92.1%) | 299,340 (94.3%) | 1,147,449 (67.5%) | 575,196 (99.2%) | 518,717 (60.0%) |
|  | 65-79 | 39,135 (4.8%) | 10,352 (3.3%) | 374,312 (22.0%) | 3,510 (0.6%) | 323,028 (37.4%) |
|  | 80+ | 25,820 (3.1%) | 7,726 (2.4%) | 178,309 (10.5%) | 1,012 (0.2%) | 22,737 (2.6%) |
| Deprivation status † | 1 - High | 205,432 (25.0%) | 66,669 (21.0%) | 298,544 (17.6%) | 108,919 (18.8%) | 157,005 (18.2%) |
|  | 2 | 169,211 (20.6%) | 63,951 (20.1%) | 329,494 (19.4%) | 113,974 (19.7%) | 169,312 (19.6%) |
|  | 3 | 152,275 (18.5%) | 60,508 (19.1%) | 354,759 (20.9%) | 113,319 (19.5%) | 172,125 (19.9%) |
|  | 4 | 133,425 (16.2%) | 61,465 (19.4%) | 357,544 (21.0%) | 122,379 (21.1%) | 185,171 (21.4%) |
|  | 5-Low | 146,503 (17.8%) | 62,566 (19.7%) | 351,497 (20.7%) | 116,611 (20.1%) | 176,467 (20.4%) |
|  | Missing | 16,029 (1.9%) | 2,259 (0.7%) | 8,232 (0.5%) | 4,516 (0.8%) | 4,402 (0.5%) |
| Urban/Rural classification | Large Urban Areas | 400,059 (48.6%) | 106,845 (33.7%) | 504,854 (29.7%) | 198,731 (34.3%) | 265,389 (30.7%) |
|  | Other Urban Areas | 228,786 (27.8%) | 127,283 (40.1%) | 639,270 (37.6%) | 219,520 (37.9%) | 341,614 (39.5%) |
|  | Accessible Small Towns | 59,193 (7.2%) | 29,661 (9.3%) | 175,173 (10.3%) | 52,415 (9.0%) | 84,055 (9.7%) |
|  | Remote Small Towns | 30,652 (3.7%) | 12,956 (4.1%) | 97,714 (5.7%) | 25,587 (4.4%) | 43,383 (5.0%) |
|  | Accessible Rural | 57,018 (6.9%) | 28,243 (8.9%) | 176,139 (10.4%) | 51,608 (8.9%) | 75,019 (8.7%) |
|  | Remote Rural | 31,137 (3.8%) | 10,171 (3.2%) | 98,688 (5.8%) | 27,341 (4.7%) | 50,620 (5.9%) |
|  | Unknown | 16,029 (1.9%) | 2,259 (0.7%) | 8,232 (0.5%) | 4,516 (0.8%) | 4,402 (0.5%) |
| Number of risk groups ‡ | 0 | 624,911 (75.9%) | 212,410 (66.9%) | 799,570 (47.0%) | 432,540 (74.6%) | 453,079 (52.4%) |
|  | 1 | 149,875 (18.2%) | 76,134 (24.0%) | 505,125 (29.7%) | 126,668 (21.8%) | 250,841 (29.0%) |
|  | 2 | 33,366 (4.1%) | 19,062 (6.0%) | 230,070 (13.5%) | 17,399 (3.0%) | 100,407 (11.6%) |
|  | 3 | 8,878 (1.1%) | 5,648 (1.8%) | 97,190 (5.7%) | 2,069 (0.4%) | 37,794 (4.4%) |
|  | 4 | 3,330 (0.4%) | 2,420 (0.8%) | 41,443 (2.4%) | 600 (0.1%) | 14,309 (1.7%) |
|  | 5+ | 2,515 (0.3%) | 1,744 (0.5%) | 26,672 (1.6%) | 442 (0.1%) | 8,052 (0.9%) |
| Number of previous tests | 0 | 698,800 (84.9%) | 261,076 (82.2%) | 1,418,251 (83.4%) | 463,390 (79.9%) | 643,020 (74.4%) |
|  | 1 | 87,888 (10.7%) | 42,333 (13.3%) | 197,928 (11.6%) | 90,477 (15.6%) | 118,433 (13.7%) |
|  | 2 | 18,373 (2.2%) | 8,149 (2.6%) | 44,872 (2.6%) | 16,930 (2.9%) | 31,388 (3.6%) |
|  | 3 | 5,123 (0.6%) | 2,117 (0.7%) | 14,538 (0.9%) | 3,810 (0.7%) | 11,093 (1.3%) |
|  | 4-9 | 7,087 (0.9%) | 2,497 (0.8%) | 17,441 (1.0%) | 3,014 (0.5%) | 22,047 (2.6%) |
|  | 10+ | 5,604 (0.7%) | 1,246 (0.4%) | 7,040 (0.4%) | 2,097 (0.4%) | 38,501 (4.5%) |
| Household age | Mean (SD) | 35.8 (15.8) | 38.6 (14.5) | 54.9 (17.8) | 31.3 (10.5) | 51.9 (18.2) |
|  | Median (IQR) | 32 (17.95) | 37.5 (18.75) | 56 (27) | 30 (13.67) | 54 (31.5) |
| Number of people in household | 1 | 301,184 (36.6%) | 86,963 (27.4%) | 594,660 (35.0%) | 139,155 (24.0%) | 271,417 (31.4%) |
|  | 2 | 180,398 (21.9%) | 69,284 (21.8%) | 590,885 (34.8%) | 133,185 (23.0%) | 309,554 (35.8%) |
|  | 3-5 | 287,407 (34.9%) | 148,709 (46.8%) | 484,254 (28.5%) | 283,716 (48.9%) | 265,521 (30.7%) |
|  | 6-10 | 43,915 (5.3%) | 11,747 (3.7%) | 28,867 (1.7%) | 22,475 (3.9%) | 16,913 (2.0%) |
|  | 11-30 | 4,565 (0.6%) | 458 (0.1%) | 1,193 (0.1%) | 851 (0.1%) | 842 (0.1%) |
|  | 31-100 | 1,288 (0.2%) | 111 (0.0%) | 140 (0.0%) | 132 (0.0%) | 209 (0.0%) |
|  | 101+ | 4,118 (0.5%) | 146 (0.0%) | 71 (0.0%) | 204 (0.0%) | 26 (0.0%) |
| BMI | Underweight | 13,619 (1.7%) | 2,835 (0.9%) | 16,520 (1.0%) | 7,086 (1.2%) | 6,942 (0.8%) |
|  | Normal weight | 103,257 (12.5%) | 34,307 (10.8%) | 208,072 (12.2%) | 83,318 (14.4%) | 107,575 (12.4%) |
|  | Overweight | 647,884 (78.7%) | 239,780 (75.5%) | 1,082,151 (63.7%) | 436,414 (75.3%) | 553,620 (64.0%) |
|  | Obese | 58,115 (7.1%) | 40,496 (12.8%) | 393,327 (23.1%) | 52,900 (9.1%) | 196,345 (22.7%) |
| Smoking status | Ex-smoker | 55,027 (6.7%) | 38,211 (12.0%) | 291,770 (17.2%) | 42,581 (7.3%) | 140,865 (16.3%) |
|  | Non-smoker | 279,551 (34.0%) | 113,784 (35.8%) | 659,033 (38.8%) | 248,687 (42.9%) | 346,487 (40.1%) |
|  | Smoker | 134,491 (16.3%) | 78,403 (24.7%) | 411,663 (24.2%) | 83,097 (14.3%) | 199,034 (23.0%) |
|  | Unknown | 353,806 (43.0%) | 87,020 (27.4%) | 337,604 (19.9%) | 205,353 (35.4%) | 178,096 (20.6%) |
| Atrial fibrillation | | 4,819 (0.6%) | 3,072 (1.0%) | 70,772 (4.2%) | 663 (0.1%) | 26,341 (3.0%) |
| Asthma | | 86,583 (10.5%) | 40,009 (12.6%) | 236,627 (13.9%) | 84,754 (14.6%) | 113,965 (13.2%) |
| Blood cancer | | 1,318 (0.2%) | 665 (0.2%) | 14,290 (0.8%) | 236 (0.0%) | 5,294 (0.6%) |
| Heart failure | | 2,708 (0.3%) | 1,780 (0.6%) | 31,891 (1.9%) | 385 (0.1%) | 10,984 (1.3%) |
| Cerebral palsy | | 412 (0.1%) | 218 (0.1%) | 4,233 (0.2%) | 111 (0.0%) | 1,006 (0.1%) |
| Coronary heart disease | | 9,055 (1.1%) | 5,677 (1.8%) | 129,110 (7.6%) | 1,293 (0.2%) | 56,402 (6.5%) |
| Cirrhosis | | 1,782 (0.2%) | 1,356 (0.4%) | 13,701 (0.8%) | 764 (0.1%) | 5,999 (0.7%) |
| Congenital heart disease | | 2,605 (0.3%) | 1,129 (0.4%) | 23,580 (1.4%) | 619 (0.1%) | 10,003 (1.2%) |
| COPD | | 7,319 (0.9%) | 5,118 (1.6%) | 86,943 (5.1%) | 1,077 (0.2%) | 32,902 (3.8%) |
| Dementia | | 1,856 (0.2%) | 1,106 (0.3%) | 16,684 (1.0%) | 521 (0.1%) | 6,999 (0.8%) |
| Diabetes type 1 | | 1,575 (0.2%) | 618 (0.2%) | 14,442 (0.8%) | 252 (0.0%) | 5,478 (0.6%) |
| Diabetes type 2 | | 12,266 (1.5%) | 7,062 (2.2%) | 163,342 (9.6%) | 1,839 (0.3%) | 76,023 (8.8%) |
| Epilepsy | | 6,412 (0.8%) | 3,706 (1.2%) | 40,788 (2.4%) | 1,228 (0.2%) | 12,067 (1.4%) |
| Fracture | | 23,275 (2.8%) | 11,853 (3.7%) | 93,789 (5.5%) | 18,872 (3.3%) | 40,141 (4.6%) |
| Neurological disorder | | 969 (0.1%) | 543 (0.2%) | 12,256 (0.7%) | 136 (0.0%) | 4,276 (0.5%) |
| Parkinson’s | | 429 (0.1%) | 254 (0.1%) | 5,904 (0.3%) | 80 (0.0%) | 2,350 (0.3%) |
| Pulmonary hypertension | | 583 (0.1%) | 391 (0.1%) | 6,212 (0.4%) | 60 (0.0%) | 1,558 (0.2%) |
| Pulmonary rare | | 1,040 (0.1%) | 663 (0.2%) | 15,607 (0.9%) | 154 (0.0%) | 5,577 (0.6%) |
| Peripheral vascular disease | | 2,463 (0.3%) | 1,590 (0.5%) | 27,776 (1.6%) | 479 (0.1%) | 11,530 (1.3%) |
| Rheumatoid arthritis or SLE | | 2,480 (0.3%) | 1,342 (0.4%) | 30,387 (1.8%) | 441 (0.1%) | 12,481 (1.4%) |
| Respiratory cancer | | 917 (0.1%) | 574 (0.2%) | 6,846 (0.4%) | 154 (0.0%) | 2,379 (0.3%) |
| Severe mental illness | | 67,529 (8.2%) | 45,219 (14.2%) | 252,621 (14.9%) | 46,339 (8.0%) | 120,431 (13.9%) |
| Sickle cell disease | | 256 (0.0%) | 124 (0.0%) | 1,839 (0.1%) | 67 (0.0%) | 755 (0.1%) |
| Stroke/TIA | | 5,806 (0.7%) | 3,730 (1.2%) | 77,364 (4.6%) | 906 (0.2%) | 30,713 (3.6%) |
| Thrombosis or pulmonary embolus | | 5,889 (0.7%) | 3,463 (1.1%) | 48,320 (2.8%) | 937 (0.2%) | 17,087 (2.0%) |
| Housing category | Care home | 711 (0.1%) | 239 (0.1%) | 2,346 (0.1%) | 392 (0.1%) | 4,286 (0.5%) |
|  | Homeless | 1,478 (0.2%) | 517 (0.2%) | 1,166 (0.1%) | 361 (0.1%) | 323 (0.0%) |
| Learning disability or Down’s | Learning disability | 11,747 (1.4%) | 3,974 (1.3%) | 32,522 (1.9%) | 8,485 (1.5%) | 9,325 (1.1%) |
|  | Down’s | 78 (0.0%) | 42 (0.0%) | 1,181 (0.1%) | 9 (0.0%) | 354 (0.0%) |
| Kidney disease | CKD 5 without dialysis or transplant | 5,693 (0.7%) | 4,209 (1.3%) | 100,487 (5.9%) | 886 (0.2%) | 36,924 (4.3%) |
|  | CKD 5 with dialysis | 418 (0.1%) | 268 (0.1%) | 4,145 (0.2%) | 58 (0.0%) | 1,167 (0.1%) |
|  | CKD 5 with transplant | 297 (0.0%) | 153 (0.0%) | 2,989 (0.2%) | 53 (0.0%) | 1,187 (0.1%) |
| Vaccination categories refer to vaccination status at the end of the study period - 30 June 2021. † Deprivation status: Quintiles of the Scottish Index of Multiple Deprivation (SIMD) 2020  ‡ Number of risk groups: Count of QCovid risk groups: [doi:10.1136/bmj.m3731](https://www.bmj.com/content/371/bmj.m3731) | | | | | | |

**Table S3d: Cohort summary Wales**

| **Characteristic** | **Levels** | **Unvaccinated** | **One dose ChAdOx1** | **Two doses ChAdOx1** | **One dose BNT162b2** | **Two doses BNT162b2** |
| --- | --- | --- | --- | --- | --- | --- |
| Total |  | 301264 | 34044 | 913278 | 99972 | 611720 |
| Sex | Female | 174800 (58.0) | 17229 (50.6) | 452512 (49.5) | 55006 (55.0) | 273350 (44.7) |
|  | Male | 126464 (42.0) | 16815 (49.4) | 460766 (50.5) | 44966 (45.0) | 338370 (55.3) |
| Age | Mean (SD) | 39.2 (15.9) | 51.8 (16.9) | 58.5 (15.6) | 27.4 (9.5) | 47.7 (19.1) |
|  | Median (IQR) | 36 (22) | 48 (21) | 58 (21) | 25 (12) | 45 (36) |
| Age group | 18-64 | 277372 (92.1) | 26871 (78.9) | 604692 (66.2) | 98824 (98.9) | 444109 (72.6) |
|  | 65-79 | 16490 (5.5) | 3787 (11.1) | 202017 (22.1) | 942 (0.9) | 156436 (25.6) |
|  | 80+ | 7402 (2.5) | 3386 (9.9) | 106569 (11.7) | 206 (0.2) | 11175 (1.8) |
| Deprivation Status † | 1 - High | 80418 (26.7) | 9191 (27.0) | 171471 (18.8) | 25249 (25.3) | 117603 (19.2) |
|  | 2 | 65573 (21.8) | 7496 (22.0) | 184415 (20.2) | 23080 (23.1) | 122952 (20.1) |
|  | 3 | 64559 (21.4) | 6382 (18.7) | 180125 (19.7) | 18380 (18.4) | 112462 (18.4) |
|  | 4 | 47877 (15.9) | 5702 (16.7) | 182091 (19.9) | 16739 (16.7) | 113413 (18.5) |
|  | 5 - Low | 42837 (14.2) | 5273 (15.5) | 195176 (21.4) | 16524 (16.5) | 145290 (23.8) |
| Local authority | Blaenau Gwent | 5434 (1.8) | 635 (1.9) | 30820 (3.4) | 2709 (2.7) | 10027 (1.6) |
|  | Bridgend | 13196 (4.4) | 1576 (4.6) | 53427 (5.9) | 7713 (7.7) | 35730 (5.8) |
|  | Caerphilly | 12598 (4.2) | 1357 (4.0) | 55651 (6.1) | 6422 (6.4) | 40706 (6.7) |
|  | Cardiff | 43861 (14.6) | 3898 (11.4) | 97466 (10.7) | 7388 (7.4) | 85794 (14.0) |
|  | Carmarthenshire | 26776 (8.9) | 2164 (6.4) | 59035 (6.5) | 4368 (4.4) | 21089 (3.4) |
|  | Ceredigion | 16201 (5.4) | 731 (2.1) | 23242 (2.5) | 1734 (1.7) | 8166 (1.3) |
|  | Conwy | 6886 (2.3) | 917 (2.7) | 20506 (2.2) | 2655 (2.7) | 25456 (4.2) |
|  | Denbighshire | 7650 (2.5) | 1507 (4.4) | 27585 (3.0) | 3392 (3.4) | 9753 (1.6) |
|  | Flintshire | 10510 (3.5) | 2433 (7.1) | 35882 (3.9) | 5056 (5.1) | 28205 (4.6) |
|  | Gwynedd | 10915 (3.6) | 1449 (4.3) | 37916 (4.2) | 4409 (4.4) | 22038 (3.6) |
|  | Isle of Anglesey | 5675 (1.9) | 931 (2.7) | 19539 (2.1) | 2231 (2.2) | 20520 (3.4) |
|  | Merthyr Tydfil | 6707 (2.2) | 700 (2.1) | 19502 (2.1) | 2959 (3.0) | 17639 (2.9) |
|  | Monmouthshire | 3356 (1.1) | 364 (1.1) | 18567 (2.0) | 1509 (1.5) | 12286 (2.0) |
|  | Neath Port Talbot | 13081 (4.3) | 2067 (6.1) | 51122 (5.6) | 5893 (5.9) | 27396 (4.5) |
|  | Newport | 16495 (5.5) | 1337 (3.9) | 36424 (4.0) | 6405 (6.4) | 33348 (5.5) |
|  | Pembrokeshire | 16172 (5.4) | 1382 (4.1) | 39527 (4.3) | 1572 (1.6) | 11920 (1.9) |
|  | Powys | 4408 (1.5) | 599 (1.8) | 19656 (2.2) | 1289 (1.3) | 13644 (2.2) |
|  | Rhondda Cynon Taf | 21598 (7.2) | 2207 (6.5) | 78962 (8.6) | 11516 (11.5) | 62143 (10.2) |
|  | Swansea | 30507 (10.1) | 3225 (9.5) | 75534 (8.3) | 9845 (9.8) | 65700 (10.7) |
|  | Torfaen | 5384 (1.8) | 591 (1.7) | 22221 (2.4) | 2119 (2.1) | 14242 (2.3) |
|  | Vale of Glamorgan | 10556 (3.5) | 1725 (5.1) | 49240 (5.4) | 1697 (1.7) | 22577 (3.7) |
|  | Wrexham | 13298 (4.4) | 2249 (6.6) | 41454 (4.5) | 7091 (7.1) | 23341 (3.8) |
| Health board | Aneurin Bevan UHB | 43267 (14.4) | 4284 (12.6) | 163683 (17.9) | 19164 (19.2) | 110609 (18.1) |
|  | Betsi Cadwaladr UHB | 54934 (18.2) | 9486 (27.9) | 182882 (20.0) | 24834 (24.8) | 129313 (21.1) |
|  | Cardiff and Vale UHB | 54417 (18.1) | 5623 (16.5) | 146706 (16.1) | 9085 (9.1) | 108371 (17.7) |
|  | Cwm Taf UHB | 28305 (9.4) | 2907 (8.5) | 98464 (10.8) | 14475 (14.5) | 79782 (13.0) |
|  | Hywel Dda UHB | 59149 (19.6) | 4277 (12.6) | 121804 (13.3) | 7674 (7.7) | 41175 (6.7) |
|  | Powys THB | 4408 (1.5) | 599 (1.8) | 19656 (2.2) | 1289 (1.3) | 13644 (2.2) |
|  | Swansea Bay UHB | 56784 (18.8) | 6868 (20.2) | 180083 (19.7) | 23451 (23.5) | 128826 (21.1) |
| Number of previous tests | 0 | 254430 (84.5) | 25904 (76.1) | 752010 (82.3) | 73423 (73.4) | 446152 (72.9) |
|  | 1 | 32315 (10.7) | 5055 (14.8) | 115931 (12.7) | 19511 (19.5) | 104695 (17.1) |
|  | 2 | 7525 (2.5) | 1432 (4.2) | 25194 (2.8) | 4597 (4.6) | 28411 (4.6) |
|  | 3-4 | 3199 (1.1) | 805 (2.4) | 10234 (1.1) | 1417 (1.4) | 11930 (2.0) |
|  | 5-9 | 1743 (0.6) | 490 (1.4) | 4487 (0.5) | 446 (0.4) | 5880 (1.0) |
|  | 10+ | 2052 (0.7) | 358 (1.1) | 5422 (0.6) | 578 (0.6) | 14652 (2.4) |
| Urban/Rural classification | Rural town and fringe | 34434 (11.4) | 4205 (12.4) | 128755 (14.1) | 14735 (14.7) | 83138 (13.6) |
|  | Rural town and fringe in a sparse setting | 10022 (3.3) | 1205 (3.5) | 31496 (3.4) | 2234 (2.2) | 14679 (2.4) |
|  | Rural village and dispersed | 13512 (4.5) | 1894 (5.6) | 57843 (6.3) | 4841 (4.8) | 32980 (5.4) |
|  | Rural village and dispersed in a sparse setting | 22944 (7.6) | 2311 (6.8) | 69529 (7.6) | 4054 (4.1) | 30109 (4.9) |
|  | Urban city and town | 209663 (69.6) | 23796 (69.9) | 610671 (66.9) | 71802 (71.8) | 440246 (72.0) |
|  | Urban city and town in a sparse setting | 10689 (3.5) | 633 (1.9) | 14984 (1.6) | 2306 (2.3) | 10568 (1.7) |
| BMI | Underweight | 18060 (6.0) | 1102 (3.2) | 16772 (1.8) | 5888 (5.9) | 13984 (2.3) |
|  | Normal weight | 121397 (40.3) | 10132 (29.8) | 229176 (25.1) | 40175 (40.2) | 179600 (29.4) |
|  | Overweight | 90746 (30.1) | 10770 (31.6) | 303913 (33.3) | 29513 (29.5) | 203115 (33.2) |
|  | Obese | 71061 (23.6) | 12040 (35.4) | 363417 (39.8) | 24396 (24.0) | 215021 (35.2) |
| Smoking status | Ex-smoker | 37037 (12.3) | 6851 (20.1) | 220390 (24.1) | 7917 (7.9) | 118835 (19.4) |
|  | Non-smoker | 129471 (43.0) | 14053 (41.3) | 489286 (53.6) | 48406 (48.4) | 351217 (57.4) |
|  | Smoker | 96826 (32.1) | 12195 (35.8) | 187345 (20.5) | 24049 (24.1) | 113691 (18.6) |
|  | Unknown | 37930 (12.6) | 945 (2.8) | 16257 (1.8) | 19600 (19.6) | 27977 (4.6) |
| Number of people in household | 1 | 34659 (11.5) | 6522 (19.2) | 157481 (17.2) | 5728 (5.7) | 71361 (11.7) |
|  | 2 | 56675 (18.8) | 8328 (24.5) | 312396 (34.2) | 14107 (14.1) | 175794 (28.7) |
|  | 3-5 | 161584 (53.6) | 15835 (46.5) | 395367 (43.3) | 66289 (66.3) | 321370 (52.5) |
|  | 6-10 | 42987 (14.3) | 3057 (9.0) | 43858 (4.8) | 12975 (13.0) | 40465 (6.6) |
|  | 11-30 | 4329 (1.4) | 255 (0.7) | 3677 (0.4) | 714 (0.7) | 2390 (0.4) |
|  | 30-101 | 600 (0.2) | 40 (0.1) | 472 (0.1) | 89 (0.1) | 240 (0.0) |
|  | 101+ | 430 (0.1) | 7 (0.0) | 27 (0.0) | 70 (0.1) | 100 (0.0) |
| Average household age | <18 | 18314 (6.1) | 1125 (3.3) | 10585 (1.2) | 8052 (8.1) | 22960 (3.8) |
|  | 18-29 | 89319 (29.6) | 7687 (22.6) | 108734 (11.9) | 36053 (36.1) | 136513 (22.3) |
|  | 30-64 | 175720 (58.3) | 19165 (56.3) | 537826 (58.9) | 54869 (54.9) | 320731 (52.4) |
|  | 65-79 | 13979 (4.6) | 3576 (10.5) | 178316 (19.5) | 833 (0.8) | 121572 (19.9) |
|  | 80+ | 3932 (1.3) | 2491 (7.3) | 77817 (8.5) | 165 (0.2) | 9944 (1.6) |
| Number of risk groups ‡ | 0 | 201753 (67.0) | 15395 (45.2) | 432316 (47.3) | 68899 (68.9) | 362674 (59.3) |
|  | 1 | 71617 (23.8) | 10090 (29.6) | 266167 (29.1) | 25321 (25.3) | 164235 (26.8) |
|  | 2 | 19067 (6.3) | 4582 (13.5) | 121282 (13.3) | 4720 (4.7) | 52805 (8.6) |
|  | 3 | 5356 (1.8) | 2043 (6.0) | 53142 (5.8) | 736 (0.7) | 18978 (3.1) |
|  | 4 | 1942 (0.6) | 975 (2.9) | 23872 (2.6) | 172 (0.2) | 7789 (1.3) |
|  | 5+ | 1529 (0.5) | 959 (2.8) | 16499 (1.8) | 124 (0.1) | 5239 (0.9) |
| Hypertension | |  | 7979 (2.6) | 111645 (12.2) | 799 (0.8) | 47510 (7.8) |
| Vaccination categories refer to vaccination status at the end of the study period - 30 June 2021. † Deprivation status: Quintiles of the Welsh Index of Multiple Deprivation (SIMD) 2020  ‡ Number of risk groups: Count of QCovid risk groups: [doi:10.1136/bmj.m3731](https://www.bmj.com/content/371/bmj.m3731) | | | | | | |
