# Supplementary material for: Waning of first- and second-dose ChAdOx1 and BNT162b2 COVID-19 vaccinations: a pooled target trial study of 12.9 million individuals in England, Northern Ireland, Scotland and Wales
Source: Int J Epidemiol. 2022 Oct 22;52(1):22–31. doi: 10.1093/ije/dyac199 (PMC9620314; doi:10.1093/ije/dyac199)
Supplement: dyac199_Supplementary_Data [file dyac199_supplementary_data.zip › dyac199_Supplementary_Data/ije-2022-04-0492-File011.docx]

**S4 Event count tables**

Tables S4a-S4d give event counts by vaccination status in each country. As an example, the ‘14 days prior to hospitalisation’ row counts the number of people who were hospitalised when their vaccination status 14 days prior to hospitalisation was as given in the column heading.

**Table S4a: Event counts and rates England**

| **Event** | **Unvaccinated** | **One dose ChAdOx1** | **Two doses ChAdOx1** | **One dose BNT162b2** | **Two doses BNT162b2** |
| --- | --- | --- | --- | --- | --- |
| **Person years (thousands)** | 1673.23 | 469.21 | 234.17 | 265.09 | 223.66 |
| **Hospitalisation** | 16,585 (9.91) | 1,320 (2.81) | 133 (0.57) | 1,441 (5.44) | 215 (0.96) |
| **14 days prior to hospitalisation** | 17,651 (10.55) | 806 (1.72) | 113 (0.48) | 964 (3.64) | 160 (0.72) |
| **Death** | 4,224 (2.52) | 395 (0.84) | 24 (0.10) | 441 (1.66) | 48 (0.21) |
| **14 days prior to death** | 4,335 (2.59) | 332 (0.71) | 22 (0.09) | 403 (1.52) | 40 (0.18) |
| **Hospitalisation or death** | 17,703 (10.58) | 1,413 (3.01) | 140 (0.60) | 1,519 (5.73) | 225 (1.01) |
| **14 days prior to hospitalisation or death** | 18,812 (11.24) | 876 (1.87) | 118 (0.50) | 1,025 (3.87) | 169 (0.76) |
| Parentheses give event rates per thousand person years | | | | | |

**Table S4b: Event counts and rates Northern Ireland**

| **Event** | **Unvaccinated** | **One dose ChAdOx1** | **Two doses ChAdOx1** | **One dose BNT162b2** | **Two doses BNT162b2** |
| --- | --- | --- | --- | --- | --- |
| **Person years (thousands)** | 477.51 | 111.57 | 71.02 | 79.36 | 63.68 |
| **Hospitalisation** | 3,646 (7.64) | 422 (3.78) | * | * | * |
| **14 days prior to hospitalisation** | 3,819 (8.00) | 304 (2.72) | * | * | * |
| **Death** | 876 (1.83) | 126 (1.13) | * | * | * |
| **14 days prior to death** | 895 (1.87) | 107 (0.96) | * | * | * |
| **Hospitalisation or death** | 3,856 (8.08) | 443 (3.97) | 51 (0.72) | 91 (1.15) | 11 (0.17) |
| **14 days prior to hospitalisation or death** | 4,036 (8.45) | 318 (2.85) | 39 (0.55) | 51 (0.64) | * |
| Parentheses give event rates per thousand person years  * Indicates a count of <10. | | | | | |

**Table S4c: Event counts and rates Scotland**

| **Event** | **Unvaccinated** | **One dose ChAdOx1** | **Two doses ChAdOx1** | **One dose BNT162b2** | **Two doses BNT162b2** |
| --- | --- | --- | --- | --- | --- |
| **Person years (thousands)** | 1401.83 | 383.7 | 222.01 | 206.55 | 177.8 |
| **Hospitalisation** | 9,677 (6.90) | 1,512 (3.94) | 526 (2.37) | 510 (2.47) | 104 (0.58) |
| **14 days prior to hospitalisation** | 10,465 (7.47) | 1,085 (2.83) | 369 (1.66) | 336 (1.63) | 74 (0.42) |
| **Death** | 2,749 (1.96) | 375 (0.98) | 72 (0.32) | 180 (0.87) | 18 (0.10) |
| **14 days prior to death** | 2,832 (2.02) | 319 (0.83) | 68 (0.31) | 160 (0.77) | 15 (0.08) |
| **Hospitlisation or death** | 10,561 (7.53) | 1,558 (4.06) | 532 (2.40) | 614 (2.97) | 111 (0.62) |
| **14 days prior to hospitalisation or death** | 11,373 (8.11) | 1,120 (2.92) | 374 (1.68) | 430 (2.08) | 79 (0.44) |
| Parentheses give event rates per thousand person years | | | | | |

**Table S4d: Event counts and rates Wales**

| **Event** | **Unvaccinated** | **One dose ChAdOx1** | **Two doses ChAdOx1** | **One dose BNT162b2** | **Two doses BNT162b2** |
| --- | --- | --- | --- | --- | --- |
| **Person years (thousands)** | 593.37 | 193.06 | 98.94 | 92.57 | 101.74 |
| **Hospitalisation** | 8,207 (13.83) | 790 (4.09) | 119 (0.01) | 260 (2.81) | 62 (0.61) |
| **14 days prior to hospitalisation** | 8,209 (13.83) | 553 (2.86) | 88 (0.89) | 178 (1.92) | 50 (0.49) |
| **Death** | 1,741 (2.94) | 172 (0.89) | 10 (0.10) | 26 (0.28) | 5 (0.05) |
| **14 days prior to death** | 1,741 (2.94) | 146 (0.76) | 7 (0.06) | 21 (0.23) | * |
| **Hospitlisation or death** | 8,411 (14.17) | 807 (4.18) | 122 (1.03) | 263 (2.84) | 62 (0.61) |
| **14 days prior to hospitalisation or death** | 8,413 (14.18) | 566 (7.11) | 89 (0.90) | 180 (1.94) | 50 (0.49) |
| Parentheses give event rates per thousand person years  * Indicates a count of <5. | | | | | |
