# Supplementary material for: Waning of first- and second-dose ChAdOx1 and BNT162b2 COVID-19 vaccinations: a pooled target trial study of 12.9 million individuals in England, Northern Ireland, Scotland and Wales
Source: Int J Epidemiol. 2022 Oct 22;52(1):22–31. doi: 10.1093/ije/dyac199 (PMC9620314; doi:10.1093/ije/dyac199)
Supplement: dyac199_Supplementary_Data [file dyac199_supplementary_data.zip › dyac199_Supplementary_Data/ije-2022-04-0492-File012.docx]

**S5 Exposure group summary tables**

**Table S5a: England first dose matching summary table**

| **Characteristic** | **Level** | **Unvaccinated controls ChAdOx1** | **First dose recipient ChAdOx1** | **Unvaccinated controls BNT162b2** | **First dose recipient** **BNT162b2** |
| --- | --- | --- | --- | --- | --- |
| Total | | 1,241,591 | 1,241,591 | 1,041,757 | 1,041,757 |
| COVID-19 hospitalisation or death | | 1012 | 426 | 1766 | 579 |
| Sex | Female | 622100 (50.1%) | 653677 (52.6%) | 579493 (55.6%) | 594036 (57.0%) |
|  | Male | 619491 (49.9%) | 587914 (47.4%) | 462264 (44.4%) | 447721 (43.0%) |
| Age group (years) | 18-64 | 930045 (74.9%) | 930045 (74.9%) | 733227 (70.4%) | 733227 (70.4%) |
|  | 65-79 | 273871 (22.1%) | 273871 (22.1%) | 176385 (16.9%) | 176385 (16.9%) |
|  | 80+ | 37675 (3.0%) | 37675 (3.0%) | 132145 (12.7%) | 132145 (12.7%) |
| Deprivation status † | 1 - High | 239336 (19.3%) | 235272 (18.9%) | 196585 (18.9%) | 189845 (18.2%) |
|  | 2 | 276232 (22.2%) | 266096 (21.4%) | 232996 (22.4%) | 234493 (22.5%) |
|  | 3 | 263874 (21.3%) | 267586 (21.6%) | 224218 (21.5%) | 223018 (21.4%) |
|  | 4 | 241240 (19.4%) | 247316 (19.9%) | 209209 (20.1%) | 211541 (20.3%) |
|  | 5-Low | 220909 (17.8%) | 225321 (18.1%) | 178749 (17.2%) | 182860 (17.6%) |
|  | Unknown | 239336 (19.3%) | 235272 (18.9%) | 196585 (18.9%) | 189845 (18.2%) |
| Urban/Rural index | Large Urban Areas | 360706 (29.1%) | 360757 (29.1%) | 326548 (31.3%) | 329807 (31.7%) |
|  | Other Urban Areas | 44054 (3.5%) | 44172 (3.6%) | 33784 (3.2%) | 35504 (3.4%) |
|  | Rural town or village | 220896 (17.8%) | 221200 (17.8%) | 171808 (16.5%) | 176085 (16.9%) |
|  | Urban city or town | 615935 (49.6%) | 615462 (49.6%) | 509617 (48.9%) | 500361 (48.0%) |
| Number of risk groups ‡ | 0 | 706362 (56.9%) | 661288 (53.3%) | 554570 (53.2%) | 541589 (52.0%) |
|  | 1 | 345371 (27.8%) | 370096 (29.8%) | 291910 (28.0%) | 301700 (29.0%) |
|  | 2 | 121539 (9.8%) | 133899 (10.8%) | 113990 (10.9%) | 118239 (11.3%) |
|  | 3 | 41834 (3.4%) | 46542 (3.7%) | 46073 (4.4%) | 45996 (4.4%) |
|  | 4 | 16149 (1.3%) | 18024 (1.5%) | 20358 (2.0%) | 20058 (1.9%) |
|  | 5+ | 10336 (0.8%) | 11742 (0.9%) | 14856 (1.4%) | 14175 (1.4%) |
| Number of previous tests | 0 | 871467 (70.2%) | 807278 (65.0%) | 680900 (65.4%) | 658142 (63.2%) |
|  | 1 | 194913 (15.7%) | 219603 (17.7%) | 177082 (17.0%) | 186764 (17.9%) |
|  | 2 | 90809 (7.3%) | 105413 (8.5%) | 86759 (8.3%) | 91205 (8.8%) |
|  | 3 | 36114 (2.9%) | 45652 (3.7%) | 37871 (3.6%) | 40841 (3.9%) |
|  | 4-9 | 31312 (2.5%) | 42610 (3.4%) | 34546 (3.3%) | 38600 (3.7%) |
|  | 10+ | 16976 (1.4%) | 21035 (1.7%) | 24599 (2.4%) | 26205 (2.5%) |
| Number of people in household | 1 | 324888 (26.2%) | 300652 (24.2%) | 280461 (26.9%) | 271534 (26.1%) |
|  | 2 | 368403 (29.7%) | 367808 (29.6%) | 306784 (29.4%) | 308120 (29.6%) |
|  | 3-5 | 475929 (38.3%) | 503719 (40.6%) | 388710 (37.3%) | 401733 (38.6%) |
|  | 6-10 | 64834 (5.2%) | 62569 (5.0%) | 55168 (5.3%) | 52212 (5.0%) |
|  | 11-30 | 5615 (0.5%) | 5086 (0.4%) | 5064 (0.5%) | 4044 (0.4%) |
|  | 31-100 | 917 (0.1%) | 856 (0.1%) | 982 (0.1%) | 702 (0.1%) |
|  | 101+ | 1005 (0.1%) | 901 (0.1%) | 4588 (0.4%) | 3412 (0.3%) |
| BMI | Underweight | 26864 (2.2%) | 29189 (2.4%) | 55462 (5.3%) | 53979 (5.2%) |
|  | Normal weight | 437661 (35.3%) | 433998 (35.0%) | 418809 (40.2%) | 419091 (40.2%) |
|  | Overweight | 448560 (36.1%) | 436207 (35.1%) | 330588 (31.7%) | 328831 (31.6%) |
|  | Obese | 328506 (26.5%) | 342197 (27.6%) | 236898 (22.7%) | 239856 (23.0%) |
| † Deprivation status: Quintiles of the English Index of Deprivation 2019  ‡ Number of risk groups: Count of QCovid risk groups: [doi:10.1136/bmj.m3731](https://www.bmj.com/content/371/bmj.m3731) | | | | | |

**Table S5b: England second dose matching summary table**

| **Characteristic** | **Level** | **First dose controls ChAdOx1** | **Second dose recipient ChAdOx1** | **First dose controls BNT162b2** | **Second dose recipient** **BNT162b2** |
| --- | --- | --- | --- | --- | --- |
| Total | | 1,057,259 | 1,057,259 | 600,802 | 600,802 |
| COVID-19 hospitalisation or death | | 105 | 52 | 264 | 109 |
| Sex | Female | 560574 (53.0%) | 333966 (55.6%) | 361634 (60.2%) | 547246 (51.8%) |
|  | Male | 496685 (47.0%) | 266836 (44.4%) | 239168 (39.8%) | 510013 (48.2%) |
| Age group (years) | 18-64 | 744442 (70.4%) | 744442 (70.4%) | 332656 (55.4%) | 332656 (55.4%) |
|  | 65-79 | 268254 (25.4%) | 268254 (25.4%) | 153696 (25.6%) | 153696 (25.6%) |
|  | 80+ | 44563 (4.2%) | 44563 (4.2%) | 114450 (19.0%) | 114450 (19.0%) |
| Deprivation status † | 1 - High | 198762 (18.8%) | 190561 (18.0%) | 104668 (17.4%) | 106156 (17.7%) |
|  | 2 | 227420 (21.5%) | 223330 (21.1%) | 134038 (22.3%) | 133383 (22.2%) |
|  | 3 | 224148 (21.2%) | 227701 (21.5%) | 128895 (21.5%) | 128132 (21.3%) |
|  | 4 | 210088 (19.9%) | 223986 (21.2%) | 123004 (20.5%) | 124549 (20.7%) |
|  | 5-Low | 196841 (18.6%) | 191681 (18.1%) | 110197 (18.3%) | 108582 (18.1%) |
|  | Unknown |  |  |  |  |
| Urban/Rural index | Large Urban Areas | 288635 (27.3%) | 289022 (27.3%) | 173189 (28.8%) | 176574 (29.4%) |
|  | Other Urban Areas | 38897 (3.7%) | 40136 (3.8%) | 21338 (3.6%) | 20937 (3.5%) |
|  | Accessible Small Towns | 193207 (18.3%) | 205338 (19.4%) | 110842 (18.4%) | 110972 (18.5%) |
|  | Remote Small Towns | 536520 (50.7%) | 522763 (49.4%) | 295433 (49.2%) | 292319 (48.7%) |
| Number of risk groups ‡ | 0 | 562070 (53.2%) | 542546 (51.3%) | 255266 (42.5%) | 253387 (42.2%) |
|  | 1 | 310030 (29.3%) | 317836 (30.1%) | 186339 (31.0%) | 188662 (31.4%) |
|  | 2 | 115503 (10.9%) | 122394 (11.6%) | 90941 (15.1%) | 90793 (15.1%) |
|  | 3 | 41885 (4.0%) | 44820 (4.2%) | 39495 (6.6%) | 38907 (6.5%) |
|  | 4 | 16596 (1.6%) | 18021 (1.7%) | 17031 (2.8%) | 17070 (2.8%) |
|  | 5+ | 11175 (1.1%) | 11642 (1.1%) | 11730 (2.0%) | 11983 (2.0%) |
| Number of previous tests | 0 | 715048 (67.6%) | 693847 (65.6%) | 412981 (68.7%) | 396085 (65.9%) |
|  | 1 | 178051 (16.8%) | 185302 (17.5%) | 93071 (15.5%) | 98203 (16.3%) |
|  | 2 | 82246 (7.8%) | 86605 (8.2%) | 41305 (6.9%) | 43723 (7.3%) |
|  | 3 | 33954 (3.2%) | 36849 (3.5%) | 17693 (2.9%) | 19171 (3.2%) |
|  | 4-9 | 29950 (2.8%) | 34598 (3.3%) | 17316 (2.9%) | 20555 (3.4%) |
|  | 10+ | 18010 (1.7%) | 20058 (1.9%) | 18436 (3.1%) | 23065 (3.8%) |
| Number of people in household | 1 | 273806 (25.9%) | 261610 (24.7%) | 175752 (29.3%) | 170759 (28.4%) |
|  | 2 | 335813 (31.8%) | 338380 (32.0%) | 205807 (34.3%) | 208584 (34.7%) |
|  | 3-5 | 397514 (37.6%) | 407104 (38.5%) | 193395 (32.2%) | 197376 (32.9%) |
|  | 6-10 | 45478 (4.3%) | 45566 (4.3%) | 23333 (3.9%) | 22070 (3.7%) |
|  | 11-30 | 3493 (0.3%) | 3521 (0.3%) | 1646 (0.3%) | 1324 (0.2%) |
|  | 31-100 | 590 (0.1%) | 549 (0.1%) | 258 (0.0%) | 254 (0.0%) |
|  | 101+ | 565 (0.1%) | 529 (0.1%) | 611 (0.1%) | 435 (0.1%) |
| BMI | Underweight | 20389 (1.9%) | 20904 (2.0%) | 16676 (2.8%) | 14880 (2.5%) |
|  | Normal weight | 353420 (33.4%) | 353045 (33.4%) | 210205 (35.0%) | 211608 (35.2%) |
|  | Overweight | 384705 (36.4%) | 380265 (36.0%) | 210540 (35.0%) | 208167 (34.6%) |
|  | Obese | 298745 (28.3%) | 303045 (28.7%) | 163381 (27.2%) | 166147 (27.7%) |
| † Deprivation status: Quintiles of the English Index of Deprivation 2019  ‡ Number of risk groups: Count of QCovid risk groups: [doi:10.1136/bmj.m3731](https://www.bmj.com/content/371/bmj.m3731) | | | | | |

**Table S5c: Northern Ireland first dose matching summary table**

| **Characteristic** | **Level** | **Unvaccinated controls ChAdOx1** | **First dose recipient ChAdOx1** | **Unvaccinated controls BNT162b2** | **First dose recipient BNT162b2** |
| --- | --- | --- | --- | --- | --- |
| Total | | 320,258 | 320,258 | 320,706 | 320,706 |
| COVID-19 hospitalisation or death | | 322 (0.1) | 129 (0.0) | 173 (0.1) | 54 (0.0) |
| Sex | Female | 157012 (49.0) | 155718 (48.6) | 171011 (53.3) | 182190 (56.8) |
|  | Male | 163246 (51.0) | 164540 (51.4) | 149695 (46.7) | 138516 (43.2) |
| Age group (years) | <65 | 217979 (68.1) | 217979 (68.1) | 281595 (87.8) | 281595 (87.8) |
|  | 65-79 | 69415 (21.7) | 69415 (21.7) | 39094 (12.2) | 39094 (12.2) |
|  | 80+ | 32864 (10.3) | 32864 (10.3) | 17 (0.0) | 17 (0.0) |
| Deprivation Status† | 1 - High | 29038 (9.1) | 30639 (9.6) | 27058 (8.4) | 25568 (8.0) |
|  | 2 | 31052 (9.7) | 33244 (10.4) | 29166 (9.1) | 27049 (8.4) |
|  | 3 | 31338 (9.8) | 32439 (10.1) | 29810 (9.3) | 29018 (9.0) |
|  | 4 | 33185 (10.4) | 34190 (10.7) | 33191 (10.3) | 32604 (10.2) |
|  | 5 | 33270 (10.4) | 34072 (10.6) | 33596 (10.5) | 33359 (10.4) |
|  | 6 | 31344 (9.8) | 31149 (9.7) | 32349 (10.1) | 31725 (9.9) |
|  | 7 | 33059 (10.3) | 31799 (9.9) | 35309 (11.0) | 36548 (11.4) |
|  | 8 | 33419 (10.4) | 31355 (9.8) | 35128 (11.0) | 36757 (11.5) |
|  | 9 | 31834 (9.9) | 30018 (9.4) | 32624 (10.2) | 34146 (10.6) |
|  | 10 - Low | 32719 (10.2) | 31353 (9.8) | 32475 (10.1) | 33932 (10.6) |
| Settlement band | A Belfast Metropolitan Urban Area | 51388 (16.0) | 51555 (16.1) | 45548 (14.2) | 45505 (14.2) |
|  | B Derry Urban Area | 15445 (4.8) | 15332 (4.8) | 15949 (5.0) | 16262 (5.1) |
|  | C Large town | 94359 (29.5) | 95346 (29.8) | 94182 (29.4) | 93376 (29.1) |
|  | D Medium town | 21336 (6.7) | 23433 (7.3) | 22982 (7.2) | 21968 (6.8) |
|  | E Small town | 20535 (6.4) | 20564 (6.4) | 20738 (6.5) | 20573 (6.4) |
|  | F Intermediate settlement | 13317 (4.2) | 13302 (4.2) | 14096 (4.4) | 14146 (4.4) |
|  | G Village | 17858 (5.6) | 17816 (5.6) | 17999 (5.6) | 17753 (5.5) |
|  | H Small village, hamlet and open countryside | 86020 (26.9) | 82910 (25.9) | 89212 (27.8) | 91123 (28.4) |
| BNF chapters prescribed‡ | 0 | 136808 (42.7) | 127826 (39.9) | 190515 (59.4) | 195542 (61.0) |
|  | 1 | 61499 (19.2) | 59980 (18.7) | 57395 (17.9) | 58372 (18.2) |
|  | 2 | 45777 (14.3) | 48248 (15.1) | 33267 (10.4) | 32502 (10.1) |
|  | 3 | 33598 (10.5) | 36407 (11.4) | 19677 (6.1) | 18039 (5.6) |
|  | 4 | 22382 (7.0) | 24756 (7.7) | 11084 (3.5) | 9325 (2.9) |
|  | 5 | 12347 (3.9) | 13913 (4.3) | 5544 (1.7) | 4406 (1.4) |
|  | 6+ | 7847 (2.5) | 9128 (2.9) | 3224 (1.0) | 2520 (0.8) |
| Number of previous tests | 0 | 252212 (78.8) | 253224 (79.1) | 234152 (73.0) | 227711 (71.0) |
|  | 1 | 48885 (15.3) | 47939 (15.0) | 58490 (18.2) | 60397 (18.8) |
|  | 2 | 12455 (3.9) | 12408 (3.9) | 15802 (4.9) | 17523 (5.5) |
|  | 3 | 3688 (1.2) | 3532 (1.1) | 4677 (1.5) | 5508 (1.7) |
|  | 4-9 | 2799 (0.9) | 2879 (0.9) | 5897 (1.8) | 7427 (2.3) |
|  | 10+ | 219 (0.1) | 276 (0.1) | 1688 (0.5) | 2140 (0.7) |
| Number of people in household | 1 | 50667 (15.8) | 52013 (16.2) | 30747 (9.6) | 27389 (8.5) |
|  | 2 | 84387 (26.3) | 86522 (27.0) | 62778 (19.6) | 61844 (19.3) |
|  | 3-5 | 157537 (49.2) | 153854 (48.0) | 191864 (59.8) | 194678 (60.7) |
|  | 6-10 | 26696 (8.3) | 26780 (8.4) | 33862 (10.6) | 35368 (11.0) |
|  | 11-30 | 856 (0.3) | 987 (0.3) | 1124 (0.4) | 1081 (0.3) |
|  | 31-100 | 56 (0.0) | 56 (0.0) | 84 (0.0) | 69 (0.0) |
|  | 101+ | 59 (0.0) | 46 (0.0) | 247 (0.1) | 277 (0.1) |
| † Derivation Status: Deciles of the Northern Ireland Multiple Deprivation Measure 2017.  ‡ BNF chapters prescribed: Number of chapters of the British National Formulary (BNF) from which individuals received repeat prescriptions prior to vaccination. | | | | | |

**Table S5d: Northern Ireland second dose matching summary table**

| **Characteristic** | **Level** | **First dose controls ChAdOx1** | **Second dose recipient ChAdOx1** | **First dose controls BNT162b2** | **Second dose recipient BNT162b2** |
| --- | --- | --- | --- | --- | --- |
| Total |  | 200805 | 200805 | 193015 | 193015 |
| COVID-19 hospitalisation or death | | 21 (0.0) | * | 18 (0.0) | * |
| Sex | Female | 102165 (50.9) | 100989 (50.3) | 110125 (57.1) | 117450 (60.9) |
|  | Male | 98640 (49.1) | 99816 (49.7) | 82890 (42.9) | 75565 (39.1) |
| Age group (years) | <65 | 109934 (54.7) | 109934 (54.7) | 158456 (82.1) | 158456 (82.1) |
|  | 65-79 | 62576 (31.2) | 62576 (31.2) | 34537 (17.9) | 34537 (17.9) |
|  | 80+ | 28295 (14.1) | 28295 (14.1) | 22 (0.0) | 22 (0.0) |
| Deprivation Status† | 1 - High | 15400 (7.7) | 15977 (8.0) | 15443 (8.0) | 13754 (7.1) |
|  | 2 | 18113 (9.0) | 18752 (9.3) | 16861 (8.7) | 15139 (7.8) |
|  | 3 | 19046 (9.5) | 19191 (9.6) | 17488 (9.1) | 16474 (8.5) |
|  | 4 | 20518 (10.2) | 20700 (10.3) | 19524 (10.1) | 18816 (9.7) |
|  | 5 | 20943 (10.4) | 21139 (10.5) | 19970 (10.3) | 19731 (10.2) |
|  | 6 | 19997 (10.0) | 20218 (10.1) | 19071 (9.9) | 18932 (9.8) |
|  | 7 | 21123 (10.5) | 20744 (10.3) | 21087 (10.9) | 21685 (11.2) |
|  | 8 | 22030 (11.0) | 21393 (10.7) | 21387 (11.1) | 22961 (11.9) |
|  | 9 | 21122 (10.5) | 20544 (10.2) | 20761 (10.8) | 22174 (11.5) |
|  | 10 -Low | 22513 (11.2) | 22147 (11.0) | 21423 (11.1) | 23349 (12.1) |
| Settlement band | A Belfast Metropolitan Urban Area | 29409 (14.6) | 29864 (14.9) | 27526 (14.3) | 27396 (14.2) |
|  | B Derry Urban Area | 8564 (4.3) | 8270 (4.1) | 9587 (5.0) | 9844 (5.1) |
|  | C Large town | 60866 (30.3) | 59850 (29.8) | 57781 (29.9) | 56734 (29.4) |
|  | D Medium town | 13033 (6.5) | 14249 (7.1) | 13104 (6.8) | 12569 (6.5) |
|  | E Small town | 13251 (6.6) | 13608 (6.8) | 12287 (6.4) | 12485 (6.5) |
|  | F Intermediate settlement | 8979 (4.5) | 8827 (4.4) | 8504 (4.4) | 8722 (4.5) |
|  | G Village | 10936 (5.4) | 11158 (5.6) | 10770 (5.6) | 10416 (5.4) |
|  | H Small village, hamlet and open countryside | 55767 (27.8) | 54979 (27.4) | 53456 (27.7) | 54849 (28.4) |
| BNF chapters prescribed‡ | 0 | 57432 (28.6) | 53973 (26.9) | 92185 (47.8) | 95232 (49.3) |
|  | 1 | 41067 (20.5) | 41370 (20.6) | 41993 (21.8) | 42476 (22.0) |
|  | 2 | 36433 (18.1) | 37214 (18.5) | 26409 (13.7) | 26152 (13.5) |
|  | 3 | 28292 (14.1) | 29070 (14.5) | 16108 (8.3) | 15097 (7.8) |
|  | 4 | 19590 (9.8) | 20277 (10.1) | 9149 (4.7) | 8019 (4.2) |
|  | 5 | 10910 (5.4) | 11595 (5.8) | 4510 (2.3) | 3803 (2.0) |
|  | 6+ | 7081 (3.5) | 7306 (3.6) | 2661 (1.4) | 2236 (1.2) |
| Number of previous tests | 0 | 159705 (79.5) | 160594 (80.0) | 141034 (73.1) | 136012 (70.5) |
|  | 1 | 29635 (14.8) | 29191 (14.5) | 34678 (18.0) | 37031 (19.2) |
|  | 2 | 7426 (3.7) | 7309 (3.6) | 9412 (4.9) | 10526 (5.5) |
|  | 3 | 2224 (1.1) | 2069 (1.0) | 2864 (1.5) | 3370 (1.7) |
|  | 4-9 | 1702 (0.8) | 1552 (0.8) | 3830 (2.0) | 4695 (2.4) |
|  | 10+ | 113 (0.1) | 90 (0.0) | 1197 (0.6) | 1381 (0.7) |
| Number of people in household | 1 | 36834 (18.3) | 36743 (18.3) | 21133 (10.9) | 18443 (9.6) |
|  | 2 | 63131 (31.4) | 64002 (31.9) | 43508 (22.5) | 43205 (22.4) |
|  | 3-5 | 87886 (43.8) | 87361 (43.5) | 111250 (57.6) | 113601 (58.9) |
|  | 6-10 | 12559 (6.3) | 12260 (6.1) | 16534 (8.6) | 17202 (8.9) |
|  | 11-30 | 375 (0.2) | 414 (0.2) | 521 (0.3) | 483 (0.3) |
|  | 31-100 | * | * | 26 (0.0) | 30 (0.0) |
|  | 101+ | * | * | 43 (0.0) | 51 (0.0) |
| † Derivation Status: Deciles of the Northern Ireland Multiple Deprivation Measure 2017.  ‡ BNF chapters prescribed: Number of chapters of the British National Formulary (BNF) from which individuals received repeat prescriptions prior to vaccination.  * Indicates suppression due to low numbers | | | | | |

**Table S5e: Scotland first dose matching summary table**

| **Characteristic** | **Level** | **Unvaccinated controls ChAdOx1** | **First dose recipient ChAdOx1** | **Unvaccinated controls BNT162b2** | **First dose recipient BNT162b2** |
| --- | --- | --- | --- | --- | --- |
| Total | | 939007 | 939007 | 711081 | 711081 |
| COVID-19 hospitalisation or death | | 525 (0.1) | 309 (0.0) | 463 (0.1) | 229 (0.0) |
| Sex | Female | 471528 (50.2) | 474885 (50.6) | 397742 (55.9) | 407793 (57.3) |
|  | Male | 467479 (49.8) | 464122 (49.4) | 313339 (44.1) | 303288 (42.7) |
| Age group (years) | 18-64 | 679814 (72.4) | 679814 (72.4) | 539244 (75.8) | 539244 (75.8) |
|  | 65-79 | 172840 (18.4) | 172840 (18.4) | 159492 (22.4) | 159492 (22.4) |
|  | 80+ | 86353 (9.2) | 86353 (9.2) | 12345 (1.7) | 12345 (1.7) |
| Deprivation status † | 1 - High | 168378 (17.9) | 171496 (18.3) | 133006 (18.7) | 130487 (18.4) |
|  | 2 | 183221 (19.5) | 183228 (19.5) | 139489 (19.6) | 139720 (19.6) |
|  | 3 | 191592 (20.4) | 191815 (20.4) | 144065 (20.3) | 140666 (19.8) |
|  | 4 | 197796 (21.1) | 193468 (20.6) | 149329 (21.0) | 151212 (21.3) |
|  | 5-Low | 193102 (20.6) | 194082 (20.7) | 141058 (19.8) | 144862 (20.4) |
|  | Missing | 4918 (0.5) | 4918 (0.5) | 4134 (0.6) | 4134 (0.6) |
| Urban/Rural classification | Large Urban Areas | 288975 (30.8) | 290450 (30.9) | 228634 (32.2) | 227992 (32.1) |
|  | Other Urban Areas | 355173 (37.8) | 356078 (37.9) | 271732 (38.2) | 279150 (39.3) |
|  | Accessible Small Towns | 94761 (10.1) | 94115 (10.0) | 68959 (9.7) | 65977 (9.3) |
|  | Remote Small Towns | 49452 (5.3) | 50507 (5.4) | 34796 (4.9) | 33329 (4.7) |
|  | Accessible Rural | 96407 (10.3) | 93466 (10.0) | 63905 (9.0) | 61596 (8.7) |
|  | Remote Rural | 49321 (5.3) | 49473 (5.3) | 38921 (5.5) | 38903 (5.5) |
|  | Unknown | 4918 (0.5) | 4918 (0.5) | 4134 (0.6) | 4134 (0.6) |
| Number of risk groups ‡ | 0 | 477582 (50.9) | 468887 (49.9) | 427254 (60.1) | 428926 (60.3) |
|  | 1 | 275800 (29.4) | 275904 (29.4) | 184946 (26.0) | 189158 (26.6) |
|  | 2 | 112383 (12.0) | 114857 (12.2) | 62125 (8.7) | 60706 (8.5) |
|  | 3 | 43300 (4.6) | 46217 (4.9) | 22661 (3.2) | 20434 (2.9) |
|  | 4 | 18107 (1.9) | 20155 (2.1) | 8916 (1.3) | 7572 (1.1) |
|  | 5+ | 11835 (1.3) | 12987 (1.4) | 5179 (0.7) | 4285 (0.6) |
| Number of previous tests | 0 | 784757 (83.6) | 782841 (83.4) | 549033 (77.2) | 544021 (76.5) |
|  | 1 | 111576 (11.9) | 113263 (12.1) | 104562 (14.7) | 107019 (15.1) |
|  | 2 | 24116 (2.6) | 24606 (2.6) | 24110 (3.4) | 24910 (3.5) |
|  | 3 | 7192 (0.8) | 7336 (0.8) | 7270 (1.0) | 7312 (1.0) |
|  | 4-9 | 8157 (0.9) | 8090 (0.9) | 11231 (1.6) | 11475 (1.6) |
|  | 10+ | 3209 (0.3) | 2871 (0.3) | 14875 (2.1) | 16344 (2.3) |
| BMI | Underweight | 8760 (0.9) | 9049 (1.0) | 7015 (1.0) | 6738 (0.9) |
|  | Normal weight | 111592 (11.9) | 114919 (12.2) | 94112 (13.2) | 94183 (13.2) |
|  | Overweight | 620905 (66.1) | 615136 (65.5) | 482953 (67.9) | 483156 (67.9) |
|  | Obese | 197750 (21.1) | 199903 (21.3) | 127001 (17.9) | 127004 (17.9) |
| Number of people in household | 1 | 317298 (33.8) | 313783 (33.4) | 205771 (28.9) | 202691 (28.5) |
|  | 2 | 304337 (32.4) | 304914 (32.5) | 218594 (30.7) | 220404 (31.0) |
|  | 3-5 | 296981 (31.6) | 300032 (32.0) | 267128 (37.6) | 268175 (37.7) |
|  | 6-10 | 19364 (2.1) | 19310 (2.1) | 18599 (2.6) | 18801 (2.6) |
|  | 11-30 | 791 (0.1) | 784 (0.1) | 769 (0.1) | 778 (0.1) |
|  | 31-100 | 122 (0.0) | 99 (0.0) | 126 (0.0) | 150 (0.0) |
|  | 101+ | 114 (0.0) | 85 (0.0) | 94 (0.0) | 82 (0.0) |
| † Deprivation status: Quintiles of the Scottish Index of Multiple Deprivation (SIMD) 2020  ‡ Number of risk groups: Count of QCovid risk groups: [doi:10.1136/bmj.m3731](https://www.bmj.com/content/371/bmj.m3731) | | | | | |

**Table S5f: Scotland second dose matching summary table**

| **Characteristic** | **Level** | **First dose controls ChAdOx1** | **Second dose recipient ChAdOx1** | **First dose controls BNT162b2** | **Second dose recipient BNT162b2** |
| --- | --- | --- | --- | --- | --- |
| Total | | 884217 (50.0) | 884217 (50.0) | 474069 (50.0) | 474069 (50.0) |
| COVID-19 hospitalisation or death | | 165 (0.0) | 151 (0.0) | 96 (0.0) | 29 (0.0) |
| Sex | Female | 458900 (51.9) | 454001 (51.3) | 289792 (61.1) | 295024 (62.2) |
|  | Male | 425317 (48.1) | 430216 (48.7) | 184277 (38.9) | 179045 (37.8) |
| Age group (years) | 18-64 | 618440 (69.9) | 618440 (69.9) | 314465 (66.3) | 314465 (66.3) |
|  | 65-79 | 179942 (20.4) | 179942 (20.4) | 150281 (31.7) | 150281 (31.7) |
|  | 80+ | 85835 (9.7) | 85835 (9.7) | 9323 (2.0) | 9323 (2.0) |
| Deprivation status † | 1 - High | 161037 (18.2) | 158871 (18.0) | 88528 (18.7) | 86744 (18.3) |
|  | 2 | 173474 (19.6) | 171942 (19.4) | 91756 (19.4) | 92890 (19.6) |
|  | 3 | 183185 (20.7) | 182638 (20.7) | 93676 (19.8) | 93413 (19.7) |
|  | 4 | 184080 (20.8) | 183641 (20.8) | 101630 (21.4) | 101633 (21.4) |
|  | 5-Low | 178116 (20.1) | 182800 (20.7) | 96150 (20.3) | 97060 (20.5) |
|  | Missing | 4325 (0.5) | 4325 (0.5) | 2329 (0.5) | 2329 (0.5) |
| Urban/Rural classification | Large Urban Areas | 269512 (30.5) | 268748 (30.4) | 151292 (31.9) | 152095 (32.1) |
|  | Other Urban Areas | 330557 (37.4) | 333002 (37.7) | 181893 (38.4) | 184239 (38.9) |
|  | Accessible Small Towns | 90698 (10.3) | 89561 (10.1) | 47642 (10.0) | 45001 (9.5) |
|  | Remote Small Towns | 50309 (5.7) | 48689 (5.5) | 22503 (4.7) | 22996 (4.9) |
|  | Accessible Rural | 89657 (10.1) | 90206 (10.2) | 41851 (8.8) | 40610 (8.6) |
|  | Remote Rural | 49159 (5.6) | 49686 (5.6) | 26559 (5.6) | 26799 (5.7) |
|  | Unknown | 4325 (0.5) | 4325 (0.5) | 2329 (0.5) | 2329 (0.5) |
| Number of risk groups ‡ | 0 | 425569 (48.1) | 420241 (47.5) | 260776 (55.0) | 262286 (55.3) |
|  | 1 | 266255 (30.1) | 266101 (30.1) | 135847 (28.7) | 135330 (28.5) |
|  | 2 | 115818 (13.1) | 115803 (13.1) | 49476 (10.4) | 49319 (10.4) |
|  | 3 | 45497 (5.1) | 48236 (5.5) | 17824 (3.8) | 17305 (3.7) |
|  | 4 | 19096 (2.2) | 20748 (2.3) | 6563 (1.4) | 6420 (1.4) |
|  | 5+ | 11982 (1.4) | 13088 (1.5) | 3583 (0.8) | 3409 (0.7) |
| Number of previous tests | 0 | 736966 (83.3) | 737830 (83.4) | 359510 (75.8) | 353838 (74.6) |
|  | 1 | 105019 (11.9) | 104864 (11.9) | 69304 (14.6) | 71077 (15.0) |
|  | 2 | 23286 (2.6) | 23168 (2.6) | 17308 (3.7) | 18140 (3.8) |
|  | 3 | 7146 (0.8) | 7263 (0.8) | 5359 (1.1) | 5842 (1.2) |
|  | 4-9 | 8555 (1.0) | 8230 (0.9) | 8668 (1.8) | 9980 (2.1) |
|  | 10+ | 3245 (0.4) | 2862 (0.3) | 13920 (2.9) | 15192 (3.2) |
| BMI | Underweight | 8370 (0.9) | 8516 (1.0) | 3909 (0.8) | 3840 (0.8) |
|  | Normal weight | 105674 (12.0) | 108779 (12.3) | 61058 (12.9) | 61057 (12.9) |
|  | Overweight | 570805 (64.6) | 567369 (64.2) | 308758 (65.1) | 308999 (65.2) |
|  | Obese | 199368 (22.5) | 199553 (22.6) | 100344 (21.2) | 100173 (21.1) |
| Number of people in household | 1 | 307220 (34.7) | 303763 (34.4) | 143218 (30.2) | 142702 (30.1) |
|  | 2 | 299800 (33.9) | 299040 (33.8) | 160810 (33.9) | 160979 (34.0) |
|  | 3-5 | 260189 (29.4) | 264563 (29.9) | 159190 (33.6) | 159661 (33.7) |
|  | 6-10 | 16267 (1.8) | 16140 (1.8) | 10308 (2.2) | 10192 (2.1) |
|  | 11-30 | 640 (0.1) | 640 (0.1) | 456 (0.1) | 458 (0.1) |
|  | 31-100 | 82 (0.0) | 60 (0.0) | 69 (0.0) | 64 (0.0) |
|  | 101+ | 19 (0.0) | 11 (0.0) | 18 (0.0) | 13 (0.0) |
| † Deprivation status: Quintiles of the Scottish Index of Multiple Deprivation (SIMD) 2020  ‡ Number of risk groups: Count of QCovid risk groups: [doi:10.1136/bmj.m3731](https://www.bmj.com/content/371/bmj.m3731) | | | | | |

**Table S5g: Wales first dose matching summary table**

| **Characteristic** | **Level** | **Unvaccinated controls ChAdOx1** | **First dose recipient ChAdOx1** | **Unvaccinated controls BNT162b2** | **First dose recipient BNT162b2** |
| --- | --- | --- | --- | --- | --- |
| Total |  | 499,386 | 499,386 | 413,318 | 413,318 |
| COVID-19 hospitalisation or death | | 367 (10) | 358 (4) | 310 (10) | 125 (2) |
| Sex | Male | 261,291 (52) | 244,711 (49) | 198,150 (48) | 184,305 (45) |
|  | Female | 238,095 (48) | 254,675 (51) | 215,168 (52) | 229,013 (55) |
| Age group (years) | 18-64 | 348,296 (70) | 348,296 (70) | 324,951 (79) | 324,951 (79) |
|  | 65-79 | 97,273 (20) | 97,273 (20) | 82,704 (20) | 82,704 (20) |
|  | 80-110 | 53,817 (11) | 53,817 (11) | 5,663 (1) | 5,663 (1) |
| Deprivation status † | 1 | 98,768 (20) | 97,352 (20) | 88,730 (22) | 82,680 (20) |
|  | 2 | 100,838 (20) | 102,205 (20) | 87,635 (21) | 84,626 (20) |
|  | 3 | 98,634 (20) | 98,072 (20) | 80,958 (20) | 75,732 (18) |
|  | 4 | 95,948 (19) | 98,239 (20) | 74,999 (18) | 75,841 (18) |
|  | 5 | 105,198 (21) | 103,518 (21) | 80,996 (20) | 94,439 (23) |
| Urban/rural classification | Rural town and fringe | 66,962 (13) | 70,169 (14) | 56,726 (14) | 56,420 (14) |
|  | Rural town and fringe in a sparse setting | 16,286 (3) | 16,971 (3) | 12,211 (3) | 9,826 (2) |
|  | Rural village and dispersed | 30,837 (6) | 30,645 (6) | 21,871 (5) | 21,989 (5) |
|  | Rural village and dispersed in a sparse setting | 37,913 (8) | 36,922 (7) | 24,918 (6) | 19,805 (5) |
|  | Urban city and town | 337,893 (68) | 336,024 (67) | 288,790 (70) | 298,005 (72) |
|  | Urban city and town in a sparse setting | 9,495 (2) | 8,655 (2) | 8,802 (2) | 7,273 (2) |
| Number of risk groups ‡ | 0 | 267,586 (54) | 231,121 (46) | 248,827 (60) | 251,164 (61) |
|  | 1 | 137,791 (28) | 149,524 (30) | 107,920 (26) | 111,573 (27) |
|  | 2 | 55,165 (11) | 68,124 (14) | 35,005 (8) | 32,784 (8) |
|  | 3 | 22,153 (4) | 29,261 (6) | 12,724 (3) | 10,827 (3) |
|  | 4 | 9,754 (2) | 12,730 (2) | 5,273 (1) | 4,161 (1) |
|  | 5+ | 6,937 (1) | 8,626 (2) | 3,569 (1) | 2,809 (1) |
| Number of previous tests | 0 | 422,043 (84) | 407,011 (82) | 312,139 (76) | 296,663 (72) |
|  | 1 | 57,848 (12) | 66,411 (13) | 65,537 (16) | 74,623 (18) |
|  | 2 | 11,551 (2) | 14,846 (3) | 17,414 (4) | 20,486 (5) |
|  | 3-4 | 4,279 (1) | 5,764 (1) | 6,921 (2) | 8,551 (2) |
|  | 5-9 | 1,768 (0) | 2,338 (0) | 3,441 (1) | 3,868 (1) |
|  | 10+ | 1,897 (0) | 3,016 (1) | 7,866 (2) | 9,127 (2) |
| BMI | 18.4 or less | 11,389 (2) | 9,806 (2) | 14,546 (4) | 11,493 (3) |
|  | 18.5-24.9 | 134,035 (27) | 127,758 (26) | 135,665 (33) | 128,343 (31) |
|  | 25.0-29.9 | 165,643 (33) | 164,726 (33) | 132,917 (32) | 134,379 (32) |
|  | 30.0-39.9 | 188,319 (38) | 197096 (39) | 130,910 (31) | 139,103 (34) |
| Number of people in household | 1 | 86,350 (17) | 82,186 (16) | 46,661 (11) | 42,885 (10) |
|  | 2 | 157,490 (32) | 163,352 (33) | 107,451 (26) | 107,090 (26) |
|  | 3-5 | 222,653 (45) | 224,781 (45) | 219,449 (53) | 229,803 (56) |
|  | 6-10 | 29,914 (6) | 26,591 (5) | 36,494 (9) | 31,528 (8) |
|  | 11+ | 2,979 (1) | 2,476 (0) | 3,263 (1) | 2,012 (0) |
| † Deprivation status: Quintiles of the Welsh Index of Multiple Deprivation (SIMD) 2020  ‡ Number of risk groups: Count of QCovid risk groups: [doi:10.1136/bmj.m3731](https://www.bmj.com/content/371/bmj.m3731) | | | | | |

**Table S5h: Wales second dose matching summary table**

| **Characteristic** | **Level** | **First dose controls ChAdOx1** | **Second dose recipient ChAdOx1** | **First dose controls BNT162b2** | **Second dose recipient BNT162b2** |
| --- | --- | --- | --- | --- | --- |
| Total |  | 73,193 | 73,193 | 143,415 | 143,415 |
| COVID-19 hospitalisation or death | | 25 (3) | 7 (1) | 50 (1) | 15 (0) |
| Sex | Male | 36,008 (49) | 34,103 (47) | 46,086 (32) | 43,839 (31) |
|  | Female | 37,185 (51) | 39,090 (53) | 97,329 (68) | 99,576 (69) |
| Age group | 18-64 | 67,875 (93) | 67,875 (93) | 133,863 (93) | 133,863 (93) |
|  | 65-79 | 2,907 (4) | 2,907 (4) | 8,720 (6) | 8,720 (6) |
|  | 80-110 | 2,411 (3) | 2,411 (3) | 832 (1) | 832 (1) |
| Deprivation status † | 1 | 18,810 (26) | 16,276 (22) | 25,439 (18) | 24,844 (17) |
|  | 2 | 16,572 (23) | 15,655 (21) | 28,380 (20) | 28,595 (20) |
|  | 3 | 13,581 (19) | 14,191 (19) | 26,041 (18) | 27,675 (19) |
|  | 4 | 12,731 (17) | 13,712 (19) | 27,626 (19) | 28,608 (20) |
|  | 5 | 11,499 (16) | 13,359 (18) | 35,929 (25) | 33,693 (24) |
| Urban/rural classification | Rural town and fringe | 10,402 (14) | 10,761 (15) | 19,820 (14) | 19,716 (14) |
|  | Rural town and fringe in a sparse setting | 1,986 (3) | 2,412 (3) | 3,484 (2) | 4,175 (3) |
|  | Rural village and dispersed | 3,872 (5) | 4,110 (6) | 8,227 (6) | 8,457 (6) |
|  | Rural village and dispersed in a sparse setting | 4,088 (6) | 4,621 (6) | 6,889 (5) | 8,584 (6) |
|  | Urban city and town | 51,424 (70) | 49,793 (68) | 102,621 (72) | 99,474 (69) |
|  | Urban city and town in a sparse setting | 1,421 (2) | 1,496 (2) | 2,374 (2) | 3,009 (2) |
| Number of risk groups ‡ | 0 | 38,886 (53) | 31,325 (43) | 90,273 (63) | 84,765 (59) |
|  | 1 | 23,657 (32) | 25,931 (35) | 39,119 (27) | 40,924 (28) |
|  | 2 | 7,275 (10) | 10,555 (14) | 10,075 (7) | 12,289 (9) |
|  | 3 | 2,041 (3) | 3,574 (5) | 2,607 (2) | 3,556 (2) |
|  | 4 | 742 (1) | 1,203 (2) | 827 (1) | 1,170 (1) |
|  | 5+ | 592 (1) | 605 (1) | 514 (0) | 711 (0) |
| Number of previous tests | 0 | 55,881 (76) | 52,261 (71) | 93,714 (65) | 87,624 (61) |
|  | 1 | 12,362 (17) | 13,246 (18) | 32,036 (22) | 32,251 (22) |
|  | 2 | 2,922 (4) | 3,654 (5) | 10,127 (7) | 10,510 (7) |
|  | 03-Apr | 1,076 (2) | 1,678 (2) | 4,021 (3) | 4,970 (4) |
|  | 05-Sep | 425 (1) | 859 (1) | 1,420 (1) | 2,680 (2) |
|  | 10+ | 527 (1) | 1,495 (2) | 2,097 (2) | 5,380 (4) |
| BMI | 18.4 or less | 2,419 (3) | 1,989 (3) | 2,837 (2) | 2,976 (2) |
|  | 18.5-24.9 | 22,921 (31) | 21,002 (29) | 41,050 (29) | 41,692 (29) |
|  | 25.0-29.9 | 22,672 (31) | 21,912 (30) | 46,432 (32) | 45,539 (32) |
|  | 30.0-39.9 | 25,181 (34) | 28,290 (39) | 53,096 (37) | 53,208 (37) |
| Number of people in household | 1 | 8,481 (12) | 7,150 (10) | 13,590 (10) | 13,220 (9) |
|  | 2 | 14,558 (20) | 15,364 (21) | 34,199 (24) | 34,000 (24) |
|  | 3-5 | 42,480 (58) | 43,667 (60) | 85,652 (60) | 85,435 (60) |
|  | 6-10 | 7,141 (10) | 6,470 (9) | 9,430 (7) | 10,124 (7) |
|  | 11+ | 533 (1) | 542 (1) | 544 (0) | 636 (0) |
| † Deprivation status: Quintiles of the Welsh Index of Multiple Deprivation (SIMD) 2020  ‡ Number of risk groups: Count of QCovid risk groups: [doi:10.1136/bmj.m3731](https://www.bmj.com/content/371/bmj.m3731) | | | | | |
