# Supplementary material for: Waning of first- and second-dose ChAdOx1 and BNT162b2 COVID-19 vaccinations: a pooled target trial study of 12.9 million individuals in England, Northern Ireland, Scotland and Wales
Source: Int J Epidemiol. 2022 Oct 22;52(1):22–31. doi: 10.1093/ije/dyac199 (PMC9620314; doi:10.1093/ije/dyac199)

**S6 Covariate balance.**

**Fig S6a.** Covariate balance plot for first-dose matching analysis in England


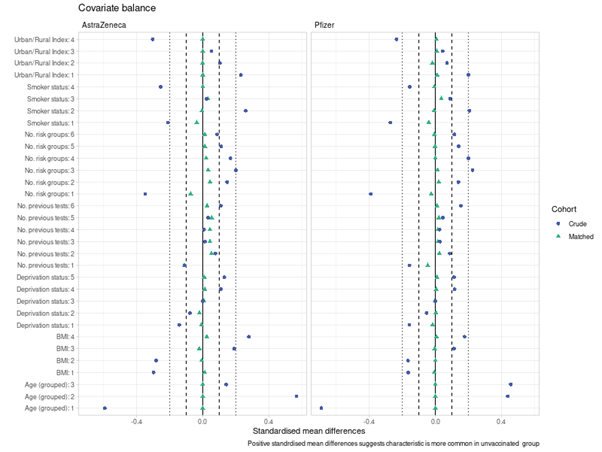


**Fig S6b.** Covariate balance plot for second-dose matching analysis in England


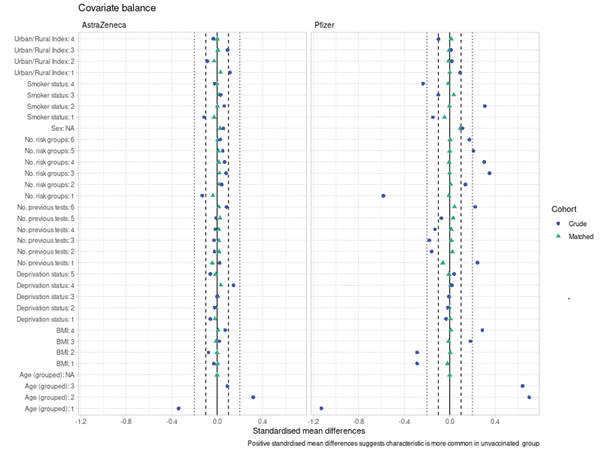


**Fig S6c.** Covariate balance plot for first-dose matching analysis in Northern Ireland


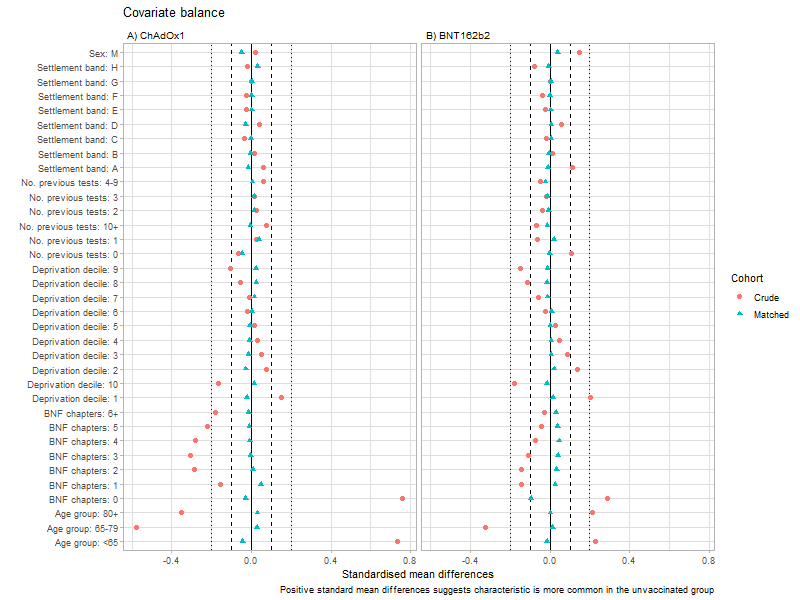


**Fig S6d.** Covariate balance plot for second-dose matching analysis in Northern Ireland


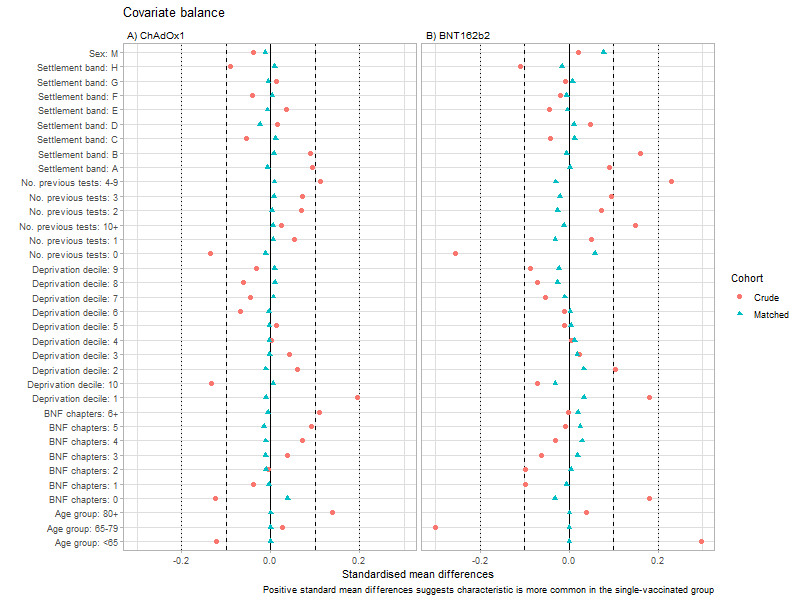


**Fig S6e.** Covariate balance plot for first-dose matching analysis in Scotland


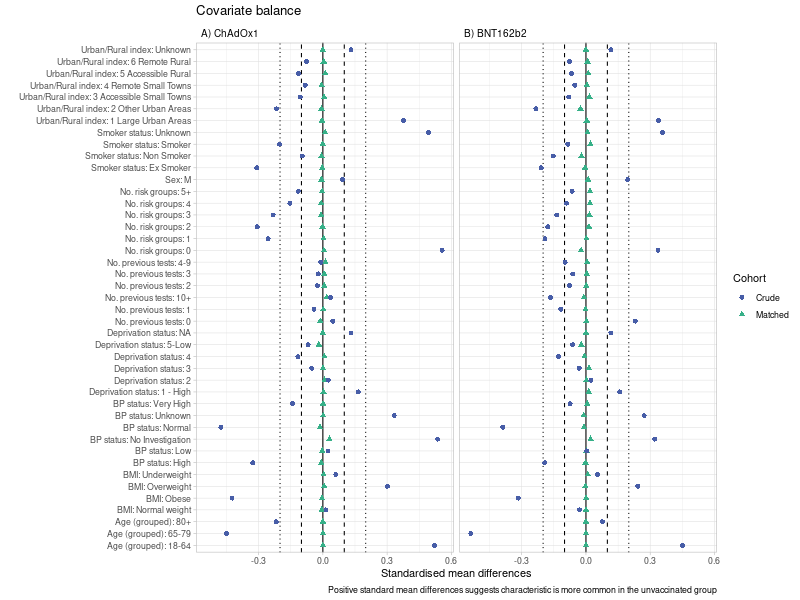


**Fig S6f.** Covariate balance plot for second-dose matching analysis in Scotland


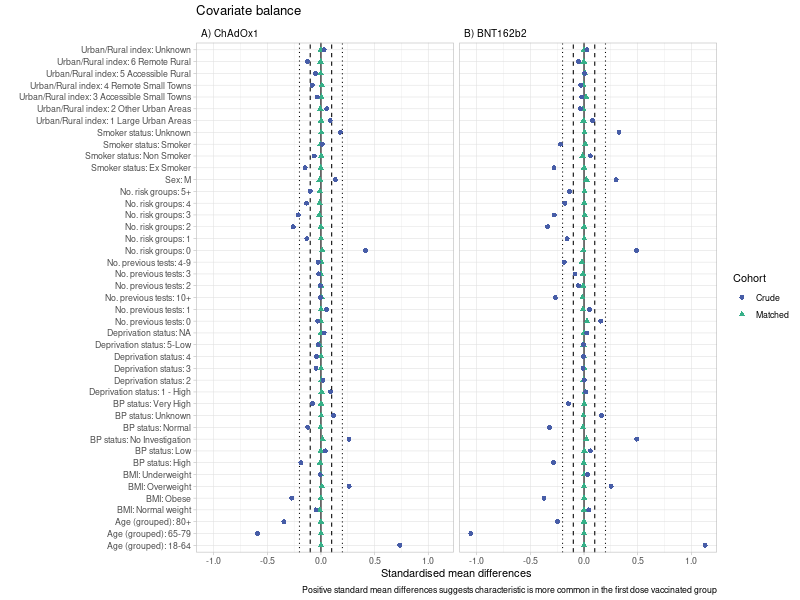


**Fig S6g.** Covariate balance plot for first-dose matching analysis in Wales


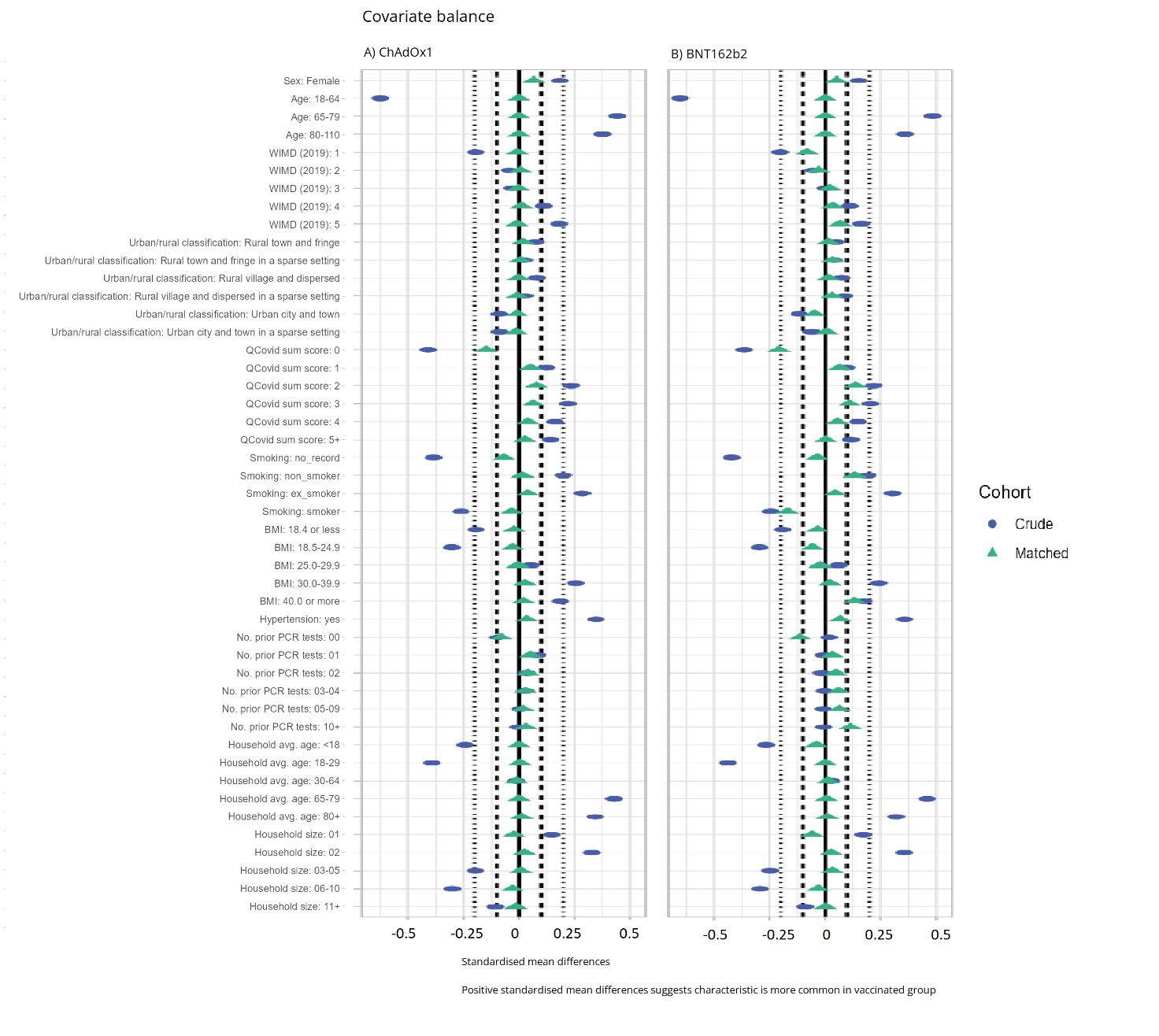


**Fig S6h.** Covariate balance plot for second-dose matching analysis in Wales


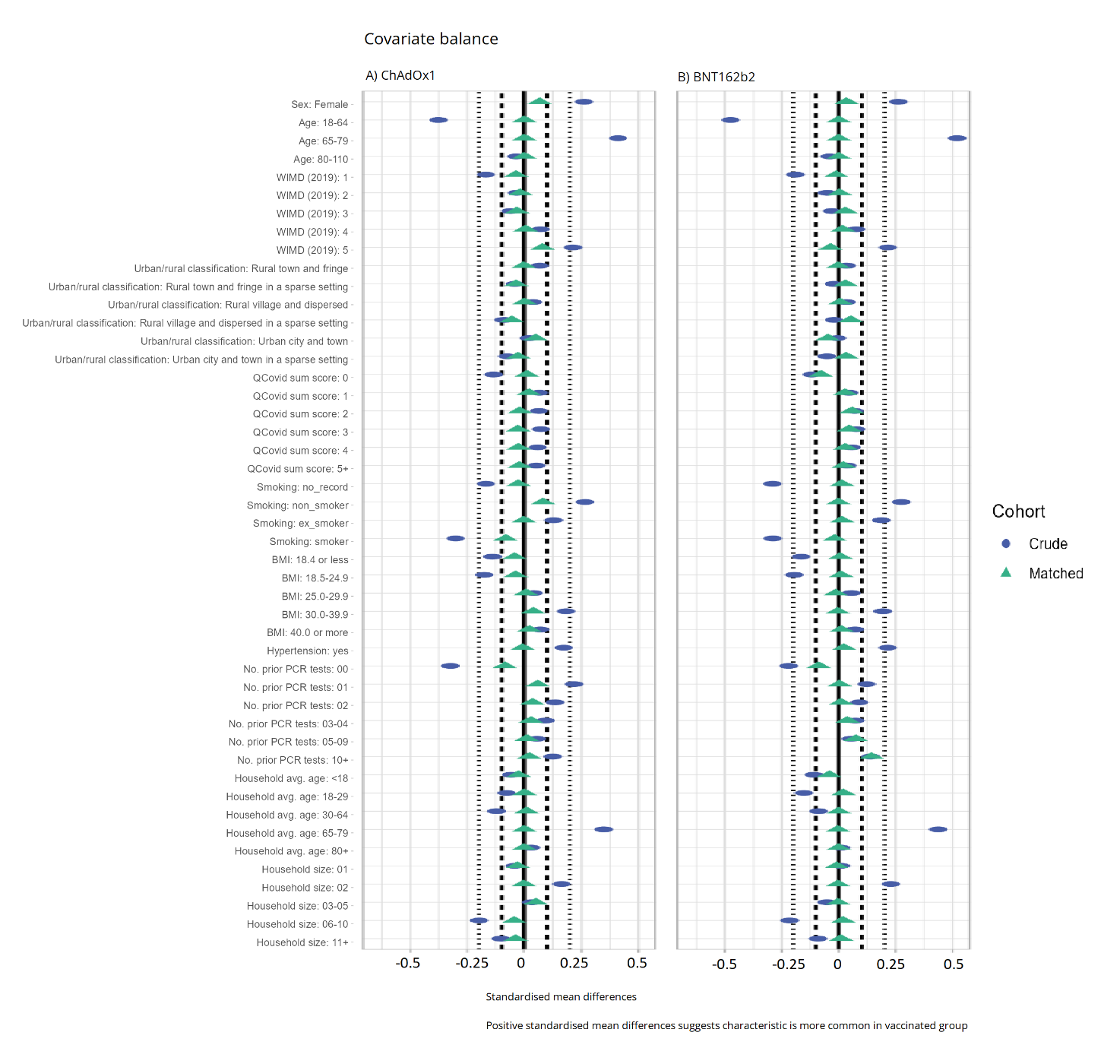

Supplement: dyac199_Supplementary_Data [file dyac199_supplementary_data.zip › dyac199_Supplementary_Data/ije-2022-04-0492-File013.docx]
