# Supplementary material for: Waning of first- and second-dose ChAdOx1 and BNT162b2 COVID-19 vaccinations: a pooled target trial study of 12.9 million individuals in England, Northern Ireland, Scotland and Wales
Source: Int J Epidemiol. 2022 Oct 22;52(1):22–31. doi: 10.1093/ije/dyac199 (PMC9620314; doi:10.1093/ije/dyac199)

**S7 Vaccine effectiveness by country.**

**Fig. S7a** Vaccine effectiveness first dose England


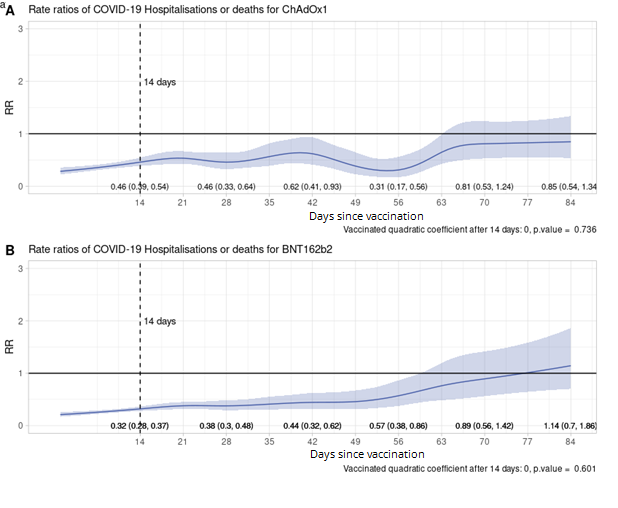


**Fig. S7b** Vaccine effectiveness second dose England


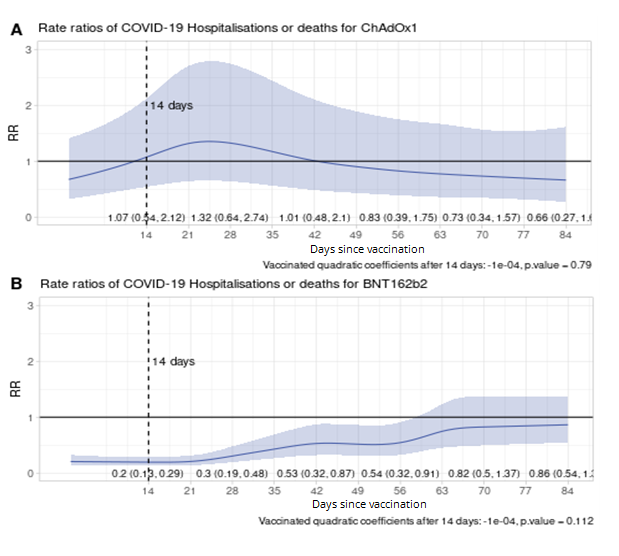


**Fig. S7c** Vaccine effectiveness first dose Northern Ireland


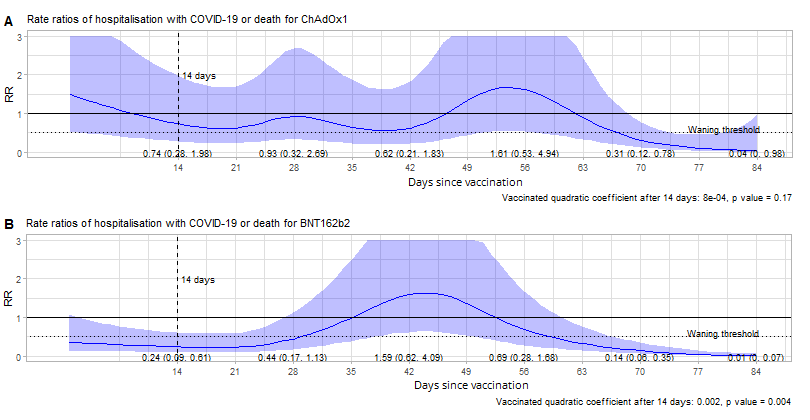


**Fig. S7d** Vaccine effectiveness second dose Northern Ireland


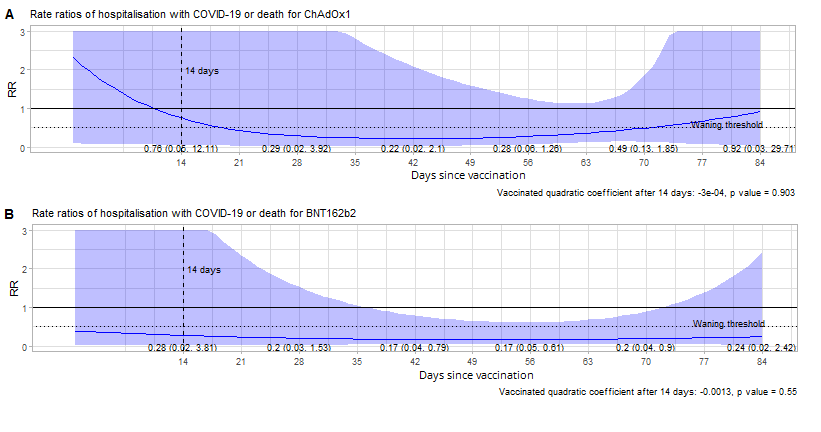


**Fig. S7e** Vaccine effectiveness first dose Scotland


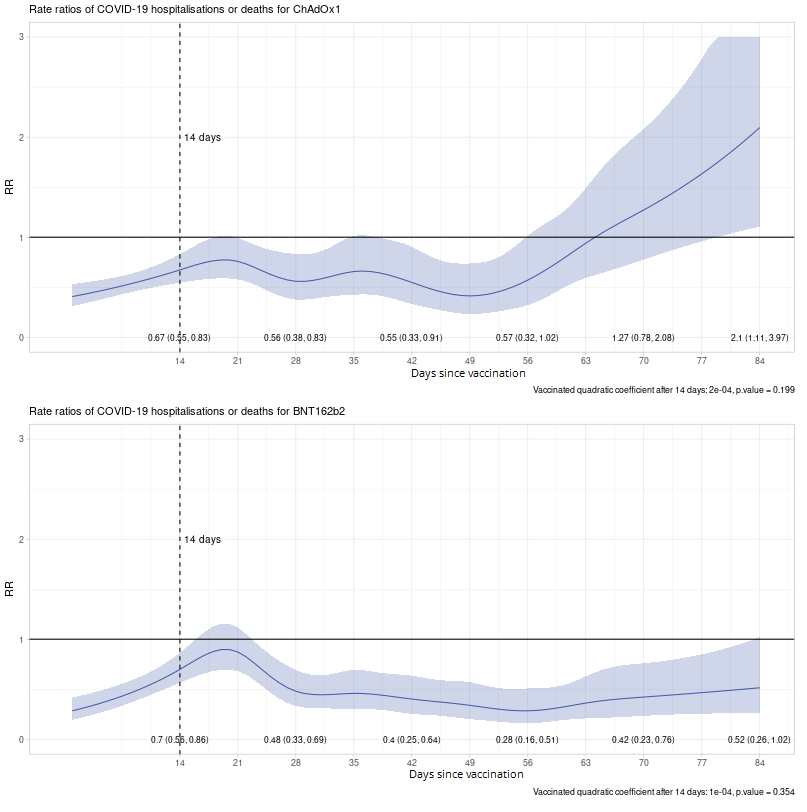


**Fig. S7f** Vaccine effectiveness second dose Scotland


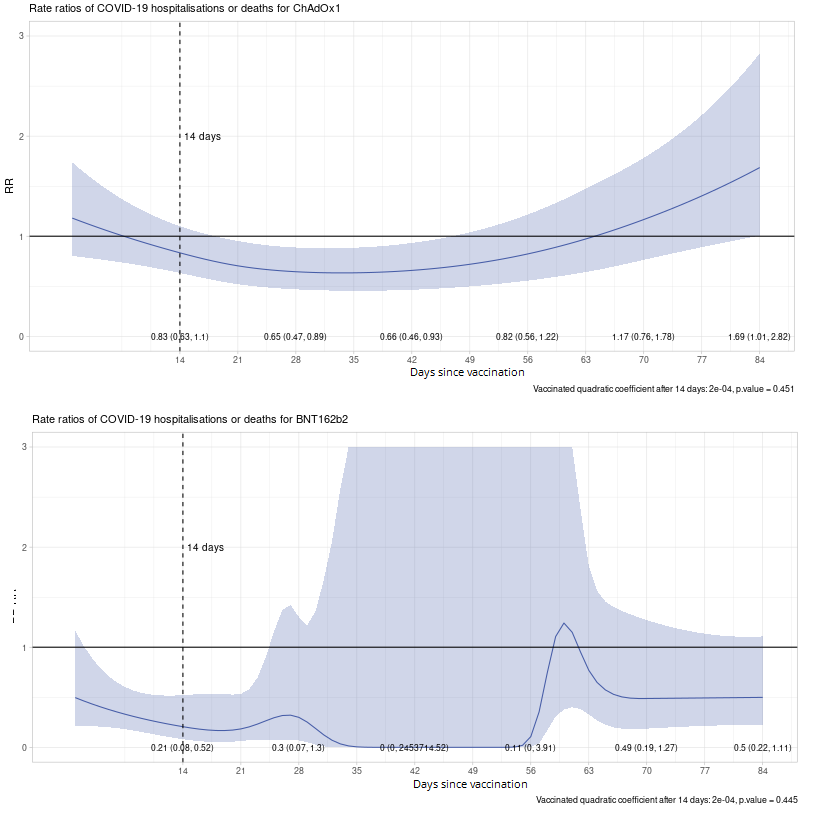


**Fig. S7g** Vaccine effectiveness Wales


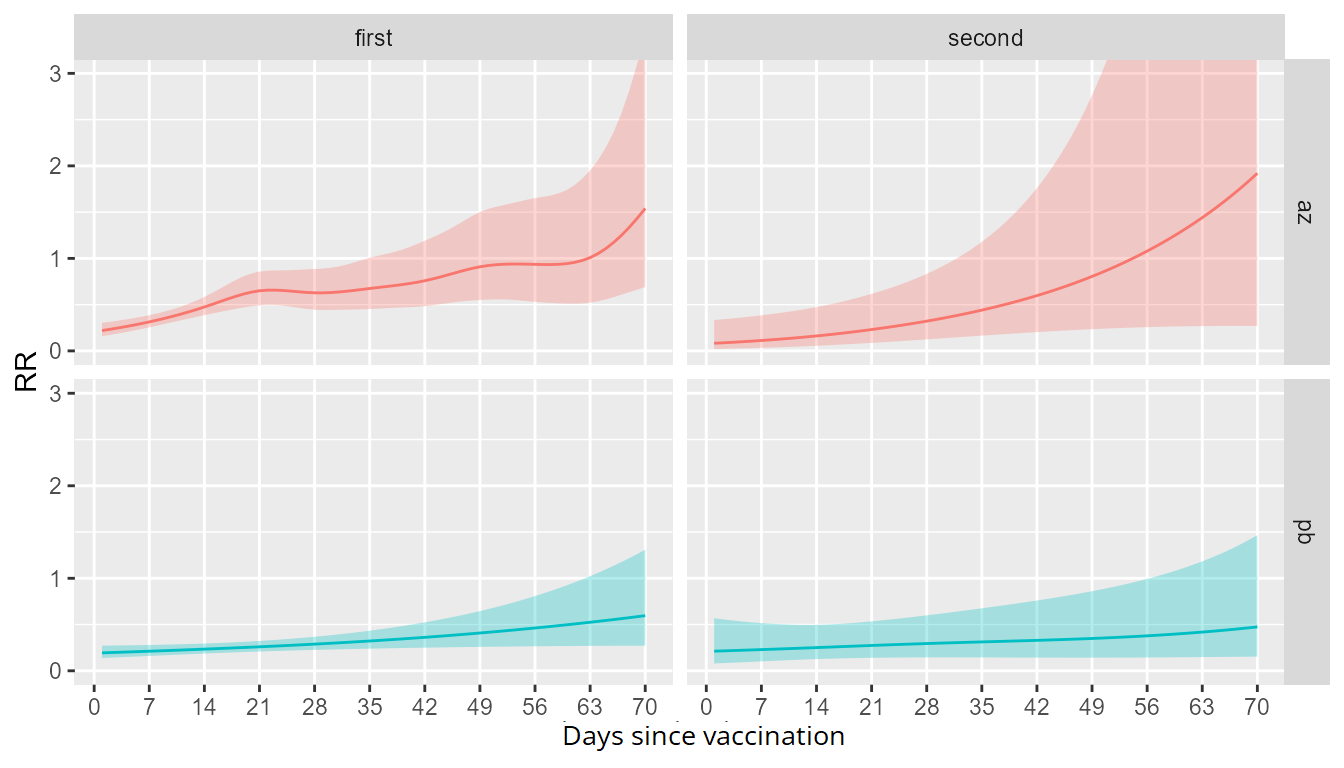

Supplement: dyac199_Supplementary_Data [file dyac199_supplementary_data.zip › dyac199_Supplementary_Data/ije-2022-04-0492-File014.docx]
