# Supplementary material for: Waning of first- and second-dose ChAdOx1 and BNT162b2 COVID-19 vaccinations: a pooled target trial study of 12.9 million individuals in England, Northern Ireland, Scotland and Wales
Source: Int J Epidemiol. 2022 Oct 22;52(1):22–31. doi: 10.1093/ije/dyac199 (PMC9620314; doi:10.1093/ije/dyac199)

**S8 Pooled vaccine effectiveness by age group**

**Fig. S8a** Pooled vaccine effectiveness first dose ChAdOx1 by age group


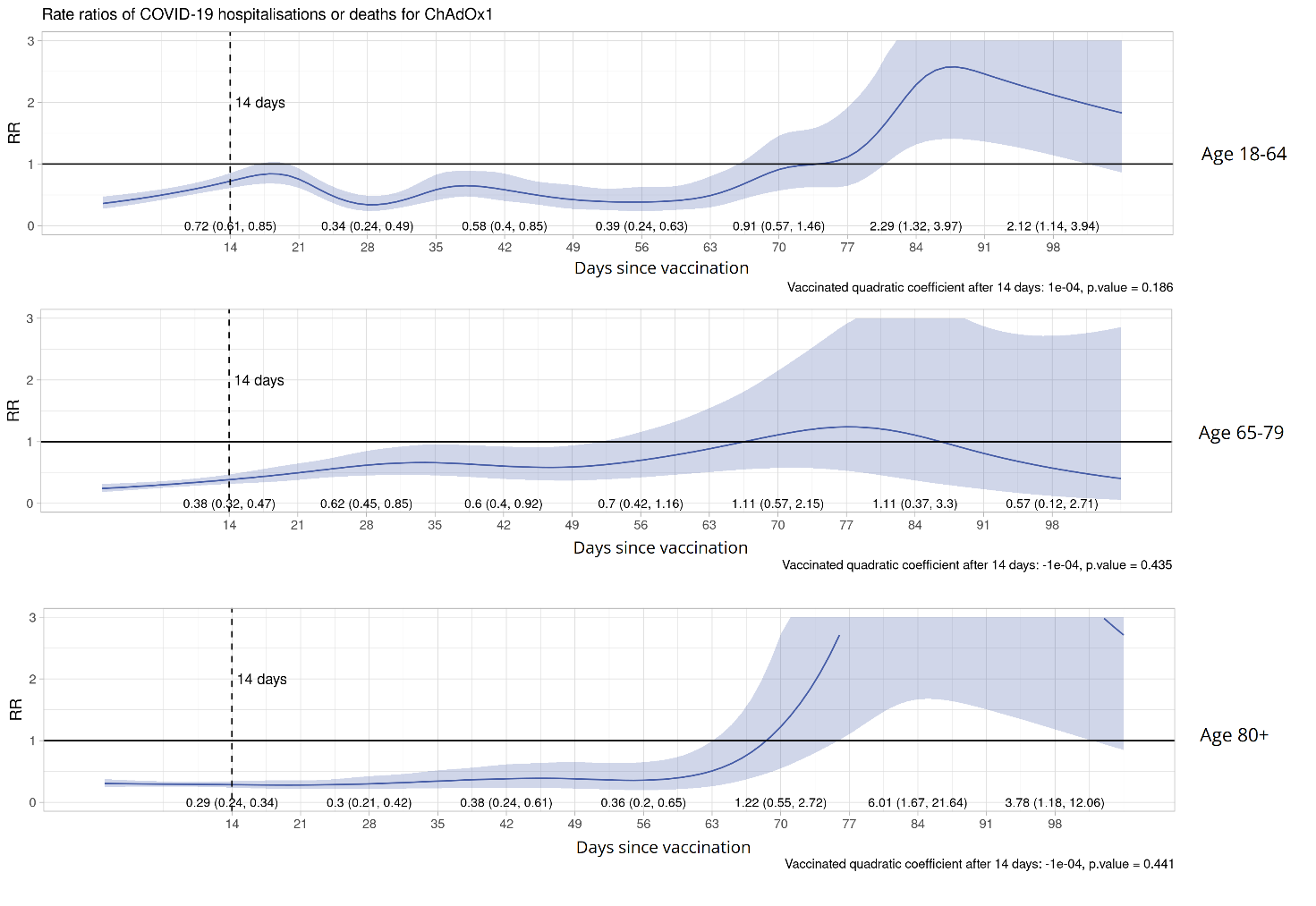


**Fig. S8b** Pooled vaccine effectiveness first dose BNT162b2age by age group


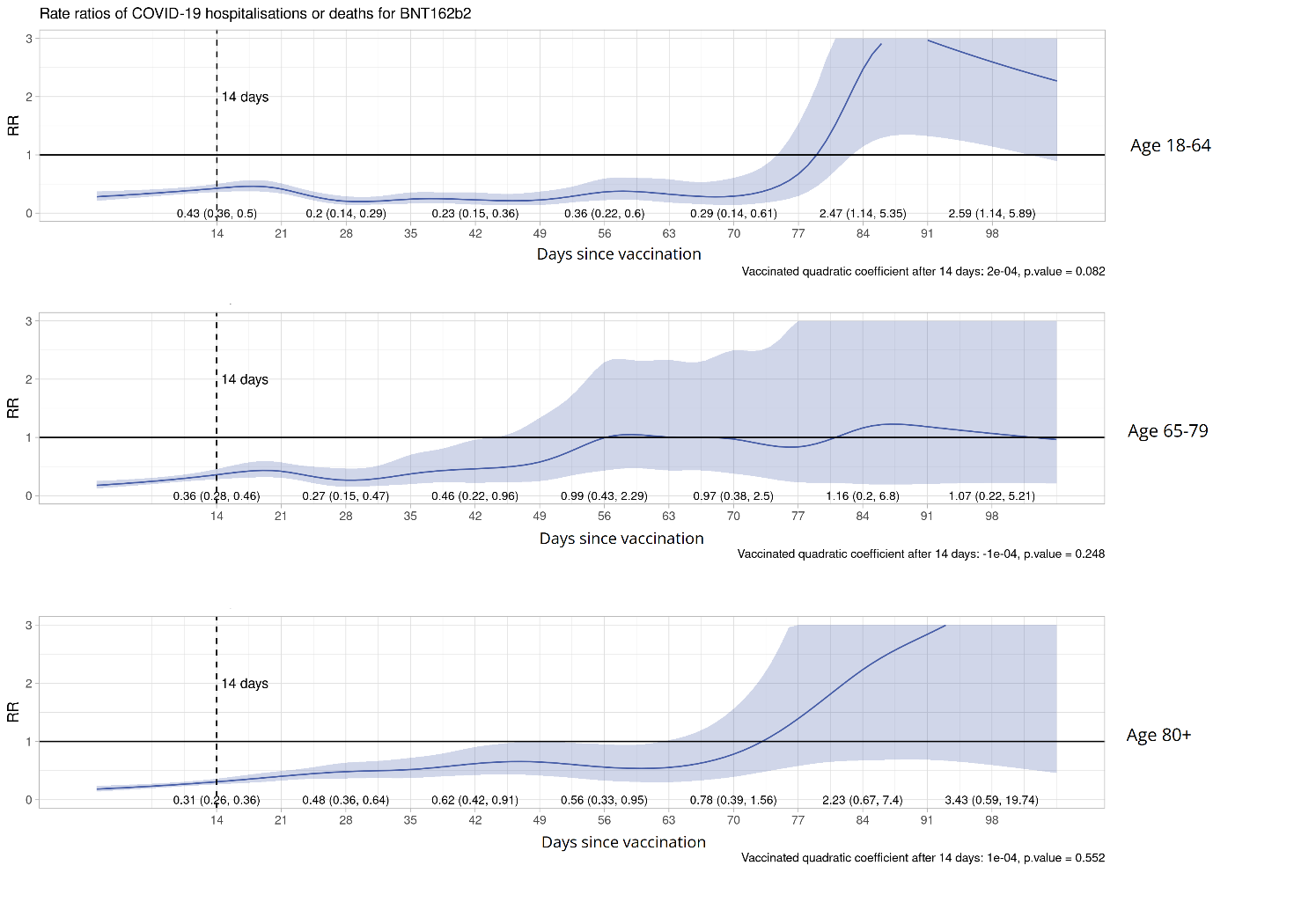


**Fig. S8c** Pooled vaccine effectiveness second dose ChAdOx1 by age group


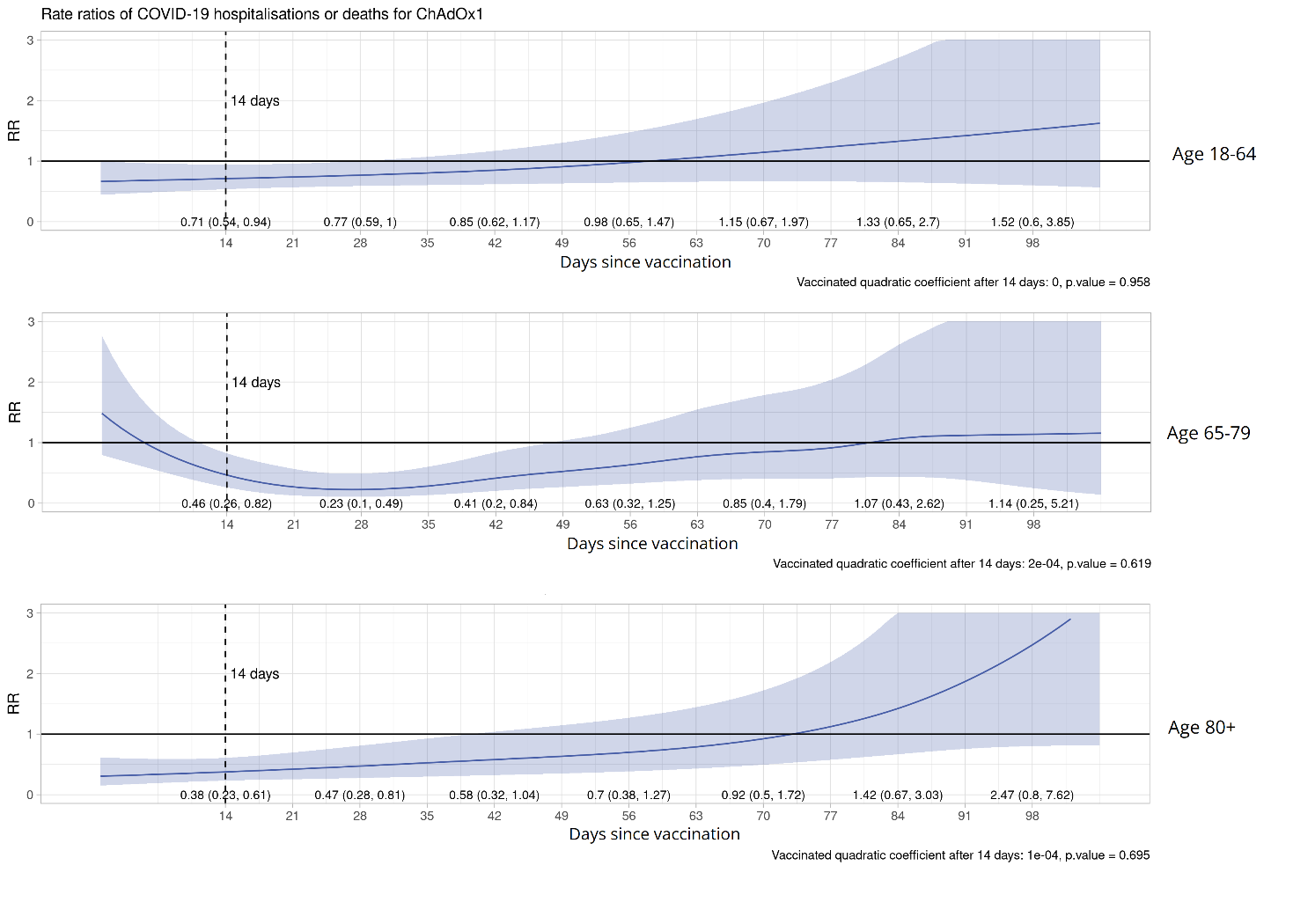


**Fig. S8d** Pooled vaccine effectiveness second dose BNT162b2age by age group


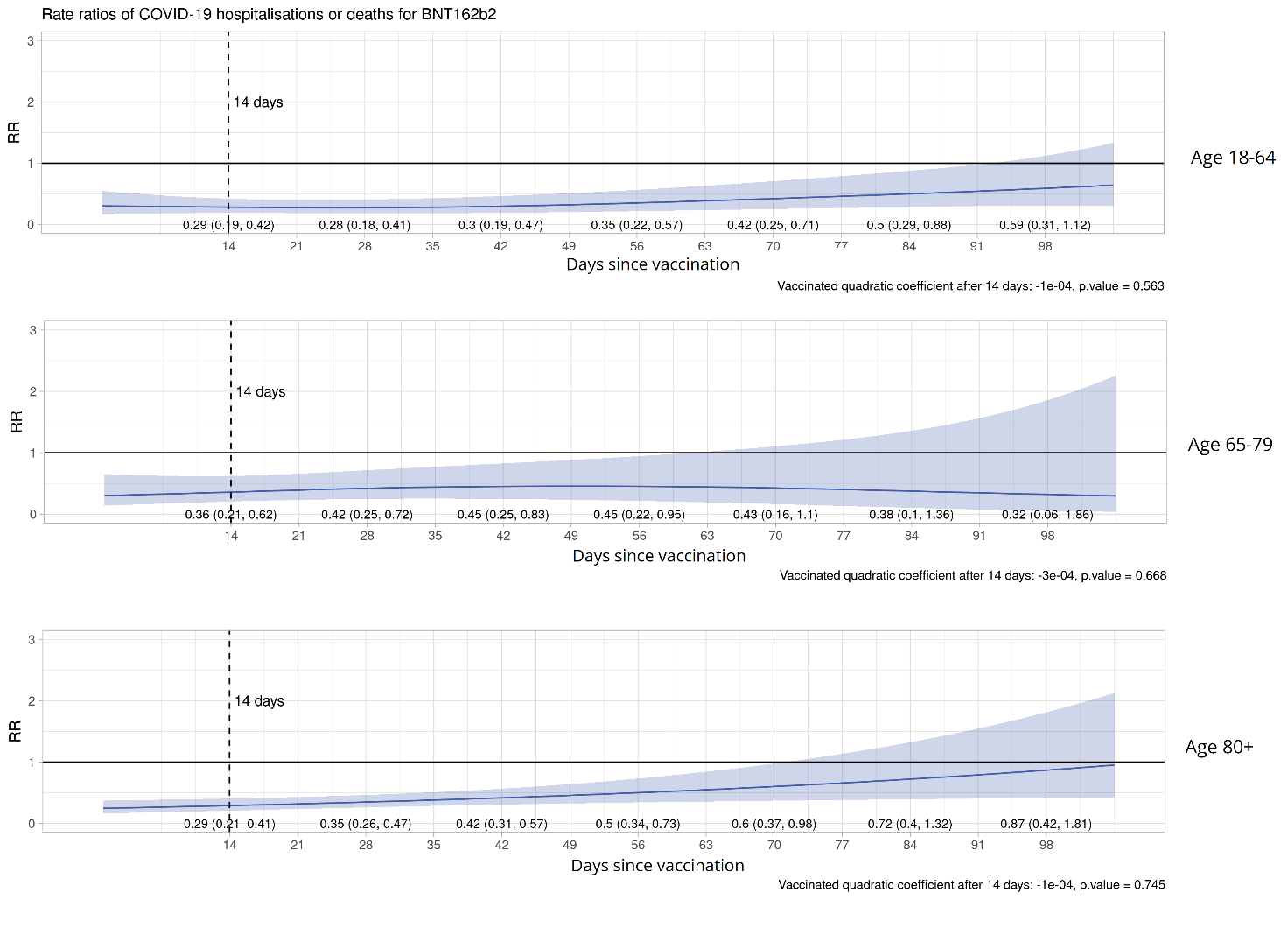

Supplement: dyac199_Supplementary_Data [file dyac199_supplementary_data.zip › dyac199_Supplementary_Data/ije-2022-04-0492-File015.docx]
